# The DP5 probability, quantification and visualisation of structural uncertainty in single molecules†

**DOI:** 10.1039/d1sc04406k

**Published:** 2022-02-25

**Authors:** Alexander Howarth, Jonathan M. Goodman

**Affiliations:** Centre for Molecular Informatics, Yusuf Hamied Department of Chemistry, University of Cambridge Lensfield Road Cambridge CB2 1EW UK jmg11@cam.ac.uk

## Abstract

Whenever a new molecule is made, a chemist will justify the proposed structure by analysing the NMR spectra. The widely-used DP4 algorithm will choose the best match from a series of possibilities, but draws no conclusions from a single candidate structure. Here we present the DP5 probability, a step-change in the quantification of molecular uncertainty: given one structure and one ^13^C NMR spectra, DP5 gives the probability of the structure being correct. We show the DP5 probability can rapidly differentiate between structure proposals indistinguishable by NMR to an expert chemist. We also show in a number of challenging examples the DP5 probability may prevent incorrect structures being published and later reassigned. DP5 will prove extremely valuable in fields such as discovery-driven automated chemical synthesis and drug development. Alongside the DP4-AI package, DP5 can help guide synthetic chemists when resolving the most subtle structural uncertainty. The DP5 system is available at https://github.com/Goodman-lab/DP5.

## Abstract 2

Whenever a new molecule is made, a chemist will justify the proposed structure by analysing the NMR spectra.

## Graphical abstract

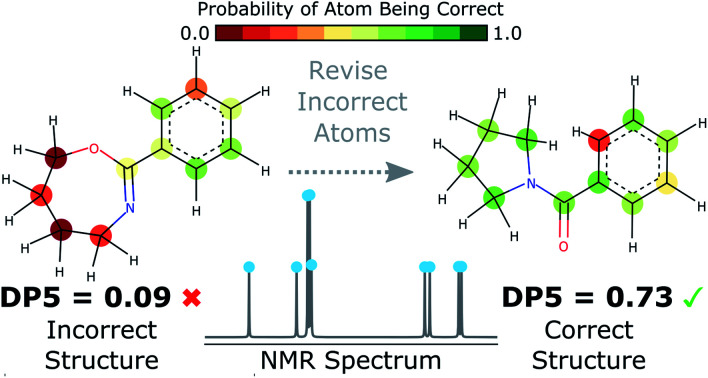

## Introduction

Molecular structure elucidation and verification are central problems in organic, synthetic and natural product chemistry. Due to the richness of the structural information its spectra contain, NMR spectroscopy has cemented itself as the method chemists use to solve these problems. Due to the complex nature of NMR spectra and often subtle variation between similar molecules, interpretation of these spectra can sometimes present a significant challenge. As a result, incorrectly assigned structures remain pervasive in the literature.^1^ Many of those incorrectly assigned are only discovered after costly and time consuming total syntheses are completed revealing a discrepancy between the experimental and literature NMR data.^2–4^

Over the last two decades, many computational tools have been developed to aid the assignment of NMR spectra and elucidation of molecular structures.^5–7^ Comparing experimental NMR shifts with those calculated for a candidate structure using density functional theory (DFT) is now a well-established methodology and has been used to solve the structures of many molecules.^8–11^ A powerful way of performing this analysis is to calculate the DP4 probability (and related metrics like DP4+ and J-DP4).^12–14^ Unlike comparative metrics such as MAE and CMAE, the DP4 algorithm applies Bayes Theorem to calculate the probability that each candidate structure is the correct one. DP4 requires a list of possible structures as its input, and it assumes that one of these structures is correct. It is common for structures to be determined except for uncertainty in the details of their stereochemistry. DP4 has proved invaluable in the resolution of many such cases.^15–19^ DP4 can also be used to resolve non-stereochemical uncertainty, provided that all of the acceptable possible structures can be enumerated. However, in cases where all the proposed candidate structures may be incorrect or only a single structure has been proposed, DP4 analysis cannot be applied. Until now in these very common situations chemists would have no quantitative way of assessing the probability of their proposed structure being correct given the NMR spectra.

To solve this problem, we present the DP5 probability, a new methodology and complete software package for quantifying uncertainty in molecular structures. Similar to the DP4 probability, the DP5 probability gives the probability that a candidate structure is correct. However, in contrast to DP4, DP5 calculates normalised stand-alone probabilities and hence, the user can propose one or many structures without having to assume any of their proposals are correct. As a result, DP5 can be used to answer different questions to DP4 and will prove valuable in situations where this type of analysis was previously impossible. The DP5 probability is calculated given only one-dimensional ^13^C NMR data and utilises the same computational engine as the latest iteration of our DP4 software, DP4-AI. This program manages all NMR processing, assignment, DFT calculations and statistical modelling automatically. DP5 can also be used on a case-by-case basis utilising the graphical user interface (GUI).

This work represents a great leap forward in this field, in previous works systems have been developed using Neural Networks (NNs) to classify structure proposals as correct or incorrect.^20^ These systems return binary metrics that cannot be interpreted as a probability, the statistical approach taken by DP5 solves the significantly more challenging problem of calculating a standalone normalised probability of a structure being correct. The NN systems are trained on vectors of metrics such as MAE and MSTD and hence repeat the analysis an expert chemist could perform. DP5 in contrast, takes into account the 3d geometry around each atomic environment to calculate the probability of observing the given NMR-DFT prediction error for each atom independently, this solves the well-known issue that DFT prediction errors vary in complex and non-linear ways with atomic environment. Finally, previous methods based on NNs are trained using a set of correct structure proposals and faked “incorrect” structure proposals. In contrast, the statistical approach taken by DP5 only utilises real data, avoiding utilising any fake datapoints and the subsequent effects of unbalanced training sets.

The system was developed and rigorously tested utilising a dataset of 5140 organic molecules from NMRShiftDB originally selected for NMR prediction using machine learning by Paton *et al.* (see ESI Section 6†).^21,22^ To demonstrate the performance of the DP5 probability in even more challenging situations, the system was also evaluated using 13 case studies of molecular structures that have undergone reassignments in the literature and addition 42 challenging relative stereochemistry elucidation examples.

DP5 represents an exciting leap forward in quantifying molecular uncertainty. This system will prove valuable in fields requiring high throughput molecular structure elucidation such as automated chemical synthesis, but also in traditional organic chemistry as a tool to aid and guide expert chemists in their development of complex syntheses. DP5 has been made possible following recent advances in molecular machine learning techniques and increased data availability.^23–28^

## Computational methods

DFT calculations for the structure reassignment and stereochemistry elucidation examples were performed using the method developed in previous works.^29–31^ All molecular mechanics calculations were performed using MacroModel (Version 9.9).^32^ All conformational searches were performed in the gas phase utilizing the MMFF force field^33–38^ and a mixture of Low Mode following and Monte Carlo search algorithms.^39,40^ The step count for MacroModel was set so that all low energy conformers were found at least 5 times. Quantum mechanical calculations were carried out using Gaussian09.^41^ NMR shielding constants were found using the GIAO method.^42–44^ The functional mPW1PW91 (ref. 45) was chosen with the 6-311G(d)^46,47^ basis set for NMR shift prediction as this has been shown to be optimal for DP4 calculation. For molecules containing iodine, the basis set def2-SVP^48,49^ was chosen. All DFT calculations were performed using the implicit PCM solvent model.^50^ The molecular geometries were also optimized at the DFT level of theory, this was performed using the B3LYP functional^51,52^ with the 6-31G(d) basis set. Finally, single-point energies were separately calculated using M06-2X^53^ functional and def2-TZVP^48,49^ basis set.

The calculations were managed by the DP4-AI^31^ Python script written in Python 3.7. DP4-AI is available from http://www-jmg.ch.cam.ac.uk/tools/nmr/ and GitHub https://github.com/Goodman-lab/.

DFT optimised geometries and NMR shift calculations for the molecules from NMRShiftDB were obtained from the training data of the GNN NMR shift prediction software CASCADE.^22^ A single conformer of each of these molecules was optimised utilising the M062X functional and def2-TZVP basis set and NMR shift calculations performed using in 6-311g(d) basis set and mPW1PW91 functional.

Calculation of FCHL atomic representations, l2 distances and Gaussian kernel transformations were performed using the python package qml.^54^

### Program description

**Fig. 1:**
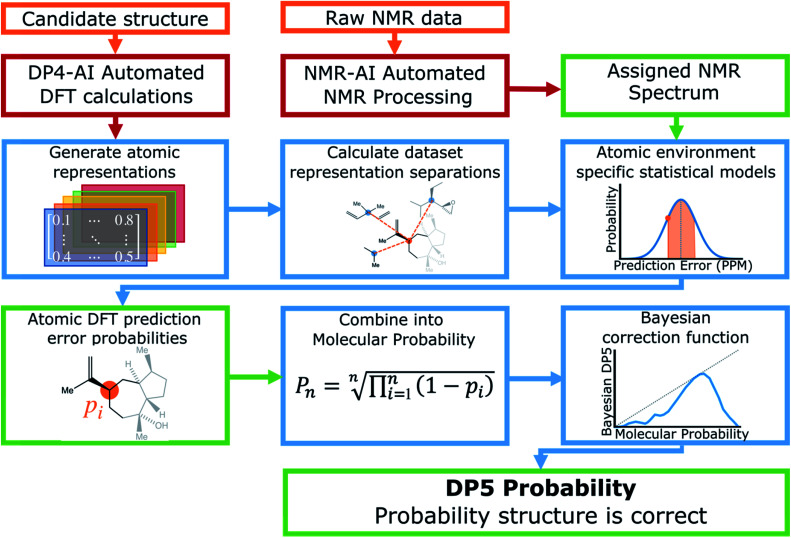
Schematic of the DP5 program. The required inputs from the user are a candidate structure and the raw ^13^C NMR data (or a list NMR signals). The DP5 probability is built on top of the DP4-AI analysis.

A schematic of the DP5 program is displayed in Fig. 1. Structure inputs can be made as any combination of, .sdf files, SMILES, SMARTS or InChIs. ^13^C NMR data can be input as raw data (for automated analysis) or as a list of peaks from a user analysis.

DP5 calculates NMR shifts for the atoms in populated conformers of the candidate structure utilising the highly optimised and well established methods within DP4-AI (see ESI Section 2.1†).^29–31^

Raw NMR data interpretation is handled by a part of DP4-AI called NMR-AI.^31^ This system was developed to remove the requirement for the user to process and assign NMR spectra and has been demonstrated to complete this task to at least the same high standard as an expert chemist.1
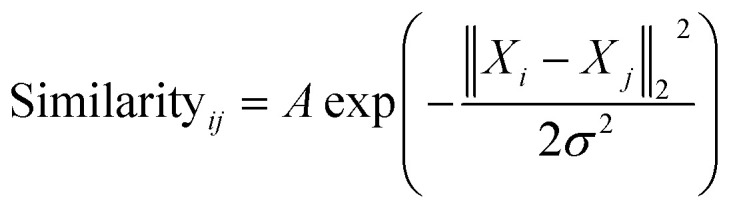


The similarity between the FCHL representation of atom *i*,*X*_*i*_ and that of atom *j* in the test set *X*_*j*_ is calculated using a Gaussian kernel.2
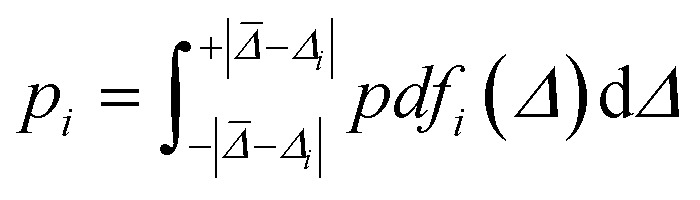


A prediction error probability for atom *i* is calculated by integrating the bespoke prediction error function generated for that atom, where *Δ*_*i*_ is the (internally scaled) prediction error for atom *i* and *Δ̄* corresponds to the mean absolute prediction error for the training set.

**Fig. 2:**
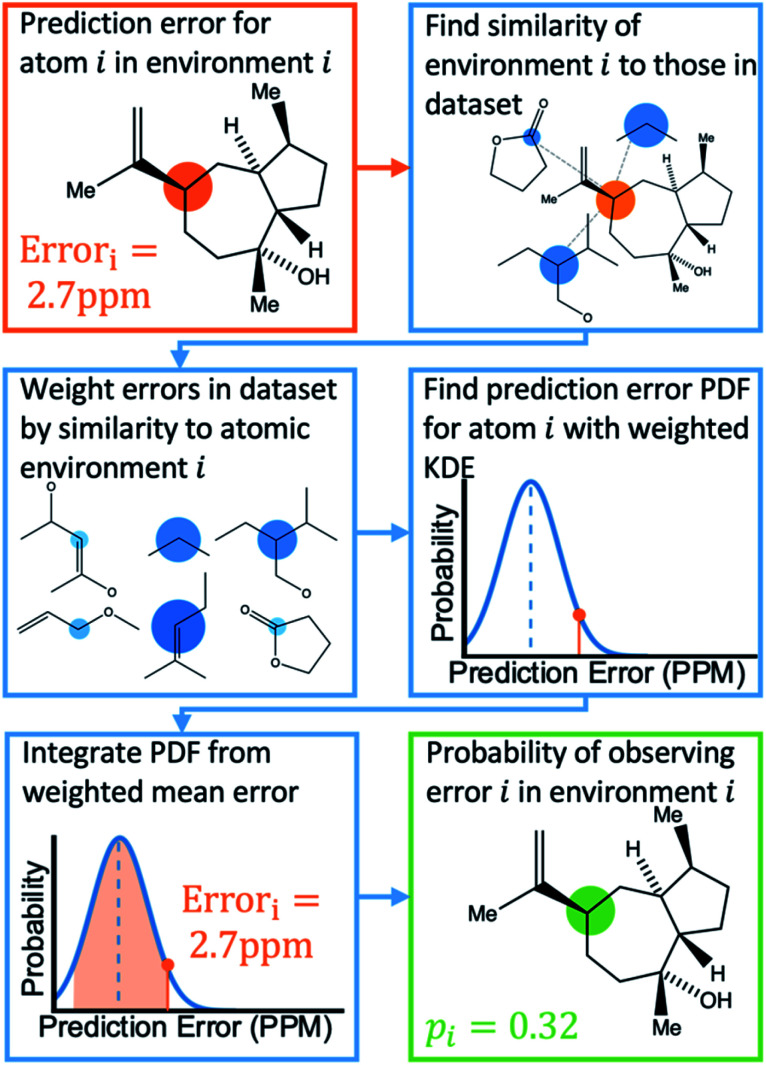
Schematic diagram of how the probability of observing a DFT-NMR prediction error for an atom in a given environment is calculated as described in the text.

Once the geometries of populated conformers have been calculated, the probabilities of the observed DFT-NMR prediction errors for each atom in that conformer can be found. To do this a probability density function (PDF) describing the DFT-NMR prediction error distribution for that atomic environment is required (see ESI Section S2.2†). This PDF is found empirically by performing a Kernel Density Estimation (KDE) on a dataset of 63 542 known prediction errors calculated for the DFT optimised geometries of 5140 molecules from NMRShiftDB. This dataset was originally developed for training machine learning models for NMR shift prediction, the generality and near chemical accuracy achieved by these models has been taken as justification for using this dataset in this similar task (details regarding this dataset can be found in the original publication).^22^ It is well known that the expected magnitude and variance of DFT prediction errors for different functionals show strong complex, nonlinear dependencies on atomic environment.^55,56^ This process takes this into account by weighting the contribution to the error PDF for the test atom of each atomic environment in the database by its similarity to the test environment. The similarity of these atomic environments is calculated by finding the Euclidian distance between a vector representation of the test atomic environment and those in the training set. These distances are converted into covariances utilising a Gaussian kernel (eqn (1); see ESI Section S2.4†). By setting the pre-exponential scaling factor to one, these covariances can be interpreted as a measure of the similarity. The resulting PDF is integrated by eqn (2) (see ESI Section S2.5†) to yield a prediction error probability for the test atom. This process is then repeated for each atom in each conformer of the proposed structure. Once atomic probabilities have been calculated for each atom in each conformer, these values are Boltzmann weighted to produce overall atomic probabilities for the structure. This process is summarised in Fig. 2.

The atomic representation used by DP5 was investigated in great detail. In recent years many representations have been developed for applications in molecular machine learning, such as the coulomb matrix,^57^ bag-of-bonds,^58^ aSLATM^59^ and FCHL.^27^ Kernel ridge regression (KRR) utilising the FCHL atomic representation have been shown to predict NMR shielding constants with near chemical accuracy (also tested in this work see ESI Section S3.4.1†).^60^ These works demonstrate that FCHL contains the information required to accurately encode atomic environments. Due to the similarity of these tasks, the FCHL representation has been chosen for use in the DP5 probability calculation (see ESI Section S2.3†).

A particular challenge in the development of the DP5 probability was determining an equation to combine individual atomic probabilities to yield probability for the whole structure. If any single atom is given too much influence, a molecular probability of one or zero will usually be assigned, whilst if there is too much smoothing over individual atomic probabilities, the resulting molecular probabilities will not show enough useful variation. A number of formulae were tested during this study (see ESI Section S2.7†). Overall eqn (3) was found to yield useful variation in molecular probabilities, whilst combining the atomic probabilities in a mathematically meaningful way. The inclusion of the geometric mean in eqn (3) was found to be necessary to prevent single atoms with a very high or low probability having too much influence on the final result.

The last stage in the calculation scales the molecular probability using a Bayesian correction function to yield the final DP5 probability (see ESI Section S2.8†). This empirical stage of the process ensures the DP5 probability assigned matches the probability of the structure being correct as closely as possible. This empirical correction function was found by first calculating a PDF for the molecular probabilities assigned to the 5140 NMRShiftDB molecules. By finding all the possible pairs of spectra and structures in this dataset with the same number of carbon atoms, a PDF of the molecular probabilities of incorrect spectra-structure pairs was also generated. In this instance, each pair was assigned a weight to ensure the mean absolute DFT-NMR prediction error distribution of these incorrect pairs matched that of the correct structure-spectra pairs (see ESI Section S3.2†). Given any proposed structure must be either correct or incorrect, by applying Bayes Theorem the DP5 probability is defined by eqn (4).3
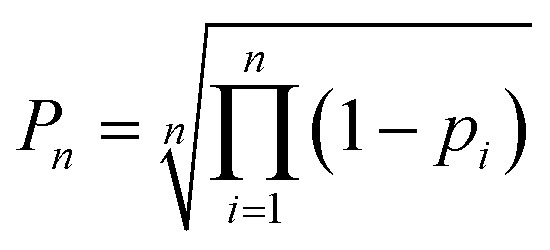


Atomic DP5 probabilities are combined to form the molecular probability *P*_*n*_ by eqn (3), where *n* is the number of atoms in the molecule and *p*_*i*_ is the DFT-NMR prediction error probability for atom *i*4
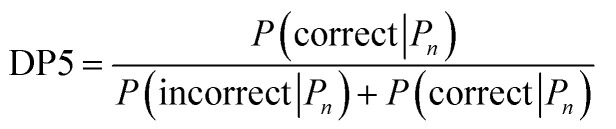


The DP5 probability is calculated by applying Bayes Theorem to the molecular probability calculated in eqn (3), where *P*(correct|*P*_*n*_) gives the probability of a molecule being correct given its calculated molecular probability *P*_*n*_.

**Fig. 3:**
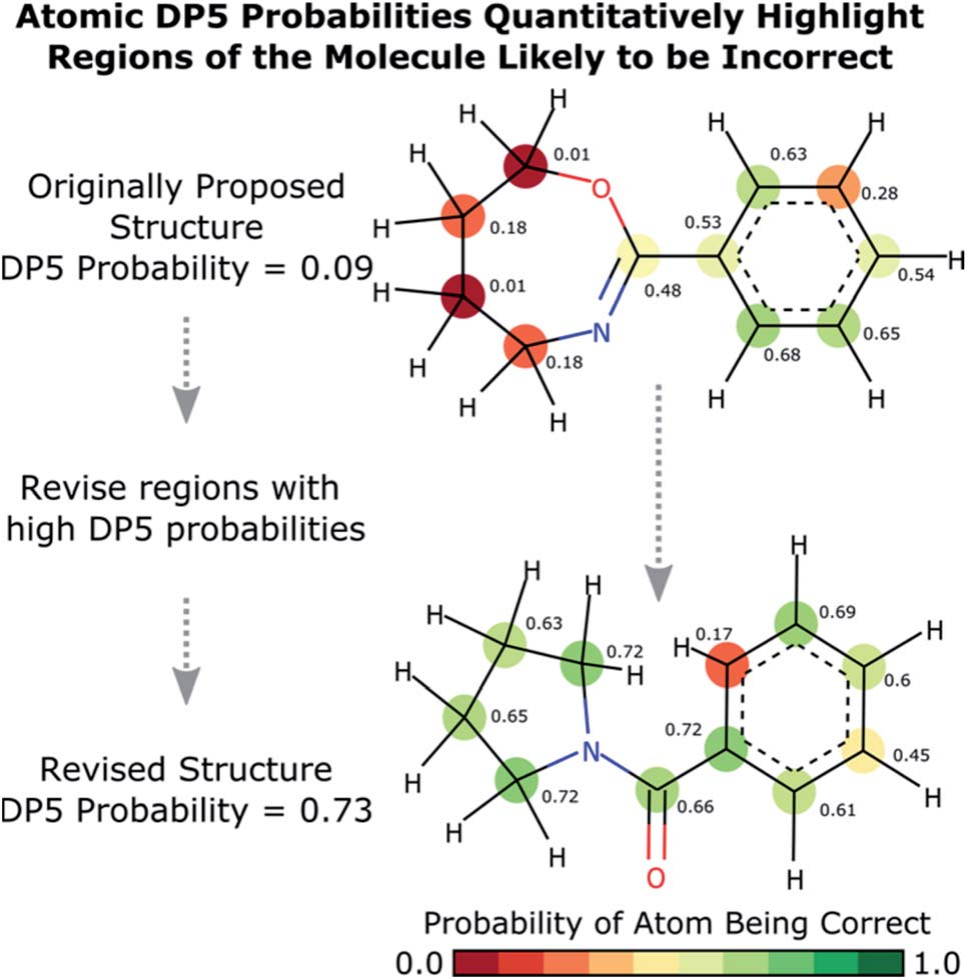
The GUI accompanying DP5 pictorially overlays atomic DP5 probabilities onto the molecular structure. This clearly displays regions of the structure that are expected to be correct and conversely regions that may require revision. This functionality will help chemists assess and revise structure proposals. This structure revision example has been taken from a real-world case study of an incorrectly assigned molecule in the literature (see Results).

Calculation of the DP5 probability has been integrated into the well-established DP4-AI workflow.^31^ All the required calculations and analysis of NMR data can be performed automatically with no user input required. DP5 can hence be integrated into pre-existing automatic reaction/characterisation workflows. DP5 analysis can also be performed on single molecules with the GUI. This GUI can be used to launch calculations, analyse NMR assignments made by NMR-AI and also to investigate the DP5 statistics. The GUI visually displays the atomic probabilities, helping the chemist identify potential regions of the molecule that may be incorrect and determine possible modifications (Fig. 3).

## Results

A major challenge in the development of DP5 involved constructing a method to assess the efficacy of the system. As the DP5 probability is not a physical property that can be measured, it is not straightforward to compare the DP5 probability assigned to a molecule with an experimental value. In this study two rigorous evaluation methods were devised to assess and improve the real-world effectiveness of the DP5 probability.

**Fig. 4:**
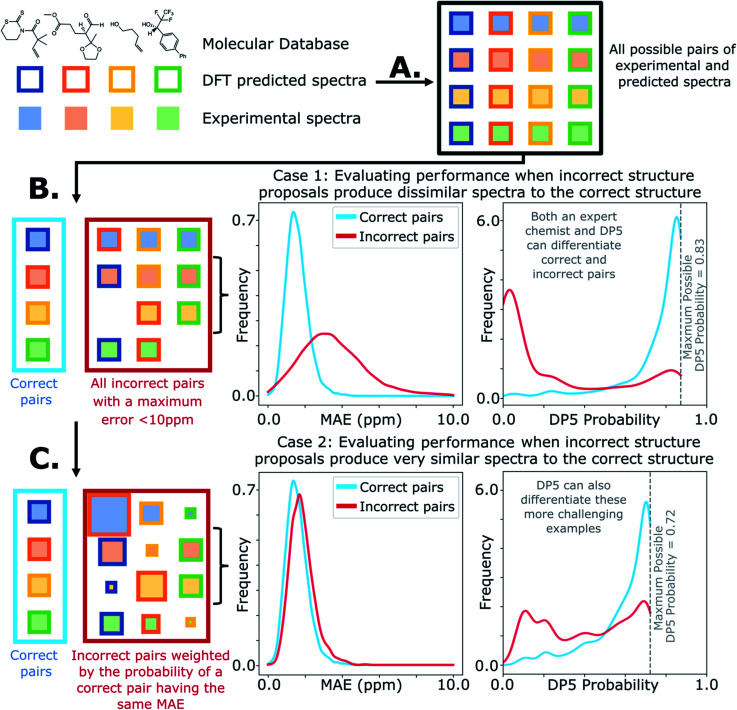
Schematic diagram of cross validation analysis used to evaluate the performance of DP5. (A) The experimental spectra of the 5140 (*n*) molecules from the NMRShiftDB training set with the same number of carbon atoms are permuted to produce pairs of structures and experimental spectra. (B) These pairs are separated into correct pairs, where the structure is paired to the correct spectrum and incorrect pairs where the molecule has been paired with a different spectrum. All incorrect pairs with max errors <10 ppm are considered in case 1. (C) In case 2 the incorrect pairs are assigned sampling weights to force the MAE distribution of the incorrect pairs to approximate that of the correct pairs, this leads to an expectation number of ∼5330 incorrect combinations. All DP5 probabilities in this study are calculated using a leave-one-out scheme (see ESI Section S3.2†).

**Fig. 5:**
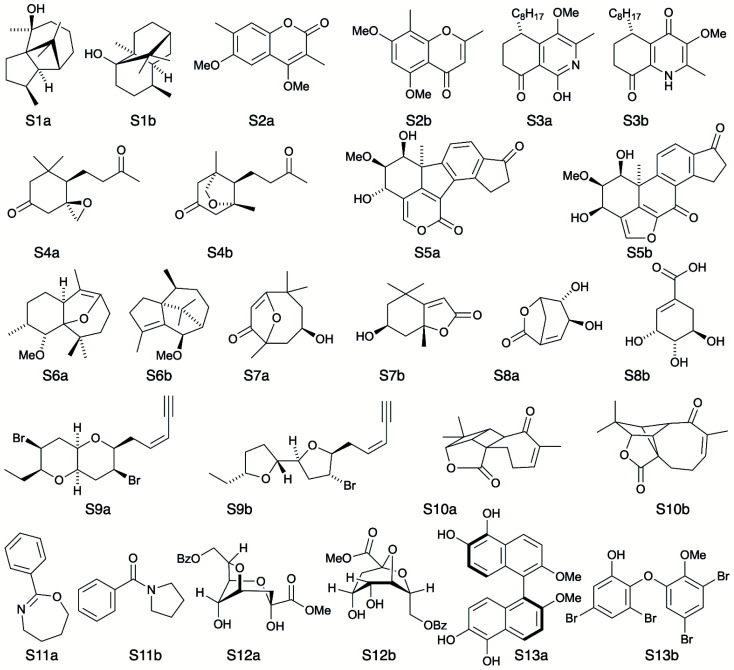
Test set of real-world structure reassignment problems taken from chemical literature. In each example an incorrect structure was initially published (S#a) which was later reassigned to the corresponding correct structure (S#b).

**Fig. 6:**
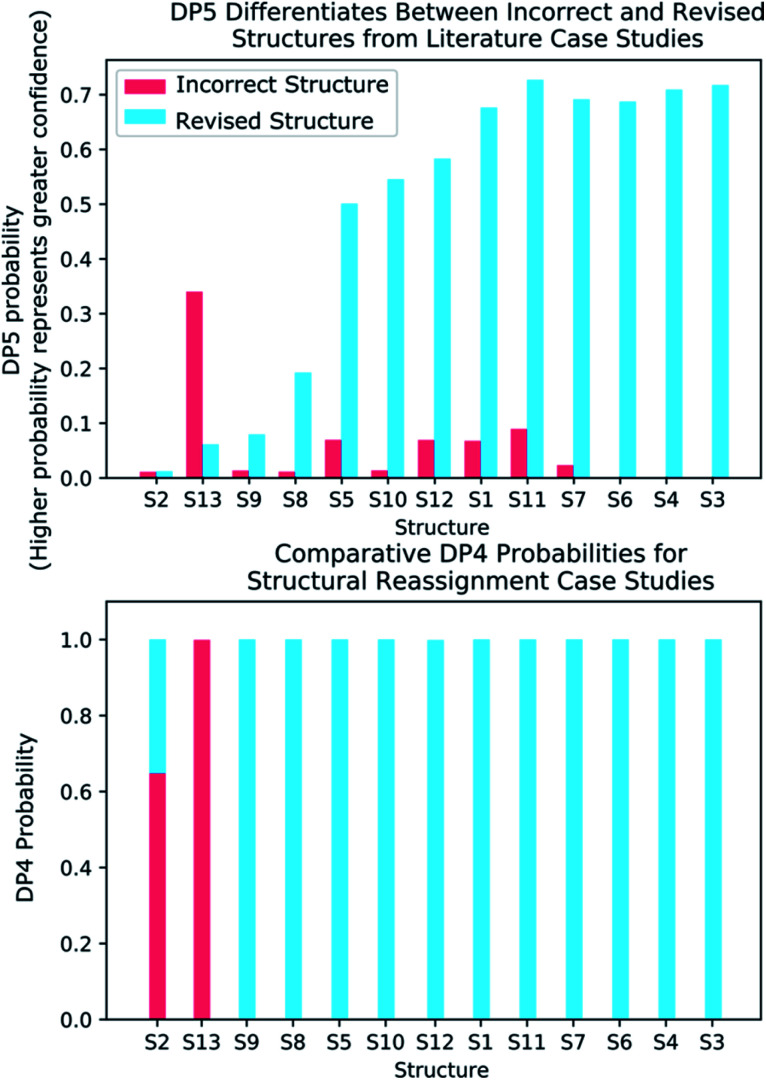
(Top) DP5 probabilities calculated for the thirteen incorrectly published structures and corresponding revised structures. DP5 assigns much greater confidence to the revised structures and also displays the three cases where both the initially proposed and revised structures are equally improbable. (Bottom) DP4 probabilities calculated for the same thirteen examples. These results show how the DP5 probability can be used to test the reliability of a DP4 calculation, as only DP5 can discern if any of the structure proposals are likely to be correct.

**Fig. 7:**
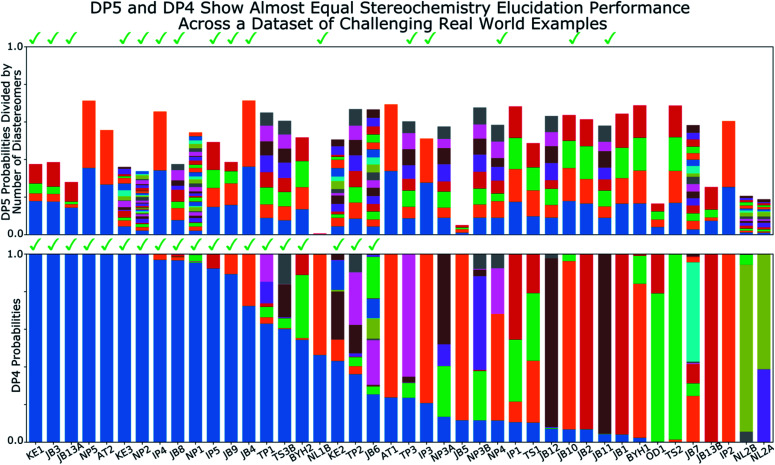
Results of DP5 (top) and DP4 (bottom) calculations on a dataset of 42 challenging real world stereochemistry elucidation examples (see ESI Section 5.1† for structures). In both plots probabilities calculated for each diastereomer are stacked in the same order with matching colours, the correct diastereomer is always represented by the blue bar at the bottom of the stack. The checkmarks above each plot indicate molecules correctly assigned by each program. The DP5 probabilities have been divided by the number of diastereomers for each molecule, this ensures the total sum of these probabilities is within the 0–1 range (see ESI Section 5.1† for unnormalized results). These results show the two systems display similar stereochemistry elucidation performance, DP4 assigns 19 molecules correctly, whilst DP5 assigns 16 correctly, the probabilities of assigning as many molecules in this dataset correctly by chance are ∼0.0001 and ∼0.01 respectively. Both DP5 and DP4 probabilities are based only on ^13^C NMR data.

The database of 5140 organic molecules from NMRShiftDB was used in comprehensive leave-one-out style cross validation study summarised in Fig. 4 (see ESI 3.2†). In this study DP5 analysis of correct and incorrect proposed candidate structures was simulated by permuting the experimental data between the structures in the dataset to form correct and incorrect pairs of structure and spectra. This analysis is particularly powerful as negative examples could be synthesised from real world data, avoiding more unreliable methods involving generating fake experimental or calculated spectra. This analysis was used to develop DP5 and was repeated for many different formulations of the DP5 probability (see ESI Section S4.1†). The results for the final DP5 system can be seen in Fig. 4. To ensure the computational feasibility of this analysis, only a single DFT optimised conformer was considered for each pair of structure and spectra. Full conformational analysis here would require significant additional computational resources, we assume that any subsequent decreases in accuracy in the DP5 probabilities here will affect both the correct and incorrect pairs equally, and hence will not change the final conclusions sufficiently to justify the substantial extra expense. In all other experiments structures were subject to full conformational analysis (as is standard in the final program). The final DP5 methodology was then evaluated against a series of thirteen real world structure reassignment problems from the literature, molecules S1a–S13b presented in Fig. 5. The results of this study are shown in Fig. 6. As the final and most subtle test, the DP5 probability was evaluated against the same dataset of 42 relative stereochemistry problems used to evaluate DP4-AI.^31^ With an average of 3.49 stereocentres per molecule and a diverse range of natural-product-like carbon skeletons, this dataset provides rigorous evaluation of DP5 for many real world applications. The results of this analysis are displayed in Fig. 7.

## Discussion

Results from the combinatorial analysis of the 5140 molecules from NMRShiftdb are presented in Fig. 4. In case 1, all incorrect pairs of structure and spectra with maximum errors <10 ppm are considered equally. This represents the situation where an experienced chemist should be able to accurately and reliably predict whether a chemically reasonable proposed structure is likely to be correct or incorrect based on the DFT-NMR prediction errors alone. There is very little overlap between the DP5 probability distributions for the correct and incorrect structure proposals, the modal DP5 probability for correct structures being the maximum possible value (see ESI Section S2.8†). The incorrect structures display the opposite pattern, with the modal value at close to zero. This result highlights the DP5 probability's ability to differentiate reliably between correct and incorrect structures using DFT prediction errors as well as an experienced chemist in situations where the incorrect and correct structures are both structurally dissimilar and produce very different spectra. When paired this way, the correct and incorrect pairs show different MAE distributions, with the incorrect pairs displaying a larger modal MAE and a greater variance. The DP5 probability would be even more useful if it could reliably differentiate incorrect structure proposals following the same MAE distributions as the correct structures. This is tested in Fig. 4, case 2. The incorrect pairs are assigned weights to ensure that they follow the same MAE distribution as the correct pairs (see ESI Section S3.2†). This gives an expectation number of about 5330 incorrect structure proposals with a mean error of <2 ppm. This mean DFT error is same as that of the correct structures, and hence the correct and incorrect structures now produce spectra that not only very similar to each other but are also often within DFT error of each other. This represents the radically more challenging situation where the correct and incorrect structure proposals have significantly different structures but are practically indistinguishable by their DFT prediction errors. In this situation an expert chemist would have significant difficulty deciding whether a proposed structure is correct or incorrect, and in some cases this may be impossible without collecting additional information. The results of this study are particularly exciting, as despite this test proving to be more demanding, the DP5 probability is still able to correctly differentiate many correct and incorrect structures. This is shown by the DP5 probability frequency distributions, with the correct pairs maintaining a strong peak at the maximum possible value and the incorrect pairs having significant density towards zero. This shows that DP5 will typically assign lower probabilities to incorrect structures even when they display similar spectra to the correct structure. It is able to do this because the internal statistical model takes into account the structure of the proposal, which is not possible in traditional error analysis.

A very interesting feature that these results illuminate is the value of the maximum possible DP5 probability. This value is dependent on many factors including, the dataset of atomic environments, the atomic representation chosen, and, most notably, the inherent uncertainty in the DFT NMR predictions. Using this state-of-the-art and highly-optimised set of conditions, DFT NMR predictions still have a MAE of 1.57 ppm. As a result, even if a proposed structure is correct, the DP5 probability has to take into account the possible variance in NMR predictions and reflect this uncertainty. Therefore, when using this set of DFT conditions, the user can never be more than 72% confident that a structure is correct using one dimensional DFT NMR predictions alone, this important functionality has previously been missing from other structure validation systems. This value acts as a metric for assessing the accuracy of DFT NMR calculations and the reliability of the DP5 calculations. We expect that the use of even larger databases and even higher levels of theory will raise this limit. Equivalently, this can be interpreted as acknowledging that an incorrect structure could possibly produce a set of errors equally or more convincing than the correct structure, just as two molecules may produce similar experimental spectra. However, this is seldom a problem in organic chemistry as in most real-world applications, there are additional constraints on the potential structures that need to be considered. However, one can sometimes be 100% confident that a structure is incorrect. For example, in robot-controlled syntheses, the particular sequence of reactions is known limiting the potential products. In most cases, a DP5 probability of 73%, combined with this additional data, will give the chemist much higher levels of certainty their structure is correct. In cases where multiple structures give high DP5 probabilities for the same spectra, this is a good indication of where DP4 can be applied in conjunction with DP5 to give even more accurate relative probabilities.

To further test the efficacy of DP5 analysis, the system was evaluated against thirteen real-world examples of structures originally incorrectly published in the literature and later reassigned, these structures are presented in Fig. 5.^1,58,61–65^ The results of this study are striking and are presented in Fig. 6. In all cases (except S13) the DP5 probabilities of the incorrect structures are equal to or close to zero. This illustrates that DP5 can reliably pick out structures that are likely to be incorrect, and will suggest to the user in these cases that the proposed structures may require revision. On the other hand, DP5 typically assigns probabilities close to the maximum to the correct structures. This will inform the user these structures are likely to be correct. There are three examples S2, S8 and S9 where the DP5 probabilities of the correct and incorrect structure are both low. This result is not surprising and does not show a weakness, but rather a distinct advantage of the DP5 probability over DP4. Being a single structure probability, DP5 is questioning the initial structure and the revision independently, if both structures are improbable DP5 can assign low probabilities to both. In these situations, when all the candidate structures are unlikely, DP4 probabilities must still sum to one and DP4 will typically randomly show overconfidence in one of the structures. This behaviour was clearly displayed when the analysis was repeated using DP4. The low DP5 probabilities for S8, and S9, suggest DP4 may have also assigned these structures correctly by chance. These results highlight the consequences of the underlying assumptions of the DP4 methodology. For DP4 probabilities to be reliable, the correct structure must be present in the list of candidates. When this is true, DP4 is often more accurate than DP5 as it is more sensitive to slight differences in NMR spectra and as more information is available within the calculation. This makes DP4 the perfect system when the correct structure is guaranteed to be in the list of proposals. However, in cases where none of the candidate structures may be correct, only the DP5 probability can reflect this and can be calculated to assess the reliability of the DP4 calculation.

By analysing the DFT-prediction errors observed for these examples (see ESI Section 7†), we can gain insight into how the DP5 probabilities have been assigned. In examples such as S3 and S4, the correct structures show significantly smaller errors than the incorrect structures. In these situations a trained chemist would be able to distinguish these structures using the DFT-NMR prediction errors. These results show DP5 also reliably assigns probabilities to these structures confirming the findings of the first analysis of the molecules from NMRShiftdb. In examples such as S5 and S8, both the correct and incorrect structures display a number of atoms with large errors. In these cases, analysis of the DFT errors may allow a chemist to qualitatively decide which structure they think is correct. However, previous work in this field has shown that large errors occur frequently in correct structures, and as a result statistical distributions with wide tails must be used to describe DFT-NMR prediction errors accurately.^12,55^ In addition, it is also very well known that the shapes of the DFT-NMR prediction error distributions vary in complex ways with the structure of the atomic environment.^55,66^ As a result, it is often difficult to draw accurate and reliable conclusions about structure proposals based on the sizes of the prediction errors alone. In contrast, DP5 quantitatively analyses the DFT-NMR prediction errors whilst taking into account how the underlying distribution will be affected by the structure of the atomic environment in question. This behaviour is clearly displayed in the atomic probabilities calculated for these examples, atoms with larger errors sometimes display lower probabilities than those with smaller errors, and *vice versa* (see ESI Section 7†). Finally, by utilising eqn (3) and (4) to combine the atomic probabilities, the DP5 probability reliably reflects the overall probability for the whole molecule in a way a simple error analysis cannot.

S2 is an interesting example and highlights another useful property of the DP5 probability. In the original interpretation of the NMR spectrum of S2, a peak was missed leading to a large difference between the experimental NMR spectrum and the DFT predicated spectrum for this molecule. DP4 is unable to detect this error and a probability of 100% to the incorrect molecule. DP5 on the other hand, when utilising this incorrect NMR assignment, gives very low confidences to both structures. This shows DP5 does not only detect errors in structures, but as it is calculating the probability a spectrum corresponds to a structure, it may also suggest whether an error has been made in the NMR interpretation. If the missing peak is included in the NMR analysis, the DP5 probability of the correct structure rises to 21.6% whilst the probability of the incorrect structure remains at 0%, again displaying the usefulness of DP5.

S13 is the only example assigned incorrectly by DP5 and should be considered in more detail. In this case the predicted spectrum of the correct structure is significantly more different to the observed spectrum than that of the incorrect structure. This is most likely due to the large quantity of Br atoms in the correct structure decreasing the accuracy of the DFT calculations. This emphasises that DP5 should strictly be interpreted as the probability of a collection of NMR-DFT prediction errors being observed for a given structure. In this case, given the DFT results, the incorrect structure is a better fit for the observed spectrum and thus is assigned a higher DP5 probability.

These results demonstrate some key behaviours of the DP5 probability and in addition confirm with real world examples the conclusions made during the analysis of the NMRShiftdb molecules. These results also suggest utilising DP5 analysis may have prevented these incorrectly assigned structures from being published.

To confirm the finding in the second stage of the analysis of the NMRShiftdb molecules that DP5 can reliably differentiate correct and incorrect structure proposals indistinguishable by their errors, DP5 was evaluated against a set of 42 real world relative stereochemistry elucidation problems originally used to test DP4-AI (see ESI Section 5.1†).^31^

Relative stereochemistry elucidation is a particular challenge as the average difference between spectra of diastereomers (∼1 to 2 ppm) is similar to the average error in state-of-the-art DFT calculations. As in the second stage of the analysis of the molecules from NMRShiftdb, this results in diastereomers being extremely challenging to distinguish even by an expert chemist using traditional analysis with the DFT errors alone. This test is significantly different to those already presented. In the previous examples, a single structure is proposed for a single spectra. In contrast, when resolving relative stereochemistry, multiple candidate structures (diastereomers) are proposed for the same spectra and the correct structure is assumed to be one of the candidates. This is the situation for which DP4 was designed; DP5 does not make this assumption. Despite this, Fig. 7 shows the performance of DP5 and DP4 is similar in this regime: DP4 assigns 19 molecules correctly whilst DP5 assigns 16 correctly, the probabilities of assigning as many molecules in this dataset correctly by chance are ∼0.0001 and ∼0.01 respectively, showing the significance of these results. Interestingly, whilst the number of molecules correctly assigned by the two systems is very similar, the behaviours of the two systems are distinct. DP5 typically assigns similar probabilities to all of the diastereomers, whilst DP4 often shows greater confidence in a smaller number. These behaviours clearly reflect the questions DP5 and DP4 have been designed to answer. DP5 is individually comparing each diastereomer to chemical space. DP5 assigns similar probabilities to diastereomers as they are typically more similar to each other than they are to other molecules across chemical space. This can be seen when comparing the MAEs between spectra of random molecules, ∼50 to 100 ppm with those seen for diastereomers, typically ∼1 to 2 ppm. In contrast, DP4 is directly comparing the diastereomers against each other, leading to a greater variation in the probabilities. This is often beneficial for DP4 as it always assumes the correct structure is in the list of proposals, whilst DP5 does not. These results again highlight the DP5 gives reliable overall probabilities without this assumption, while DP4 shows additional sensitivity based on this assumption. In the second stage of the analysis of the molecules from NMRShiftdb, DP5 was shown to typically assign much lower probabilities to incorrect structure proposals than to correct structures even when these displayed similar MAEs (∼2 ppm). In contrast, in the stereochemical examples, whilst DP5 again assigns a significant number of the molecules correctly, the calculated probabilities are more similar. The key observation here is that whilst differences between the spectra of the correct and incorrect structures are similarly small in these two situations, in the case of diastereomers, the structures of the proposals are also more similar. Therefore DP5 should be expected to assign similar probabilities to diastereomers. This illustrates a powerful behaviour of DP5, as the proposals become more similar to each other in structure, the probabilities DP5 assigns also become more similar. This behaviour is more useful than the alternative of assigning a probability of 1.0 to correct structure proposals and zero to everything else, as this allows the user to assess if their proposals are more or less similar to the true structure. This also clearly demonstrates that DP5 probability can be interpreted as a probability. If two structures are very similar and yield similar spectra, their probabilities of being correct when considering DFT-prediction errors only should necessarily be similar. In certain situations the reliability of the DP4 probability can be further increased, for example when the user has developed a custom statistical model for working with a particular class of molecules or when multiple sets of spectral data are available.

These results also demonstrate how DP4 and DP5 can and should be used together in cases where the user knows their proposal has multiple stereocentres. In examples: JB5, OD1, NL1B, NL2A and NL2B, where the DP5 probabilities for all the diastereomers are low (the total height of each bar in DP5 results in Fig. 7 can be interpreted as the average confidence the diastereomers) this suggests that the DP4 probabilities are likely to be less reliable. There are a number of reasons why the DP5 probability may be low in this way, for example, the correct structure may be missing from the list, there may be a problem with the NMR assignment or there may be an impurity in the spectrum. The results of this test are very exciting and the application of the DP5 probability in situations with multiple structure proposals is now being more thoroughly explored.

These examples show how DP5 can serve as a valuable tool whenever a new molecule is made, increasing confidence when proposed structures are correct, highlighting cases where they are not and also playing a stern jury when an improbable but correct structure has been proposed.

## Conclusions

In conclusion, we have developed a new measure to quantify molecular structural uncertainty, the DP5 probability. This work represents a leap forward in quantification of structural uncertainty as instead of a comparative dimensionless parameter, DP5 quantifies the probability of a structure being correct. This system was rigorously evaluated by a cross validation study and it was found that DP5 could perform as well as a human in classifying correct and incorrect structure proposals and in some cases could classify structures indistinguishable to a chemist. DP5 was evaluated against thirteen real-world examples of structures that were incorrectly published and subsequently revised in the literature. In all these challenging cases, DP5 expressed the maximum concern for the incorrect structures and was on average 41% more confident in the revised structures. DP5 was finally evaluated against 42 real world stereochemistry elucidation examples, displaying almost equal performance to DP4. The DP5 probability can be calculated fully automatically and so should find wide applications in uses cases such as high throughput reaction screening, automated chemical synthesis and drug discovery. In addition, DP5 may be run on a single molecule basis and the results explored utilizing the GUI, helping to guide the development of complex syntheses. This work also suggests how DP5 may be developed to help further to accelerate chemical discovery. The DP5 probability has been evaluated here with ^13^C NMR data, DP5 is currently being extend to utilise different types of spectral data, and a DFT free version of DP5 is also being explored. In addition, utilizing DP5 alongside generative models and other machine learning methods to automatically guide structure determination is an attractive possibility. The DP5 system is available as open-source software at https://github.com/Goodman-lab/DP5.

## Data availability

Results of all DFT and MM calculations are available from https://doi.org/10.17863/CAM.81332.

## Author contributions

Prof J. M. Goodman conceptualised and supervised the project. A. Howarth, performed the research, and also built and tested the software described in the paper. A. Howarth wrote the paper with the guidance of Prof J. M. Goodman.

## Conflicts of interest

There are no conflicts to declare.

## Supplementary Material

SC-013-D1SC04406K-s001
