# Supplementary material for: The DP5 probability, quantification and visualisation of structural uncertainty in single molecules
Source: Chem Sci. 2022 Feb 25;13(12):3507–18. doi: 10.1039/d1sc04406k (PMC8943899; doi:10.1039/d1sc04406k)
Supplement: SC-013-D1SC04406K-s001 [file SC-013-D1SC04406K-s001.pdf]

Electronic Supplementary Material (ESI) for Chemical Science.
This journal is © The Royal Society of Chemistry 2022

The DP5 Probability, Quantification and Visualisation of Molecular Structural Uncertainty in Single Molecules

Supporting Information

Alexander Howarth, Jonathan M. Goodman*

Centre for Molecular Informatics, Department of Chemistry, University of Cambridge, Lensfield Road, Cambridge CB2 1EW E-mail: jmg11@cam.ac.uk Phone: 01223 336434

Contents

1 Utilising DP5 **1**

2 Program Description **4**
2.1 NMR Shift calculation 4
2.2 Bespoke Atomic Prediction Error Probability Function Generation 5
2.3 Atomic Representation Generation 6
2.4 Parameter selection in Gaussian Kernel and FCHL representation generation 6
2.5 Atomic Prediction Error Probability Function Integration 8
2.6 Atomic Prediction Error Probabilities from Multiple Conformers 10
2.7 Molecular Probability From Atomic Probabilities 10
2.8 Bayesian Molecular DP5 probability 11

3 Program Evaluation **11**
3.1 External Dataset 11
3.2 Combinatorial Study 11

4 Kernel Ridge Regression **12**

5 Results **18**
5.1 Stereochemistry Elucidation Results 18
5.2 Combinatorial Study Results 18

6 NMRShiftDB Molecules **57**

7 Reassignment Examples Shift Data **64**
7.1 S1 65
7.2 S2 66
7.3 S3 67
7.4 S4 68
7.5 S5 69
7.6 S6 70
7.7 S7 71
7.8 S8 72
7.9 S9 73
7.10 S10 74
7.11 S11 75
7.12 S12 76
7.13 S13 77

1 Utilising DP5

DP5 can be downloaded from https://github.com/Goodman-lab/

DP5 has been developed for fully automated structure elucidation. DP5 can be easily run standalone utilising the command line interface, or integrated into a users workflow using the source code. A single command is required to run DP5 for a compound or multiple compounds. This command indicates the calculations the user wishes to perform (i.e DFT geometry optimisation, higher level DFT single point energy calculations and NMR calculations) and the desired computational conditions (default conditions will be used if none are provided). Once this command has been entered, DP5 will manage all of the required calculations to yield probability values with no further intervention from the user being required.

DP5 maybe run locally on a Desktop PC and also provides support for utilising external clusters.

DP5 can also be run through the more familiar GUI interface, Figures 1 -4. The GUI allows the user to explore many features of a DP5 calculation, such as, the number of conformers found during the conformational search, their energies and the DP5 probabilities assigned to each test structure. In addition, the individual atom prediction error probabilities can be visualised, this may help users understand why a

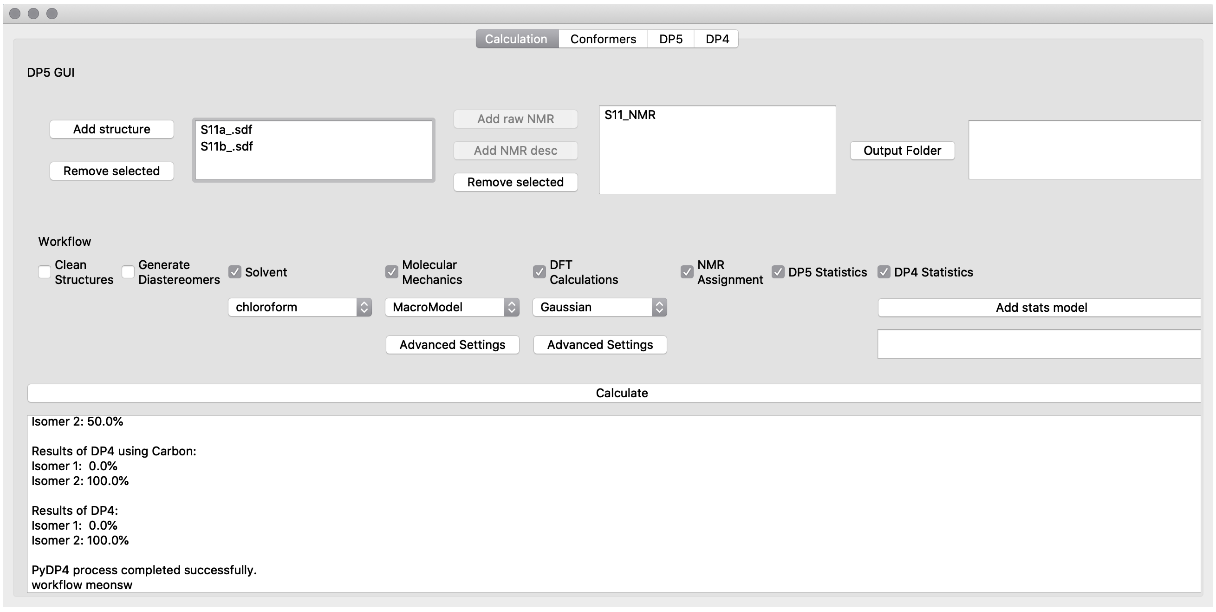

Figure 1: The DP5 GUI can utilised to set up and run DP5 calculations

particular DP5 probability has been calculated and suggest potential regions of the structure that are likely to be correct and incorrect.

Our automatic NMR processing software, NMR-AI has also been integrated into DP5, as a result, DP5 probabilities can be calculated from raw NMR data. By utilising the GUI, users can also explore the NMR assignments made by NMR-AI and thus further understand how a DP5 probability has been calculated.

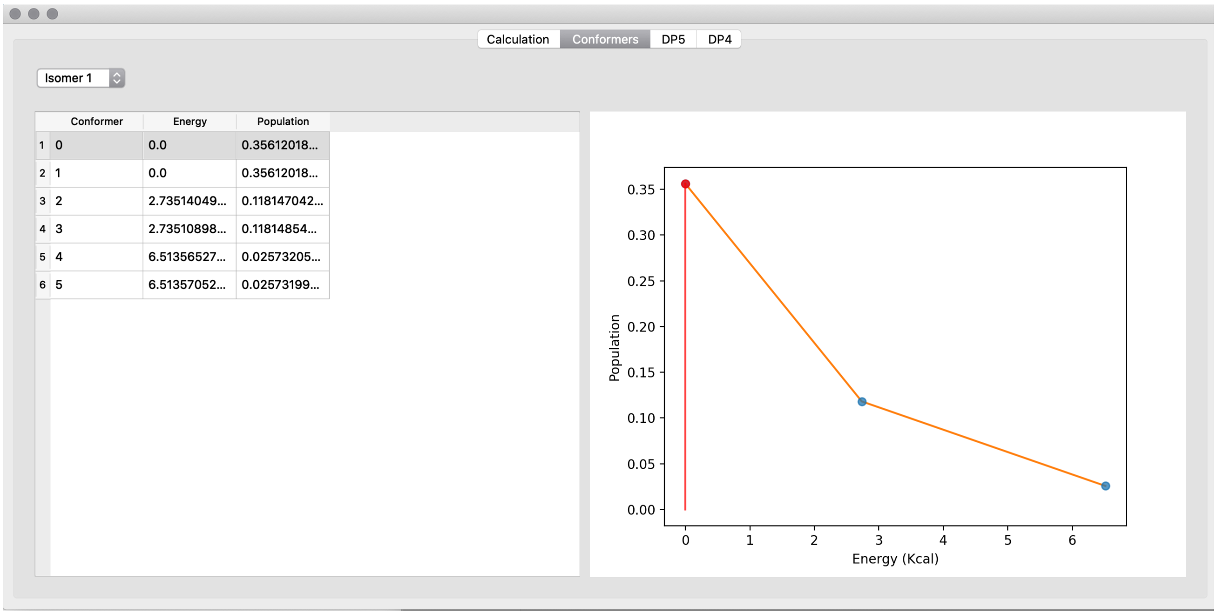

Figure 2: The DP5 GUI can utilised to investigate conformer energy distributions for each proposed structure

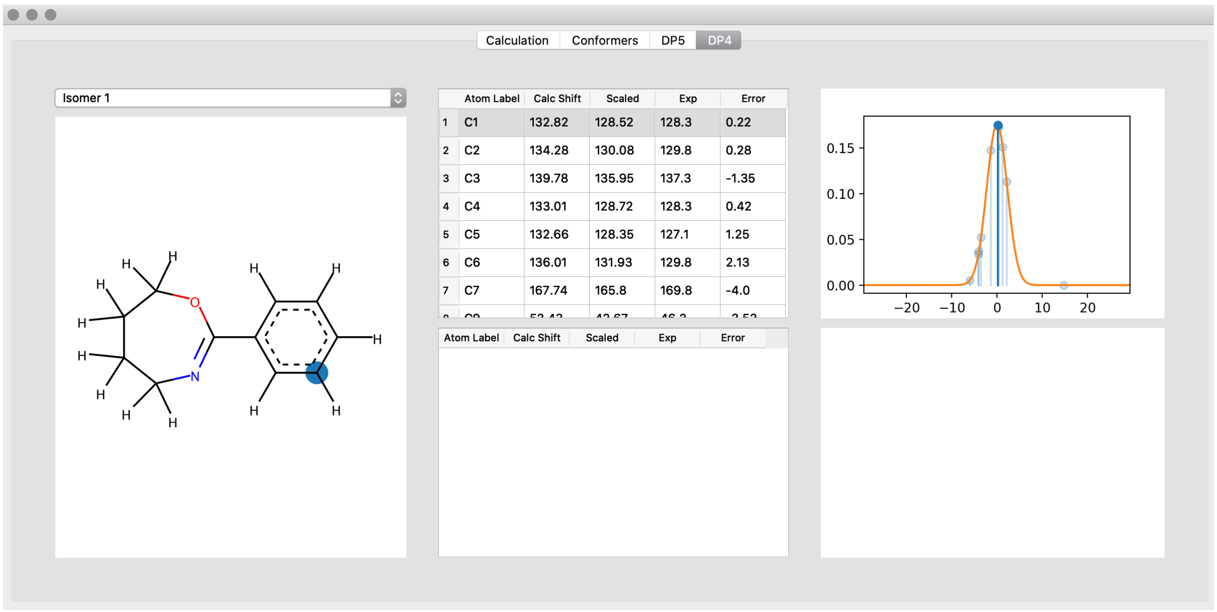

Figure 3: The DP5 GUI can utilised to investigate DP4 statistics for each proposed structure

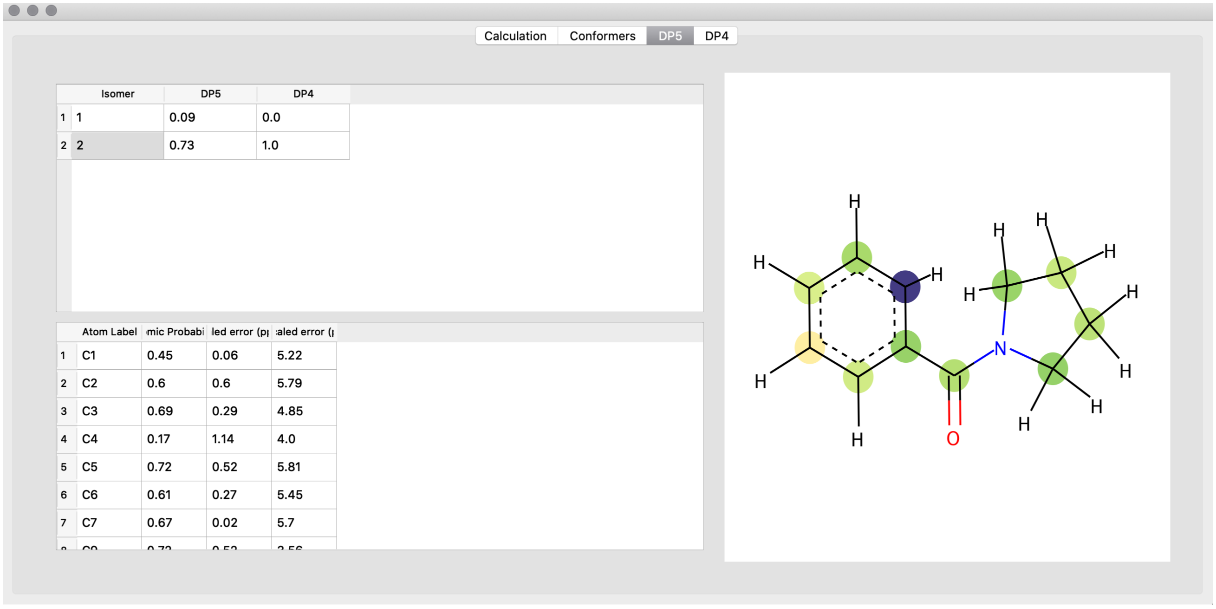

Figure 4: The DP5 GUI can utilised to investigate DP5 statistics for each proposed structure and also interactively displays atomic probabilities overlaid onto the proposed molecular structure

2 Program Description

Figure 5. displays a roadmap of the DP5 probability calculation. The basic premiss of the calculation is as follows. For each atom in the candidate structure a chemical shift is calculated using DFT. By subtracting this value from the experimentally observed shift, a prediction error can be calculated for that atom. Each atom is treated as a random variable that can take one of two states, correct or incorrect. Using a statistical model, the probability of observing a prediction error of this size can be found. This probability is equated to the probability of the atom in the candidate structure being incorrect. The atomic probabilities are then combined to yield an overall molecular probability. The DP5 probability is then calculated from this value using a Bayesian correction function. Each step in this calculation is described in more detail in the following sections.

2.1 NMR Shift calculation

DP5 calculates NMR shifts for the atoms in the candidate structure utilising the highly optimised and well established method developed in previous works.[1–4] First the a molecular mechanics search is performed to obtain a representative set of conformer geometries, all conformational searches are performed in the gas phase utilising the MMFF force field and a mixture of Low Mode following and Monte Carlo search algorithms. The step count for MacroModel[5] is set so that all low energy conformers were found at least 5 times.

The conformer geometries are then optimised at the DFT level of theory (B3LYP/6-311g(d)).[6,7] DP5 includes quantum mechanical calculation support for both the commercial program Gaussian[8] and the free software Tinker.[9]

For each conformer a single point energy calculation is completed (M062x/def2-TZVP)[10–12] and NMR shielding constants are found using the GIAO method.[13] The functional mPW1PW91[14] and 6-311G(d) basis set was chosen for NMR shift prediction as this has been shown to be optimal for DP4 calculations.

All calculations are managed by the DP5 Python script written in Python 3.7. DP5 is available from https://github.com/orgs/Goodman-lab/

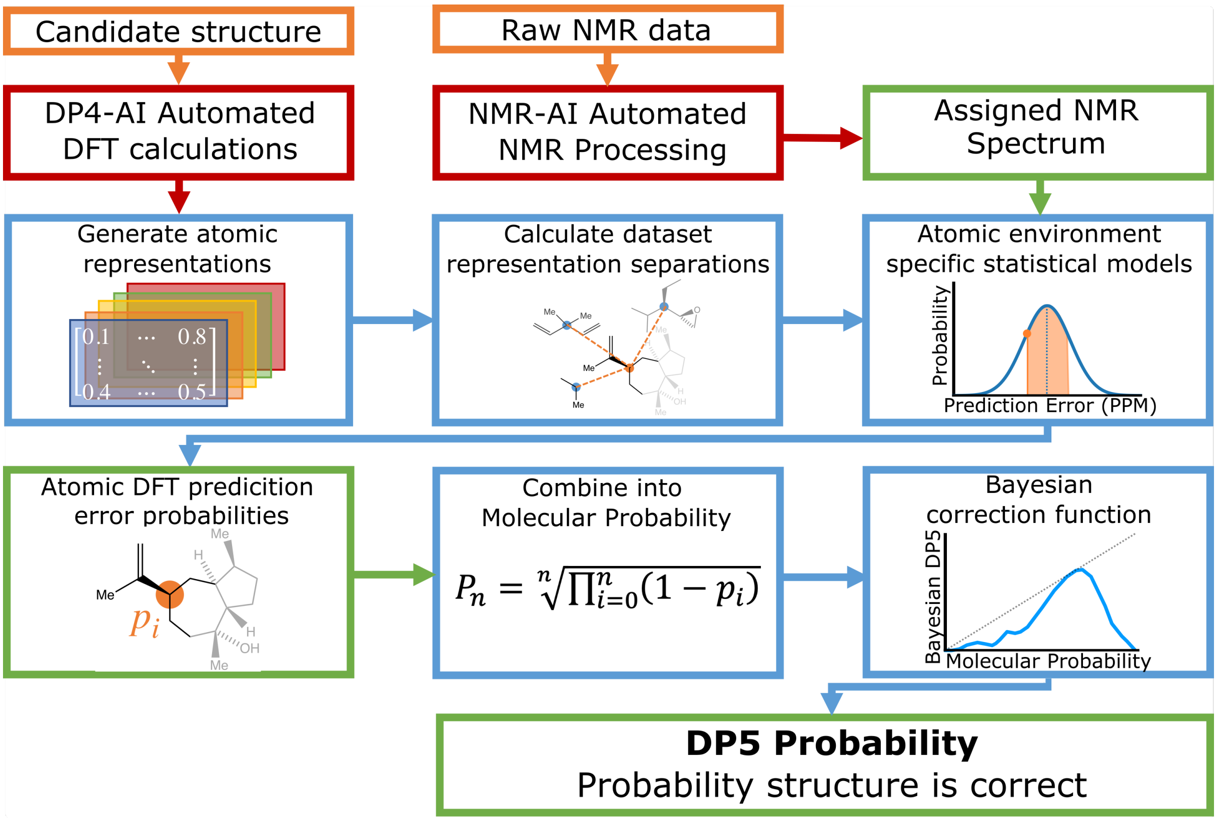

Figure 5: Schematic diagram of how a DP5 probability is calculated by the DP5 program. Each stage of this calculation is described in the text below.

2.2 Bespoke Atomic Prediction Error Probability Function Generation

The backbone of the DP5 probability calculation is generating and integrating a bespoke DFT-NMR prediction error probability function for each atom in every conformer of the candidate structure.

It is well known that the expected magnitude and variance of DFT prediction errors for different functionals show strong complex, nonlinear dependencies on atomic environment.[4,15] A number of different statistical models have been explored in previous works to help alleviate this issue such as, multi-gaussian, multi-region and kernel density estimation (KDE) models.[4] However, these models all display the same type of issue. The underlying assumption here is that the probability of a prediction error of a specific magnitude being observed in an external dataset is equal to the probability of observing the same prediction error within a given structure, this is not always the case. For example, some atomic environments may be expected to produce large prediction errors, these types of environments are typically underrepresented in the datasets used to develop the prediction error statistical models, as a result the corresponding prediction error probabilities will be close to zero. This is not a particular problem for a DP4 calculation, due to its comparative nature, these same systematic errors are likely to be present in all the structures being compared and tend to cancel out. However, this is a significant problem when developing a standalone probability for a single candidate structure. To solve this issue, DP5 produces a bespoke prediction error probability distribution for every atomic environment in every conformer of the candidate structure. This probability distribution takes into account the molecular geometry around each atom and the surrounding atom types by utilising the FCHL representation.[16]

The bespoke prediction error probability distribution for a given atom in a given conformer of the candidate structure is found as follows. The FCHL representation for the atom is calculated (this calculation is performed utilising the python package qml). This calculation has similarly been performed for 63542 carbon atoms present in a dataset of 5140 molecules from NMRShiftDB.[17,18] The l2 distance between this representation and all those in the dataset are then calculated (also performed using qml). These distances are then mapped onto a similarity value using a gaussian kernel, equation 1. This yields a similarity between the test atomic environment and those in the dataset. A kernel density estimation is then performed on the prediction errors from the atoms 63542 in the external dataset, with each

$$\text{Similarity}_{\text{ij}} = \exp\left(-\frac{||A_i - A_j||_2^2}{2\sigma^2}\right) \tag{1}$$

equation for the similarity between two atomic environments utilising a gaussian kernel, where $||A_i - A_j||_2$ describes the l2 distance between the FCHL [16] representation for atomic environment i and atomic environment j and σ is an optimised parameter (see section 2.4)

point weighted by the corresponding atomic environments similarity to the test atoms environment. Using this methodology a bespoke prediction error distribution is produced for the test atom taking into account the chemical environment the atom is in. Systems were also tested with weights multiplied by the corresponding regression coefficients (see section 4) found by solving KRR models for DFT NMR error prediction.

In order to simplify the integration process the external error dataset is made symmetrical around 0, this is achieved by taking the absolute value of each error in the dataset followed by concatenating these values and the same values multiplied by -1 into the same dataset (with twice the size) the previously calculated weights are used for both the positive and negative error values.

2.3 Atomic Representation Generation

The FCHL representation [16] was chosen due to the similarity between this calculation and kernel ridge regression (KRR). The FCHL representation of atomic environments have been used very successfully in KRR models to predict chemical shifts. [19] Similar KRR models were also constructed and evaluated in this study, see section 4. This illustrates that the FCHL representation effectively encodes the information required to define the properties of an atomic environment and that this encoding can be used to calculate the similarity between different atomic environments. As this is exactly the property required for this work and due to the ease of use, the FCHL representation was implemented in the DP5 calculation. A number of other representations were tested in this work including coulomb matrices and Morgan fingerprints of circular fragments around each atom, however, FCHL was found to be the best performing representation. It may be possible to improve the performance of DP5 by testing more atomic representations and potentially by generating a bespoke representation for this task.

In order to calculate the covariance of two FCHL representations, they must have the same vector length. This places an upper limit on the number of atoms in a molecule that the model can compare. In this study this value is set to 83, the maximum number of atoms in the NMRShiftDB dataset. For any molecules with more than 83 atoms DP5 produces molecular fragments with radius 3 around each atom in the molecule, these fragments must have fewer than 53 atoms (assuming a maximum valence of four) and a separate model based on representations of this size is used instead. No significant loss in accuracy was seen when using this fragmentation method for KRR NMR shift prediction tasks, demonstrating this is a reasonable approximation for larger molecules. This is likely to be due to the radial cutoff incorporated into the FCHL representation.

2.4 Parameter selection in Gaussian Kernel and FCHL representation generation

In order to generate an FCHL [16] representation a radial cutoff value must be defined, in this work this was set to 4.53Å. This value was found to be the optimum value for kernel ridge regression models for NMR shifts prediction using the FCHL representation (see section 4). The optimum cutoff value of 4.53Å was found using bayesian optimisation by training KRR models on a smaller dataset of 500 molecules [19] and using the mean absolution prediction error of the model across the dataset as the loss function. Due to the similarity between the use of the FCHL representation in these KRR models and in the DP5 calculation, it was concluded that this cutoff value would be similarly applicable in this context.

The second important parameter in the DP5 calculation is the sigma (σ) value used in the gaussian kernel (equation 1) during the similarity calculation described in section 2.2. This parameter changes the distance in chemical space (FCHL representation space) over which two atomic environments will be described as similar. Similarly to the radial cutoff value, this parameter was also investigated when

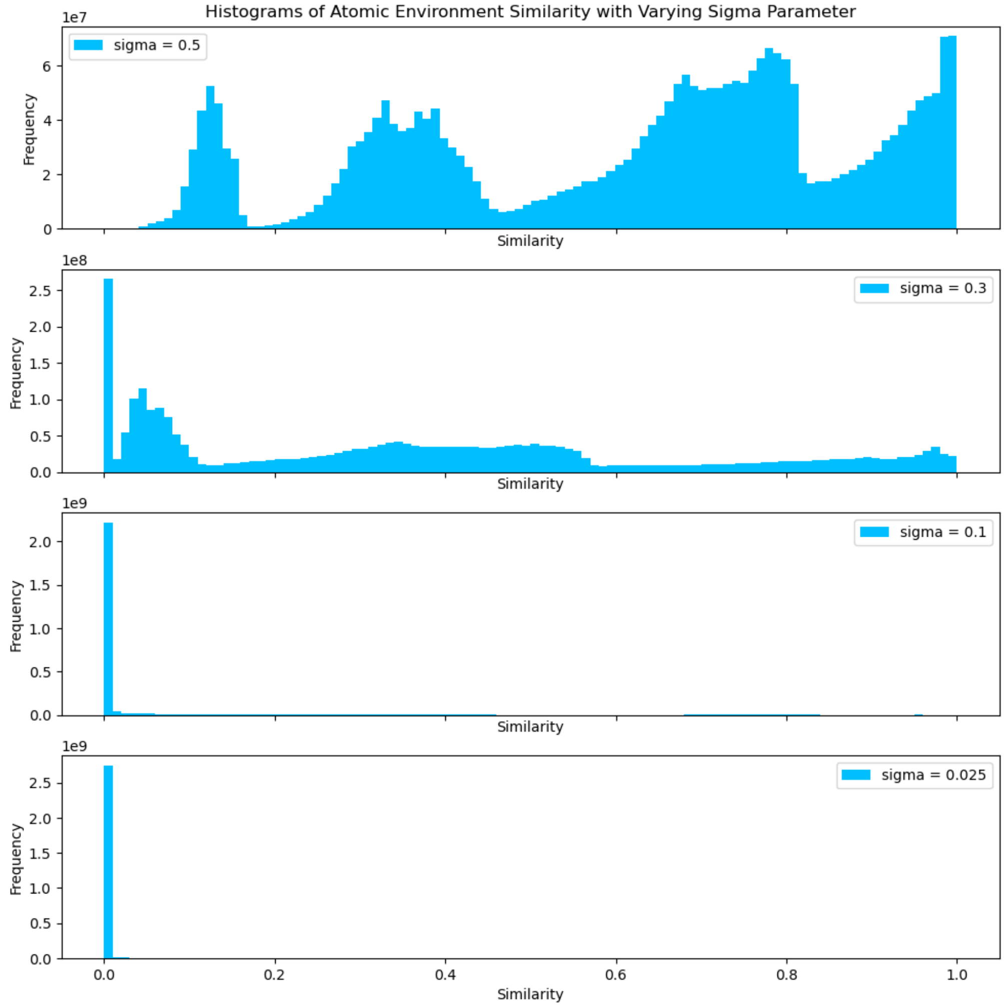

Figure 6: Figure illustrating the changes in frequency of atomic environment similarity with sigma parameter.

building KRR regression models for NMR shift prediction and optimised using bayesian optimisation. Due to the sensitivity of the DP5 probability calculation to changes in this parameter, the full DP5 system was later evaluated for multiple sigma values.

Histograms of atomic similarities between the atomic environments in the 5140 NMRShiftDB molecules were also plotted for multiple values of σ, the results of this study are presented in Figure 7. These histograms illustrate as the value of σ is decreased the average similarity between atomic environments in this dataset decreases. If σ is set at a large value, all the atomic environments become very similar to each other, in this limit the DP5 probability recovers the issue where prediction error probabilities are no longer dependent on the atomic environment they occur in. Whilst if σ is set too low, all environments will share no similarity, making it impossible to perform a weighted kernel density estimation as the resulting weights cannot be normalised. A number σ values were investigated during the main evaluation of the system as described in Section 3.2. Allowing σ value to vary with atomic environment was also investigated, this methodology is essentially the same as k-nearest-neighbours, which was also tested separately for different values of k. Utilising the k nearest neighbours method ensures each atomic environment has the same number of data-points contributing to its corresponding error kernel density estimation. This is also discussed in Section 3.2.

$$P_i = \int_{-1*\mathrm{error_i}}^{\mathrm{error_i}} pdf_i(x)dx \quad (2)$$

$$P_i = \int_{\mathrm{MAE}-|\mathrm{MAE}-\mathrm{error_i}|}^{\mathrm{MAE}+|\mathrm{MAE}-\mathrm{error_i}|} pdf_i(x)dx \quad (3)$$

where MAE is the mean absolute error in the external database

$$Pi = \int_{\mathrm{MAE_w}-|\mathrm{MAE_w}-\mathrm{error_i}|}^{\mathrm{MAE_w}+|\mathrm{MAE_w}-\mathrm{error_i}|} pdf_i(x)dx \quad (4)$$

where $\mathrm{MAE_w}$ is the weighted mean absolute error in the external database, the weights used for each datapoint are the similarity values calculated in section 2.2

2.5 Atomic Prediction Error Probability Function Integration

Once the prediction error probability distribution has been generated for an atom in a conformer of the candidate structure, this function must then be integrated to produce a prediction error probability for that atom. Three different sets of integration limits were tested in this work, they are shown in equations. 2,3 and 4. All three integrals represent reasonable definitions of the required probability, this choice was found to have a relatively large impact on the results of the DP5 calculation.

Initially equation 2 was investigated. Integrating with these limits means that atoms with large prediction errors will be assigned values close to one, whilst atoms with smaller errors will be assigned smaller probabilities. This method has the advantage that if by chance the DFT calculated shifts are very accurate this will be reflected in the atoms probability value. However, this method overlooks the fact that DFT NMR predictions have inherent error. The MAE for the DFT calculations run on the NMRShiftDB dataset is 1.57ppm, this implies that observing errors smaller than this value can be just as unlikely as observing large errors. Moreover, if an atom has a very small associated prediction error, it is perhaps more probable that the atom is incorrect than it is that the DFT calculation is very accurate by chance. Equations 3 and 4 take this into account, penalising errors either side of the dataset MAE value equally.

Equation 4 is the only method tested where the integration limits also depend on the atomic environment. In this approach, in the same way that points in the DFT-NMR prediction error KDE are weighted by their environments similarity to the test environment, MAE_w in equation. 4 is found by performing a weighted average. This method accounts for the fact that the mean error is also likely to change depending on the region of the representation space the test atomic environment is in. The differences between these integration limits are highlighted by Figure 7.

It was found that equations 3 and 4 were more effective than 2. Equation 2 typically assigns lower probabilities to atoms. This is because most atoms will have errors around the mean of the dataset, equation 2 will thus typically assign probabilities of 0.5 to these atoms. Equations 3 and 4 treat atoms differently, atoms with errors closer to the mean of the dataset (or closer to the mean observed in the corresponding region of chemical space in the case of equation 4) will be assigned probabilities closer to zero. This has the benefit that atoms with errors in the expected range will be assigned high confidence. Equation 3 and 4 do however show the disadvantage that if the DFT calculation predicts a shift with a lower error than expected, this prediction, similarly to one with a larger than expected error, will be assigned a low confidence. Equation 4 mitigates this issue as much as possible by allowing the mean value to vary with the region of chemical space the test atomic environment is in, making it much less likely for this situation to arise, as in environments where the DFT predictions are likely to be most accurate, the MAE_w in the integration limits will be close to zero.

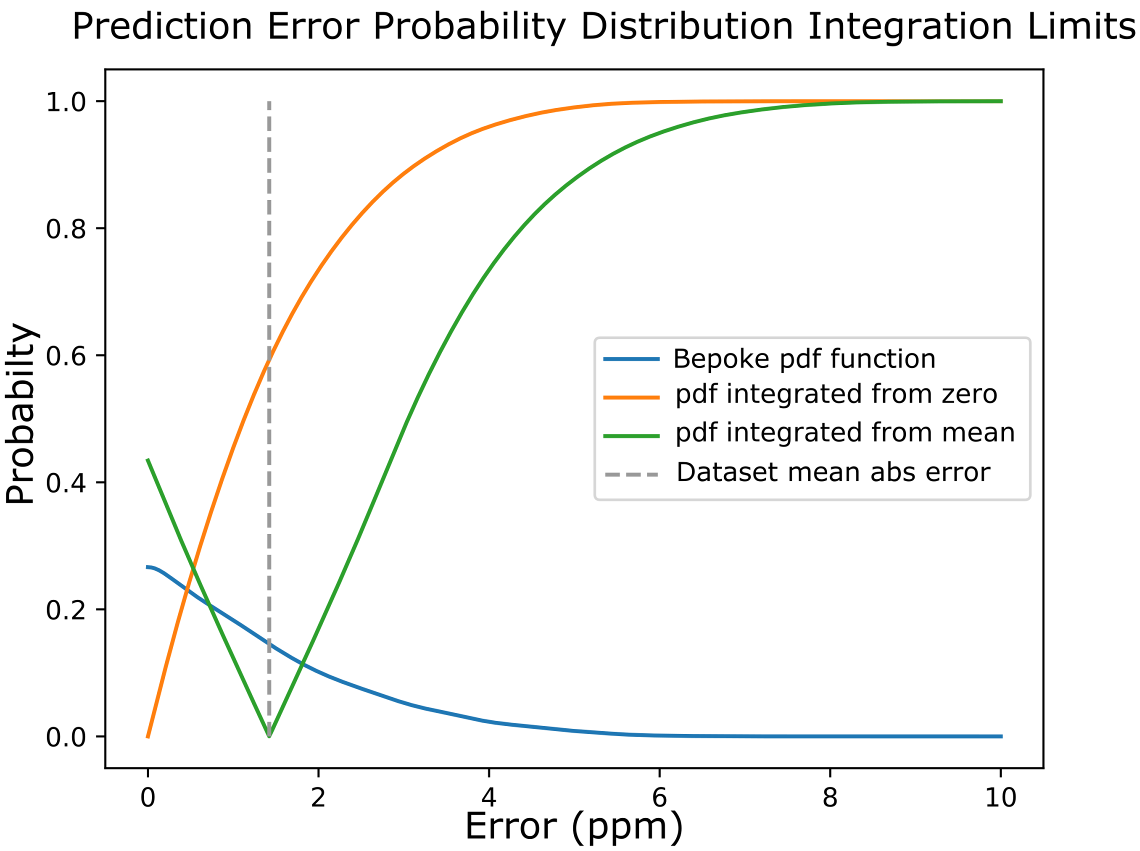

Figure 7: Figure illustrating effect of changing integration limits on atomic prediction error probabilities

$$P_{\text{DP5}} = 1 - \prod_{i=1}^{i=n} p_i \tag{5}$$

where i is the atom index and n is the total number of carbon atoms in the molecule

$$P_{\text{DP5}} = \prod_{i=1}^{i=n} (1 - p_i) \tag{6}$$

where i is the atom index and n is the total number of carbon atoms in the molecule

$$P_{\text{DP5}} = 1 - (\prod_{i=1}^{i=n} p_i)^{1/n} \tag{7}$$

where i is the atom index and n is the total number of carbon atoms in the molecule

$$P_{\text{DP5}} = (\prod_{i=1}^{i=n} 1 - p_i)^{1/n} \tag{8}$$

where i is the atom index and n is the total number of carbon atoms in the molecule

2.6 Atomic Prediction Error Probabilities from Multiple Conformers

To make the DP5 calculation as accurate as possible, probabilities for each atom are calculated not just for a single geometry, but for every atom in every conformer of the structure found during the conformational search. This is necessary as the probability assigned to each atom depends on the exact geometry of the atomic environment and this will change between conformers.

Once a DFT prediction error probability has been calculated for each atom in each conformer, these values are combined in a Boltzmann weighting process similar to the NMR shift calculation, to produce overall probabilities for each atom in the molecule. This Boltzmann weighting process utilises the same single point energy for each conformer found during the NMR Shift calculation stage (see section 2.1).

2.7 Molecular Probability From Atomic Probabilities

Having found the prediction error probabilities for each atom in the molecule, these must now be combined in order to produce a probability for the whole structure. This part of the DP5 calculation was explored extensively, and was found to have a large effect on the final results. A number of different methods for combining the atomic probabilities were tested, the most relevant of these are displayed in equations 5 to 8.

Equations 5 to 8 all combine the atomic probabilities in mathematically reasonable ways. Consider each atom as an independent random variable, each atom can have two states, correct and incorrect. The probability of the atom being in the incorrect is described by the atomic probability p_i. If these probabilities are multiplied together as in equation 5, the resulting value describes the probability of at least on of the atoms being correct. If the atomic probabilities are combined by equation 6, the resulting value instead describes the probability that all of the atoms in the structure are correct. In order to decide upon the most effective formulation of the DP5 probability, the most useful definition of when a structure should be classed as correct must first be decided upon.

It was found that when combining atomic probabilities by equation 5 the resulting molecular probabilities were often clustered around zero. This is due to a property of the underlying prediction error distribution,

these distributions are typically sharply peaked with very wide tails. As a result even in incorrect structures it is not uncommon to have number of atoms with large prediction errors, in these cases, when combining probabilities by equation 5 the molecular probability will be forced close to zero by these atoms. Incorrect structures are often highlighted by a small number of large errors, this leads to equation 6 seeming like a more useful choice. equation 6 performs much better than equation 5 however it still displays a tendency to force molecular probabilities to zero. The molecular probability should reflect the real world probability of a structure being correct rather than acting as a binary classifier of correct and incorrect structures. To help alleviate this forcing behaviour, equations, 5 and 6 were modified into equations, 7 and 8 by incorporating a geometric mean. The geometric mean was found to greatly reduce the forcing behaviour displayed by equations 5 and 6, giving a more balanced estimate of the structure being correct based upon all of the atoms. Equations including an arithmetic mean and median were also tested, however, these did not show any greater performance.

2.8 Bayesian Molecular DP5 probability

The final stage in the calculation applies Bayes theorem to the molecular probability to yield the over all DP5 probability. The purpose of this stage is to ensure the final DP5 probability assigned to a molecular structure is as close as possible to the real world probability as possible given the data available. In turn, this ensures that the DP5 probability is both easy to interpret and as useful as possible.

First a probability density function for the molecular probabilities assigned to the 5140 molecules in the dataset is found using a KDE. Similarly a weighted KDE is performed on the incorrect combinations of structures and spectra as described in section 3.2. Using these PDF functions the probabilities of the structure being correct and incorrect given the assigned molecular probability can be calculated. Finally using the information that a proposed structure must be either correct or incorrect we can define the Bayesian DP5 probability using equation 9. In the ideal case, this function (defined between zero and one) would fall exactly on the line y=x, meaning the assigned DP5 probabilities match the real world probabilities exactly. In order to ensure the final probabilities fall on the y=x line the DP5 probability calculated for the molecule is then scalded by equation 9.

$$\text{Bayesian DP5} = \frac{P(\text{correct}|\text{DP5})}{P(\text{incorrect}|\text{DP5}) + P(\text{correct}|\text{DP5})} \tag{9}$$

3 Program Evaluation

3.1 External Dataset

3.2 Combinatorial Study

A major challenge in the development of DP5 involved constructing a method to assess the efficacy of the system. As the DP5 probability is not a tangible physical property that can be measured, it is not straight forward to compare the DP5 probability assigned to a molecule with an experimental value. In order to solve this problem, a comprehensive cross validation methodology was developed.

This study was performed utilising the database of 5140 molecules from NMRShiftDB.[17,18] The NMR-ShiftDB ID for each molecule used is given in section 6. For each of these molecules, this database contains, a DFT optimised geometry, a DFT predicted carbon NMR spectrum and accompanying experimental NMR spectrum. Structure proposals are simulated by all forming pairs (or combinations) of experimental spectra and structures (with the same number of carbon atoms) in the dataset. This produces 5140 N combinations where a structure is paired with the correct experimental spectrum (correct combinations), and on the order of $\tilde{N}^2 - N$ combinations where an incorrect structure has been proposed (incorrect combinations).

In the first study, DP5 probabilities for all of the correct combinations are calculated. DP5 probabilities for incorrect combinations are calculated if the maximum error between the experimental spectrum and the paired DFT prediction spectrum is less than 10 ppm. This simulates correct and incorrect structure proposals that experienced chemists should be able to distinguish based upon the prediction errors alone.

It should be noted that the DP5 calculation relies upon the representations of the atomic environments in the same dataset of molecules. In order to make this study as robust as possible, all DP5 calculations are performed in a leave-one-out cross validation style where the representations for the atomic environments from the test structure are removed from the set used by DP5. The distributions of the resulting DP5 probabilities for the correct and incorrect combinations are plotted utilising a kernel density estimation. All the results of this study are presented in section 5.

If all incorrect combinations with a maximum error less than 10ppm are considered, the mean absolute error distribution (MAE) for the correct and incorrect combinations are expected to be different. Typically, the incorrect combinations have larger MAEs than the correct combinations. The DP5 probability would prove even more effective if it could be used to distinguish between correct and incorrect structure proposals that belong to the same MAE distribution. In the second stage of this evaluation the methodology is modified to account for this. The MAE for each incorrect combination is calculated, the probability of a correct combination having this MAE is found using the corresponding empirical PDF function. This probability is assigned to the incorrect combination as a weight, all subsequent frequency plots are performed using weighted kernel density estimations, this process is equivalent to directed sampling of the incorrect combinations. The effect of weighting the incorrect combinations in this way is the MAE distribution of the weighted incorrect combinations now approximates (as closely as possible) the MAE distribution of the correct combinations. As a result when these weights are considered the resulting plots and statistics simulate the case where both the correct and incorrect combinations belong to the same MAE distribution. Another effect of this modification is there is no longer an integer number of incorrect combinations, but rather, the sum of the weights assigned to the incorrect combinations gives an equivalent expected number of incorrect combinations. The results of this study evaluate the performance of the DP5 probability in the limit where the correct and incorrect combinations are indistinguishable by their prediction errors to even an experienced chemist. All the results of this study are presented in section 5.

This combination style analysis is particularly powerful as negative examples of incorrect structure proposals could be synthesised from real world data, avoiding more unreliable methods involving generating fake experimental or calculated spectra.

4 Kernel Ridge Regression

The KRR model for some property y for some system with the vector representation $\tilde{\boldsymbol{A}}$ is defined by equation 10

$$y(\tilde{\boldsymbol{A}}) = \sum_i \alpha_i K(\tilde{\boldsymbol{A}}, \boldsymbol{A}_i) \tag{10}$$

The kernel matrix K is found by equation 11, where $\boldsymbol{A}_i$ is the vector representation of datapoint i

$$\mathrm{K_{ij}} = K(\boldsymbol{A}_i, \boldsymbol{A}_j) = \exp\left(-\frac{||\boldsymbol{A}_i - \boldsymbol{A}_j||_2^2}{2\sigma^2}\right) \tag{11}$$

The regression coefficients $\boldsymbol{\alpha}$ in equation 10 can then be found through kernel matrix inversion and multiplication with the corresponding reference values $\boldsymbol{y}$, equation 12

$$\boldsymbol{\alpha} = (\boldsymbol{K} + \lambda \boldsymbol{I}^{-1})\boldsymbol{y} \tag{12}$$

Where λ is the regularisation constant

Prior to the development of the DP5 probability, kernel ridge regression (KRR) models for NMR shift prediction were investigated. All KRR calculations have been performed utilising the python package qml.[20]

It has been previously shown that KRR models can be trained to reproduce NMR shielding constants predicted by DFT calculations.[19] In this study, this possibility has been pushed further utilising KRR models to predict experimentally observed NMR shifts for atoms in a molecule.

KRR models in this work were constructed and evaluated utilising the database of 5140 molecules from NMRShiftDB. The molecules are first partitioned randomly into a training and test set at a ratio of 1:20. For each carbon atom in the training set an atomic FCHL representation is then generated. The symmetric kernel matrix $\boldsymbol{K}$ for the training representations is then found utilising a gaussian kernel by equation 11. Sigma values σ of 0.5, 0.3, 0.1, 0.075, 0.05 and 0.025 were tested in this study. Once the kernel matrix has been found, equation 12 can then be solved utilising the experimental shift values corresponding to the training representations to yield the regression coefficients α. The cross kernel matrix (describing the covariances of the test and training representations) is similarly found by equation 12. Finally the experimental shifts for the test set atoms can be found by equation 10. Models were constructed and evaluated for each of these sigma values in a 20 fold cross validation study, the results are presented in Figure 8.

In order to utilise equation 11 to calculate the kernel similarity between a training and test representation, these representations must have the same dimensions. The FCHL representation relies on a matrix where the first dimension must be greater or equal to the number of atoms being represented. When generating representations for a dataset of molecules, this dimension is set as the number of atoms in the largest molecule in the dataset. However, by setting this dimension a hard limit is placed upon the number of atoms a test molecule can have for atomic property prediction. To mitigate this issue KRR models were also constructed utilising a fragmentation routine. Atomic representations are generated by first creating a molecular fragment around the central atom with a specific radius, the atomic FCHL [16] representation of the central atom is then calculated utilising this fragment. Importantly by assuming the maximum valence of the atoms in the training molecules is 4, the molecular fragments have a maximum size. When applying the model constructed in this manner, test molecule can be similarly fragmented allowing the test and training representations to have equal sizes, this allows molecules with any number of atoms to be evaluated by the model. Models were constructed and evaluated for fragment radii of r = 2,3,4 and sigma values of 0.5, 0.3, 0.1, 0.075, 0.05 and 0.025 in a 20 fold cross validation study, the results are presented in Figures 9 - 11.

All models were evaluated using a random 20 fold cross validation process.

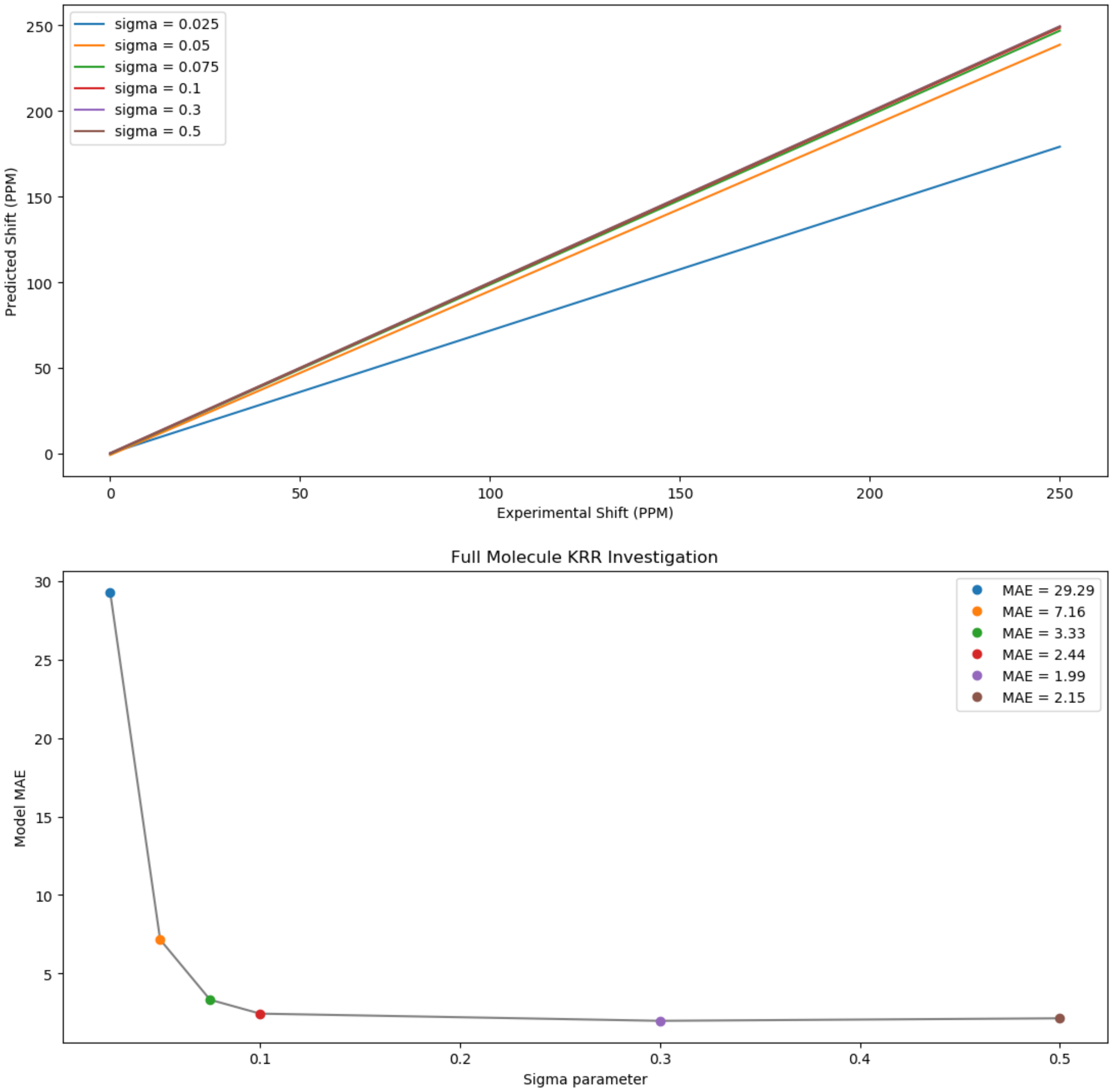

Figure 8: Top: Plot of chemical shifts predicted by KRR model against experimentally observed shift. Bottom: Model MAE against sigma. This model was trained on atomic representations with size equal to number of atoms in the largest molecule in the database, this model was evaluated at multiple sigma values. These results have been produced using a random 20 fold cross validation process.

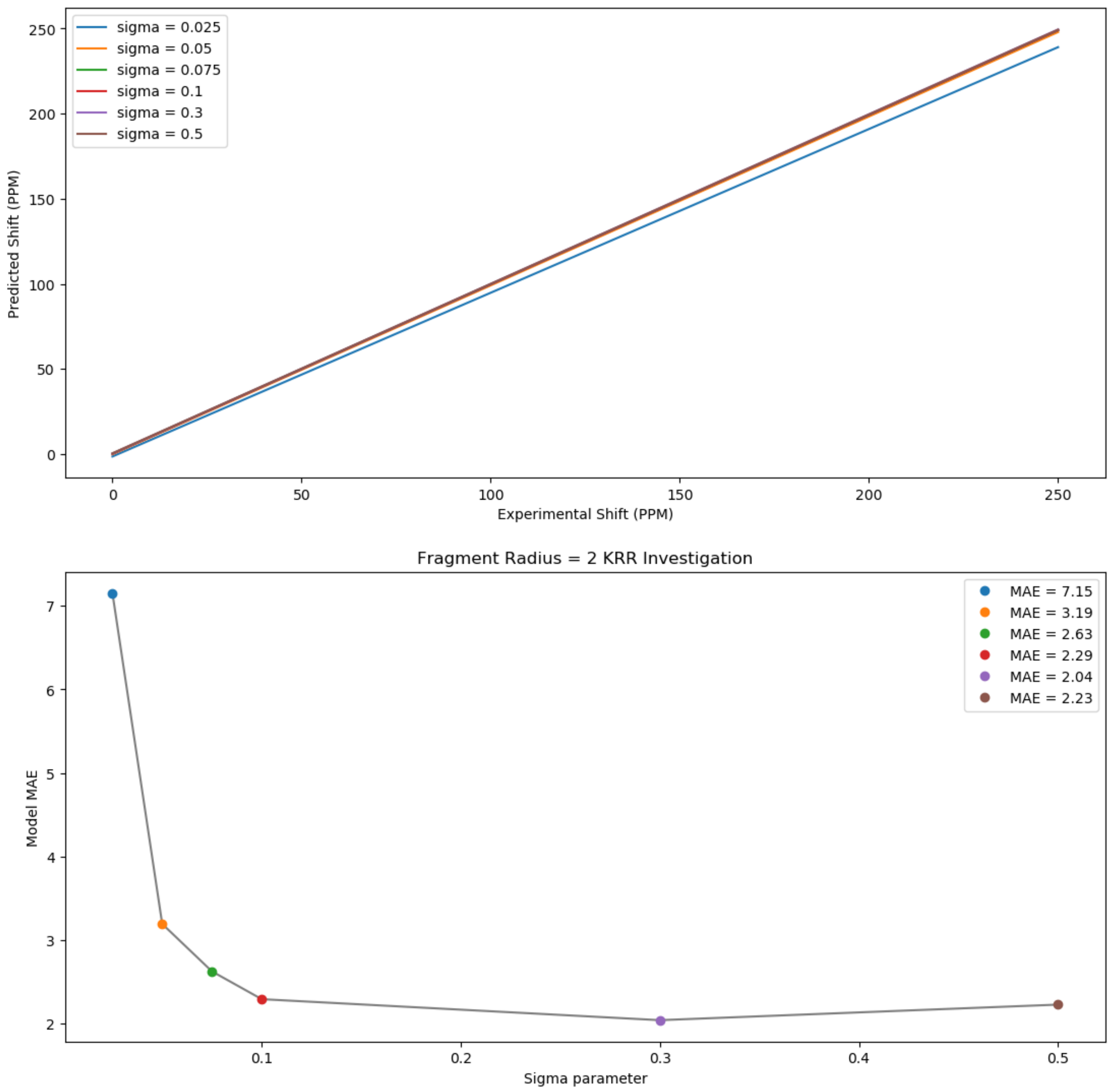

Figure 9: Top: Plot of chemical shifts predicted by KRR model against experimentally observed shift. Bottom: Model MAE against sigma. This model was trained on atomic representations generated from circular molecular fragments of radius two around a central atom. This model was evaluated at multiple sigma values. These results have been produced using a random 20 fold cross validation process.

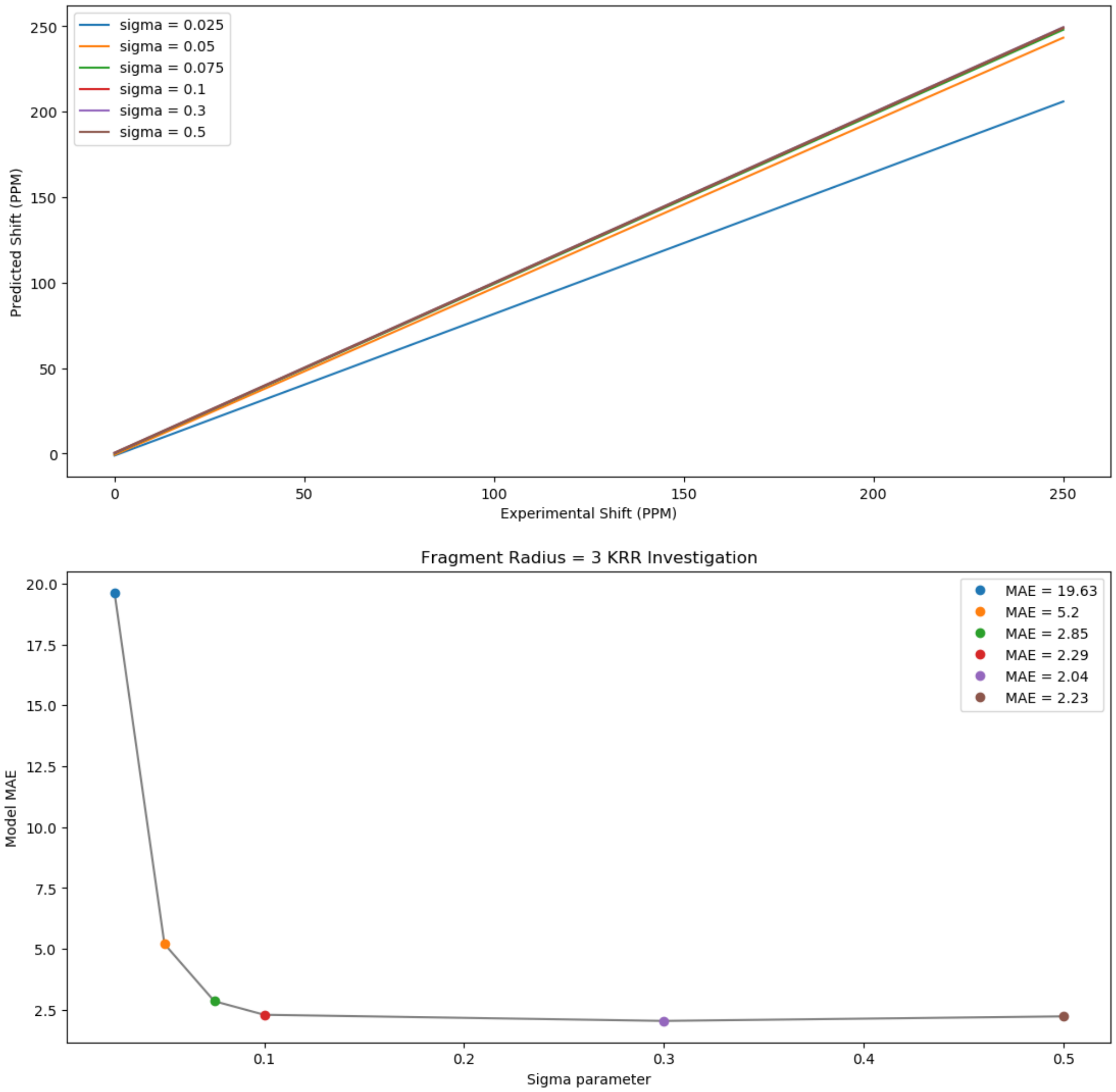

Figure 10: Top: Plot of chemical shifts predicted by KRR model against experimentally observed shift. Bottom: Model MAE against sigma. This model was trained on atomic representations generated from circular molecular fragments of radius three around a central atom. This model was evaluated at multiple sigma values. These results have been produced using a random 20 fold cross validation process.

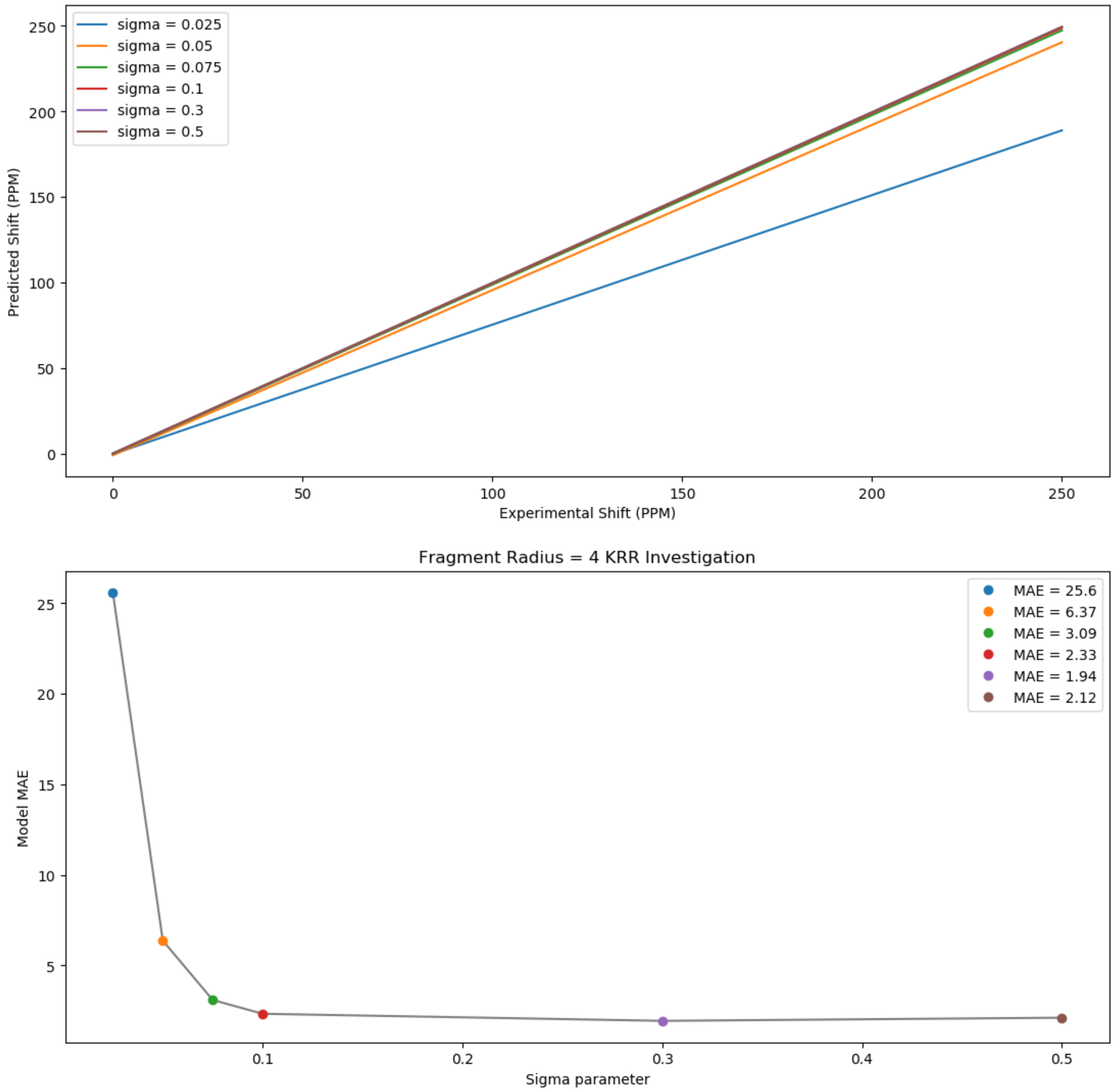

Figure 11: Top: Plot of chemical shifts predicted by KRR model against experimentally observed shift. Bottom: Model MAE against sigma. This model was trained on atomic representations generated from circular molecular fragments of radius four around a central atom. This model was evaluated at multiple sigma values. These results have been produced using a random 20 fold cross validation process.

5 Results

5.1 Stereochemistry Elucidation Results

DP5 was evaluated against a test set of 42 stereochemistry elucidation problems. This dataset of example was originally used as benchmark to evaluate DP4-AI. The molecules in this dataset are displayed in figure 12.

The full DP4 and DP5 results are displayed in figures, 13, 14 and 15

5.2 Combinatorial Study Results

Below are plots of the results from the combinatorial study described in 3.2. Each plot contains four graphs, top left: the MAE error distribution of the correct combinations (blue) and the incorrect combinations with maximum errors > 10 ppm (red). Top right: DP5 probability frequency distributions of the correct combinations (blue) and the incorrect combinations with maximum errors > 10 ppm (red). Bottom left: the MAE error distribution of the correct combinations (blue) and the MAE error distribution of incorrect combinations weighted by the corresponding probabilities from the correct combinations MAE distribution (red). Bottom right: DP5 probability frequency distribution of the correct combinations (blue) and the DP5 probability frequency distribution of the incorrect combinations when weighted by the corresponding probabilities from the correct combinations MAE distribution (red).

Plots are included for the most relevant combinations of, DP5 function (section 2.7), integration limits (section 2.5) and sigma parameter (section 2.4). A full list of plots and their corresponding formulation of the DP5 probability is given in table 16.

In addition, comparative plots for DP5 formulations differing only by the choice of sigma parameter are displayed in figures 44 - 49. These figures display three columns, Left: frequency distributions of molecular probabilities assigned to the correct (blue) and incorrect (red) combinations, where the incorrect combinations have been weighted such that their resulting MAE distribution matches that of the correct combinations. Centre: the bayesian correction function applied. Right: frequency distributions of final DP5 probabilities for correct (blue) and weighted incorrect combinations (red).

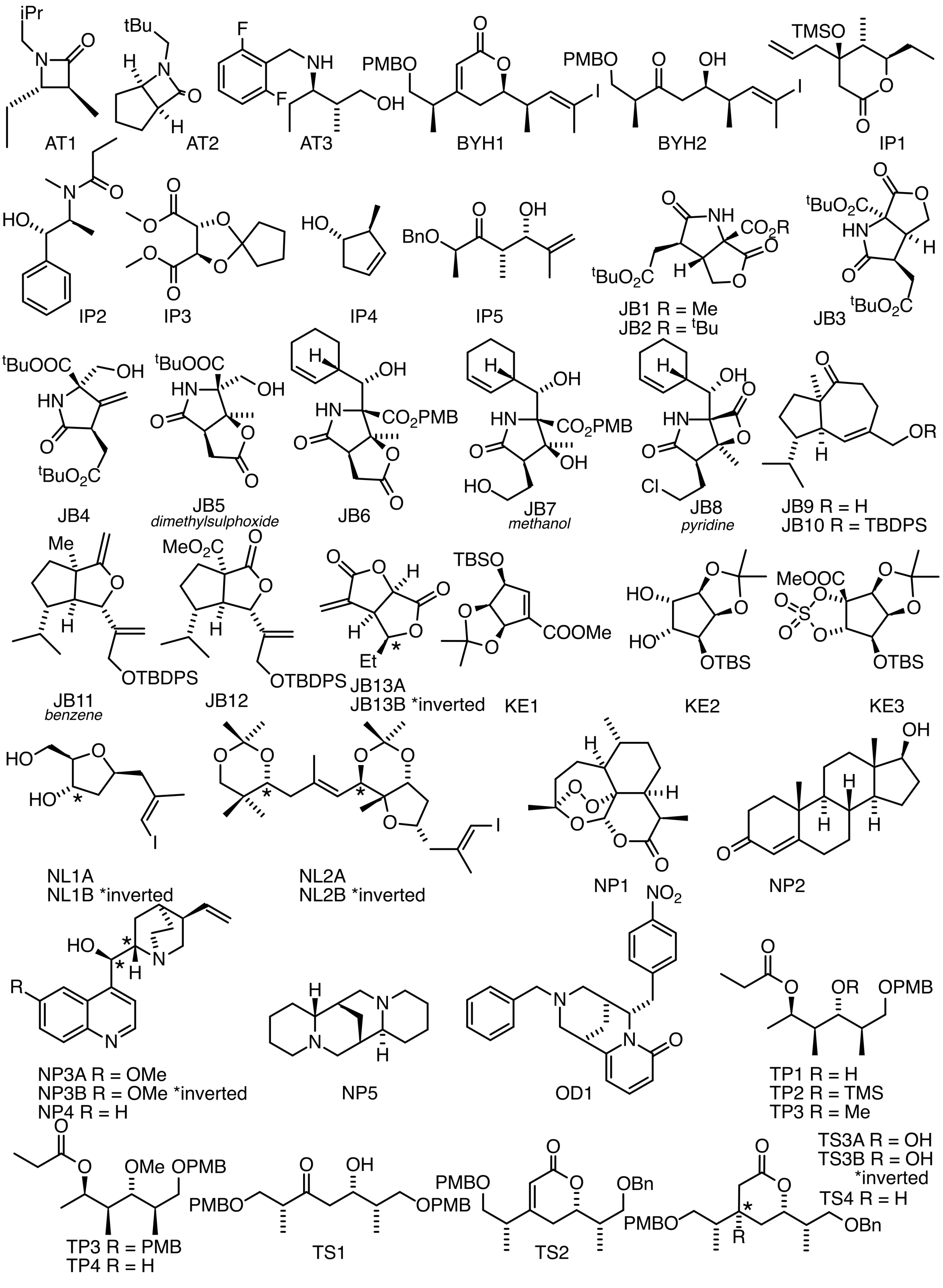

Figure 12: Figure illustrating the molecules utilised to evaluate the performance of the AA. Molecules, AT3, TS3A, TS4 and NL1A were not included as these only have corresponding 1H NMR data, all other molecules have both 1H and 13C NMR data. The spectra for molecules JB7, JB11, JB5 and JB8 were taken in solvents methanol, benzene, DMSO and methanol respectively, whilst all others were taken in $CDCl_3$. Sources for the spectral data: AT1-3,[21,22]BYH1-2,[23] JB1-13B,[24,25]KE1-3 (personal correspondence),NL1A-2B,[26] TP1-4 (personal correspondence), TS1-4(personal correspondence), data for all other molecules has been collected specifically for this study.

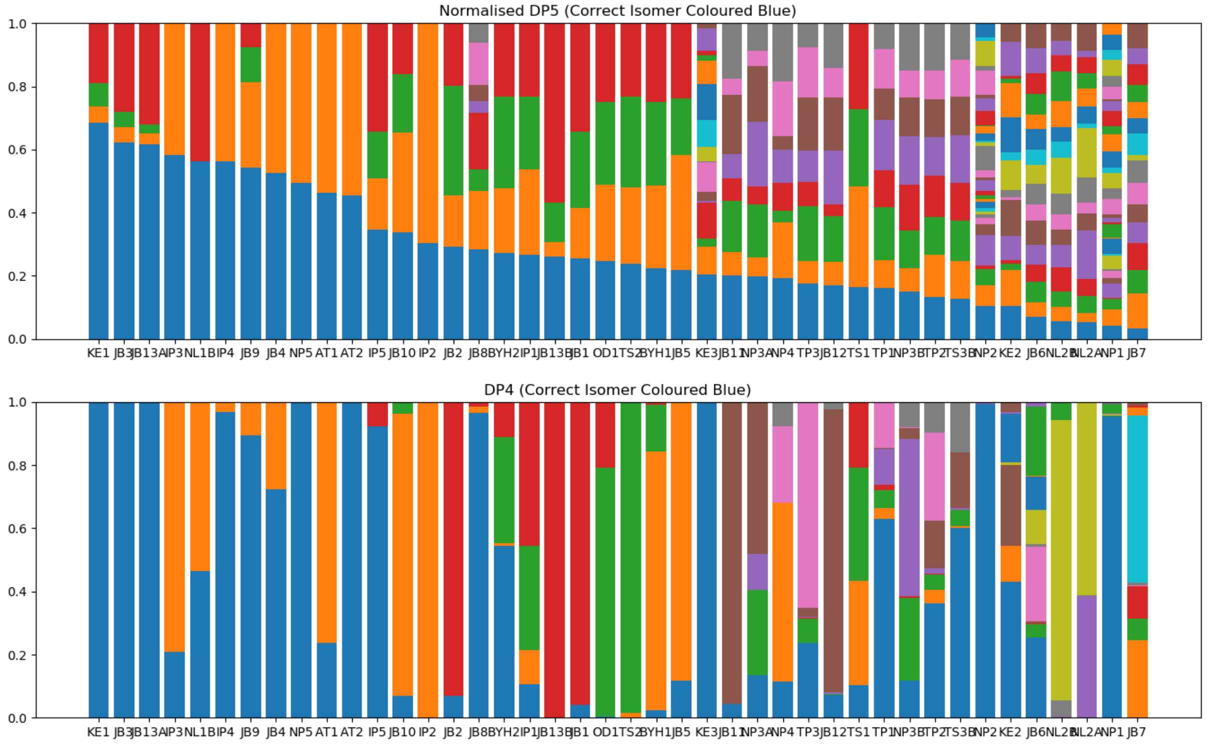

Figure 13: Full DP5 (top) and DP4 (bottom) probabilities for the 42 relative stereochemistry elucidation examples displayed in figure 12. All DP5 probabilities have been normalised to sum to 1 as in relative stereochemistry problems the correct structure can be guaranteed to be in the list of probabilities.

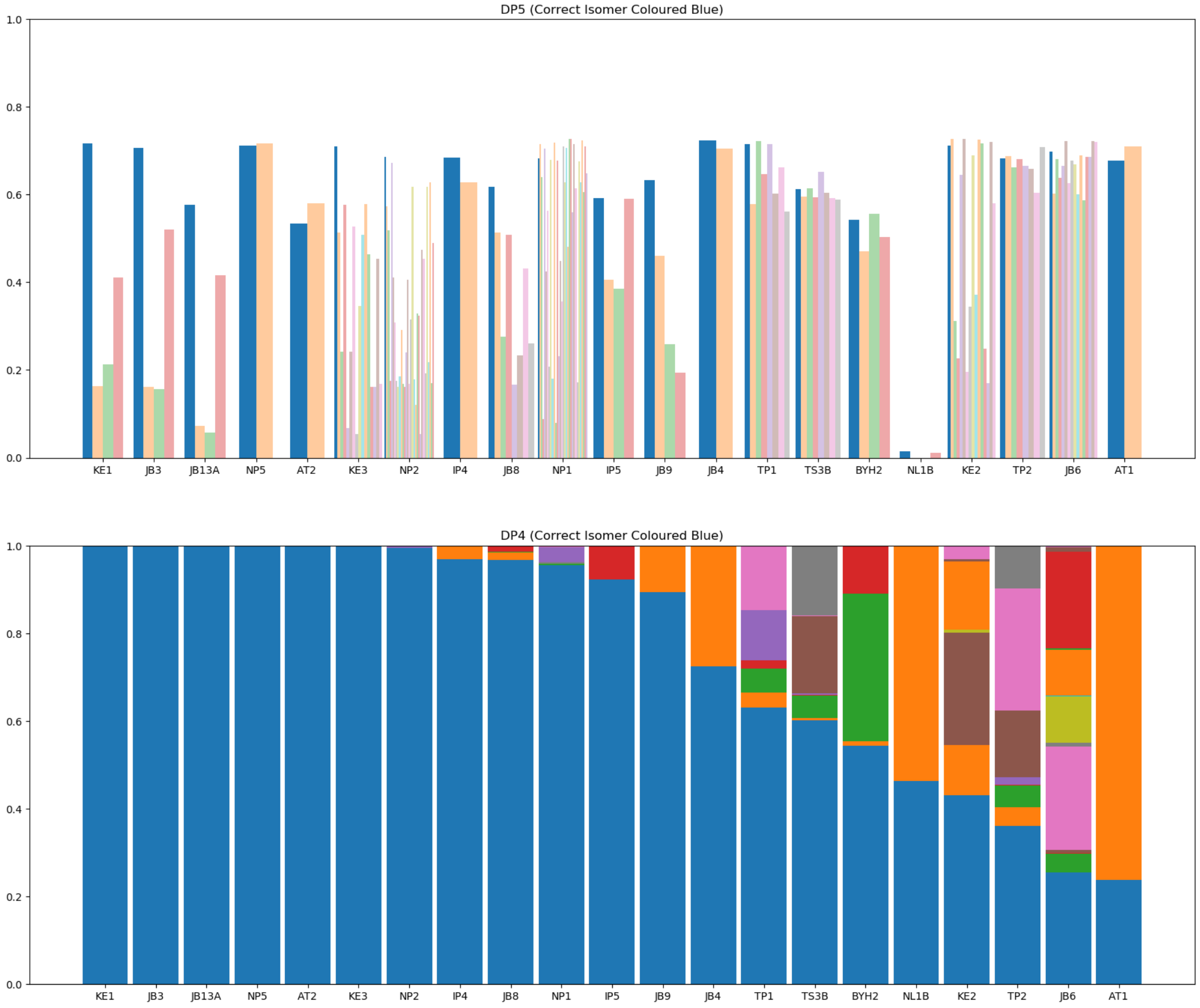

Figure 14: Full DP5 (top) and DP4 (bottom) probabilities for the 42 relative stereochemistry elucidation examples displayed in figure 12.

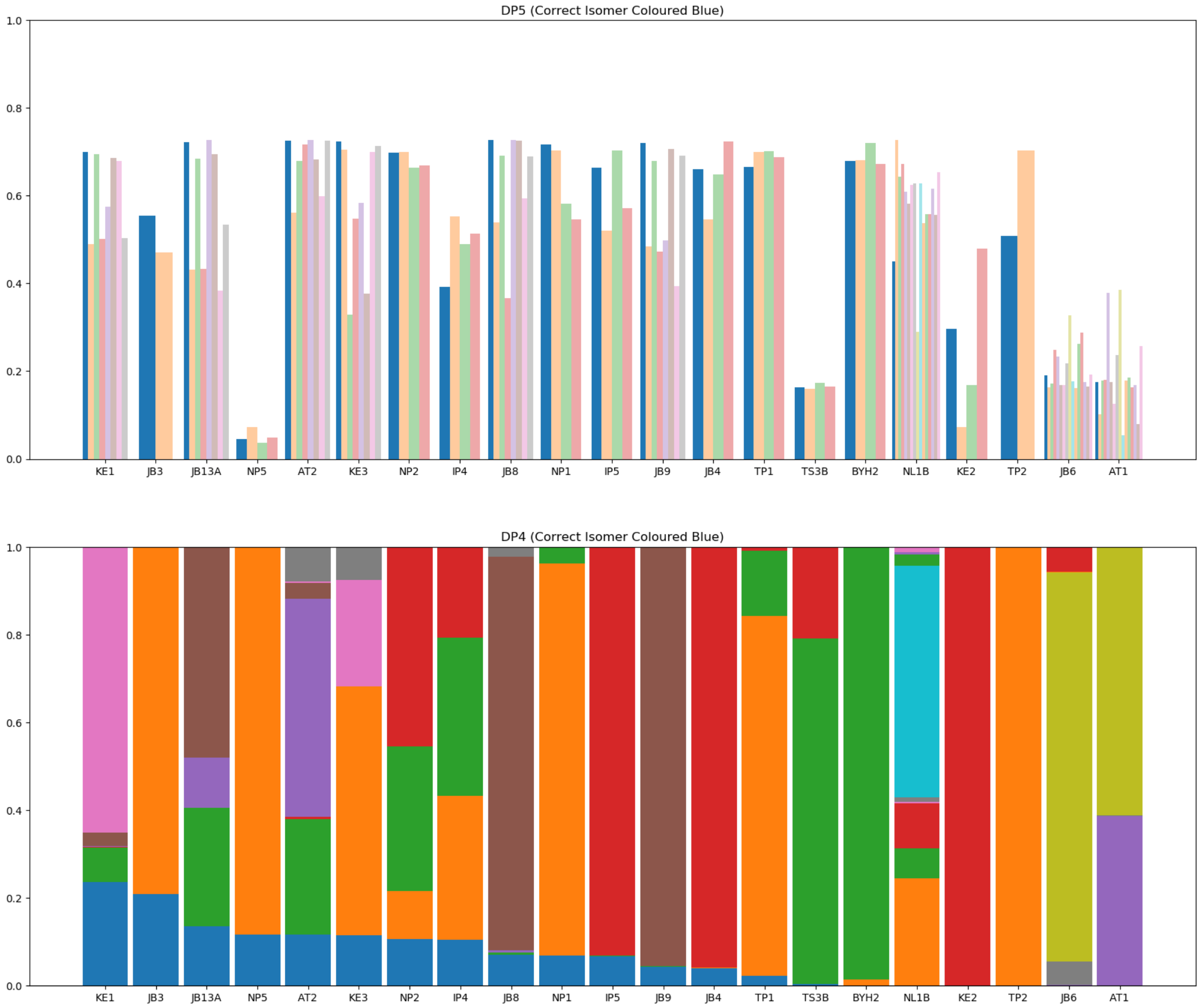

Figure 15: Full DP5 (top) and DP4 (bottom) probabilities for the 42 relative stereochemistry elucidation examples displayed in figure 12.

Figure	DP5 equation	Integration equation	Sigma Parameter
17	8	2	fixed
18	8	2	0.3
19	8	2	0.1
20	8	2	0.025
21	8	3	fixed
22	8	3	0.3
23	8	3	0.1
24	8	3	0.075
25	8	3	0.05
26	8	3	0.025
27	8	4	0.3
28	8	4	0.1
29	8	4	0.075
30	8	4	0.05
31	8	4	0.025
32	7	2	fixed
33	7	2	0.3
34	7	2	0.1
35	7	2	0.025
36	7	3	fixed
37	7	3	0.3
38	7	3	0.1
39	7	3	0.075
40	7	3	0.05
41	7	3	0.025
42	7	4	0.3
43	7	4	0.1

Figure 16

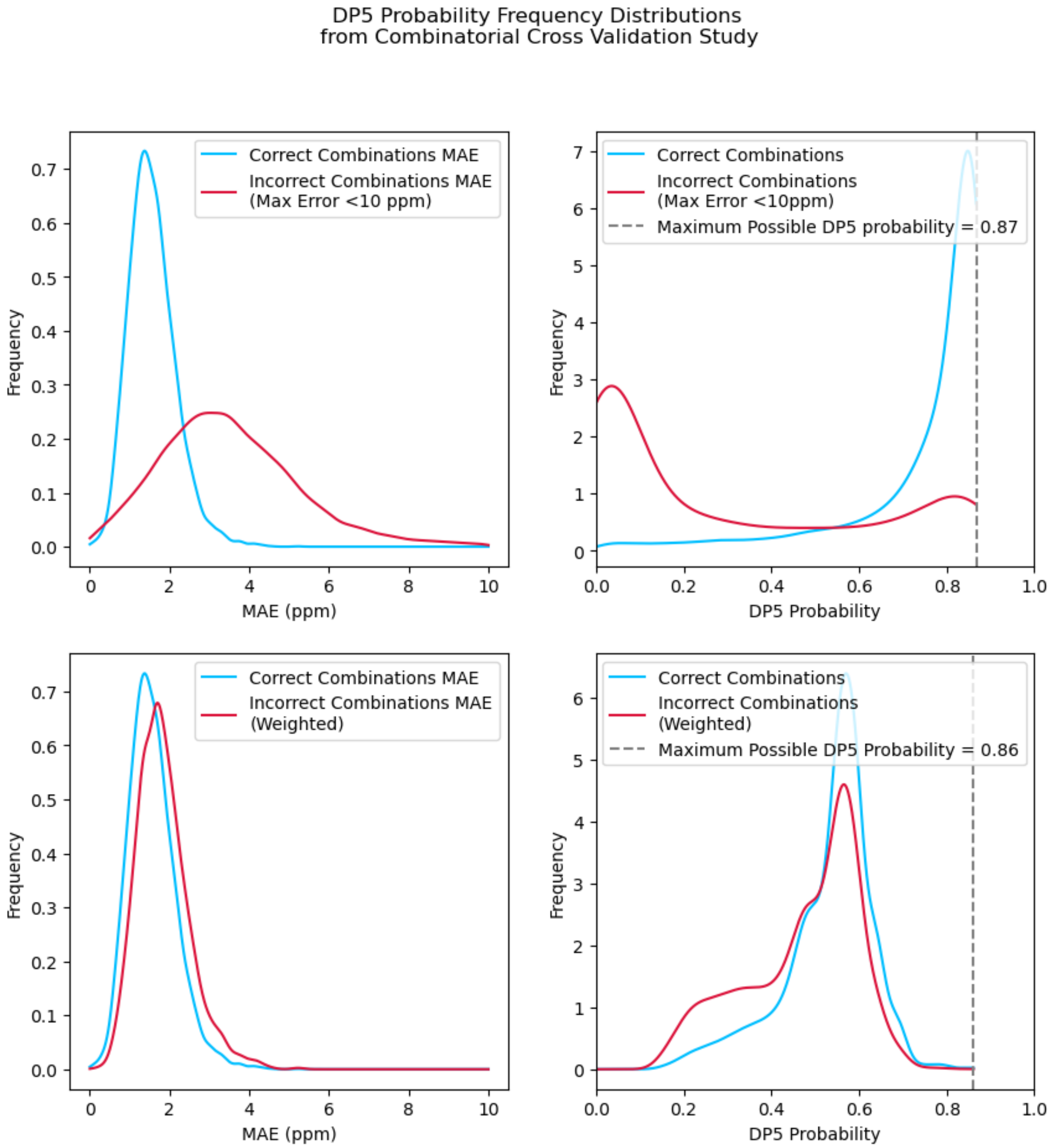

Figure 17: Figure displaying results from the combinatorial cross validation study. In this figure, the molecular probabilities were calculated using equation 8 atomic probabilities were found using equation 2 and the kernel sigma value was set to infinity. Top right: the frequency distributions of the DP5 probabilities of the correct combinations (blue) and the incorrect combinations with maximum errors > 10 ppm (red). Bottom left: the MAE error distribution of the correct combinations (blue) and the MAE error distribution of incorrect combinations weighted by the corresponding probabilities from the correct combinations MAE distribution (red). Bottom right: the frequency distributions of the DP5 probabilities of the correct combinations (blue) and weighted frequency distribution of the DP5 probabilities of the incorrect combinations weighted by the corresponding probabilities from the correct combinations MAE distribution (red).

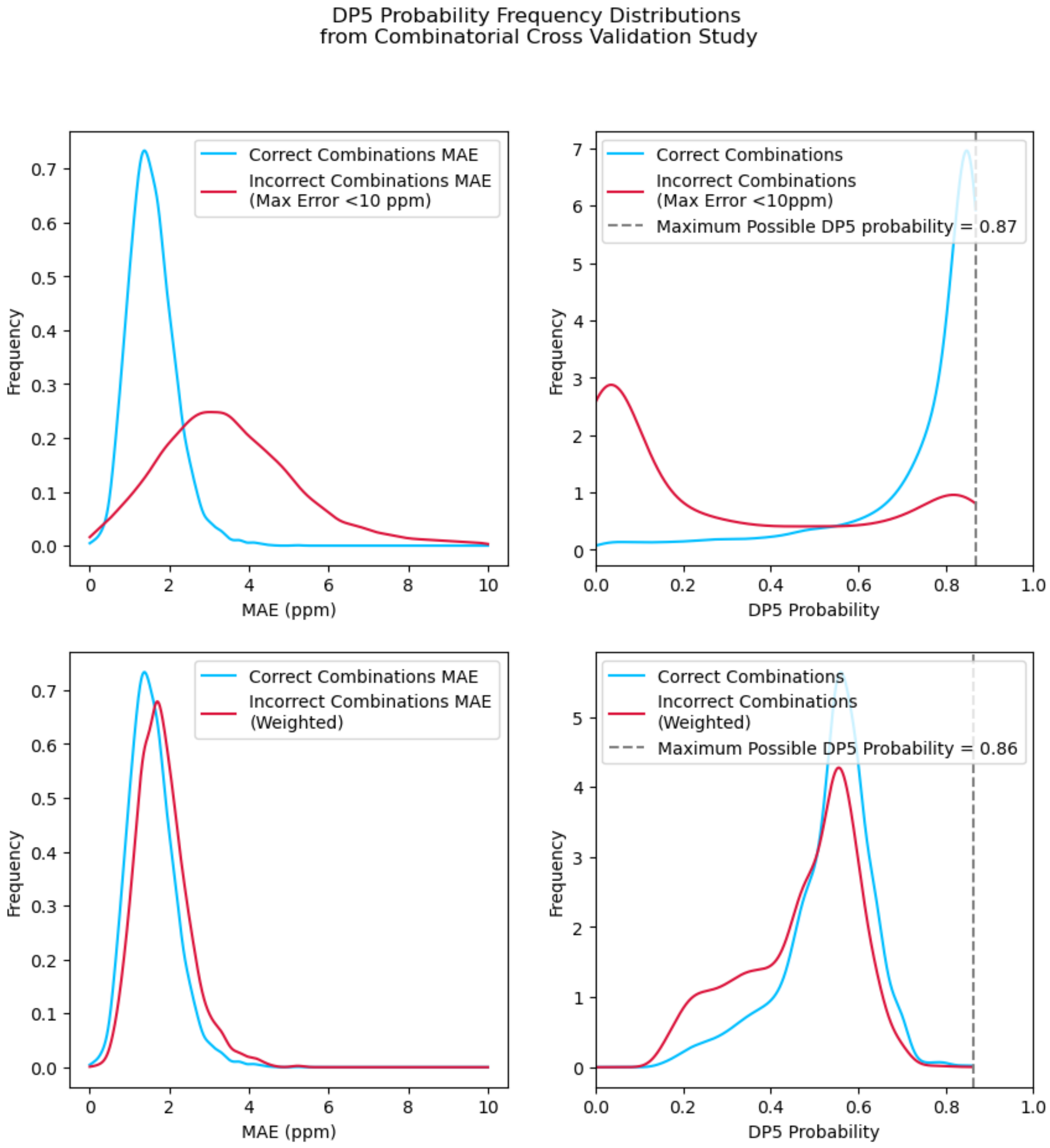

Figure 18: Figure displaying results from the combinatorial cross validation study. In this figure, the molecular probabilities were calculated using equation 8 atomic probabilities were found using equation 2 and the kernel sigma value was set to 0.3. Top right: the frequency distributions of the DP5 probabilities of the correct combinations (blue) and the incorrect combinations with maximum errors > 10 ppm (red). Bottom left: the MAE error distribution of the correct combinations (blue) and the MAE error distribution of incorrect combinations weighted by the corresponding probabilities from the correct combinations MAE distribution (red). Bottom right: the frequency distributions of the DP5 probabilities of the correct combinations (blue) and weighted frequency distribution of the DP5 probabilities of the incorrect combinations weighted by the corresponding probabilities from the correct combinations MAE distribution (red).

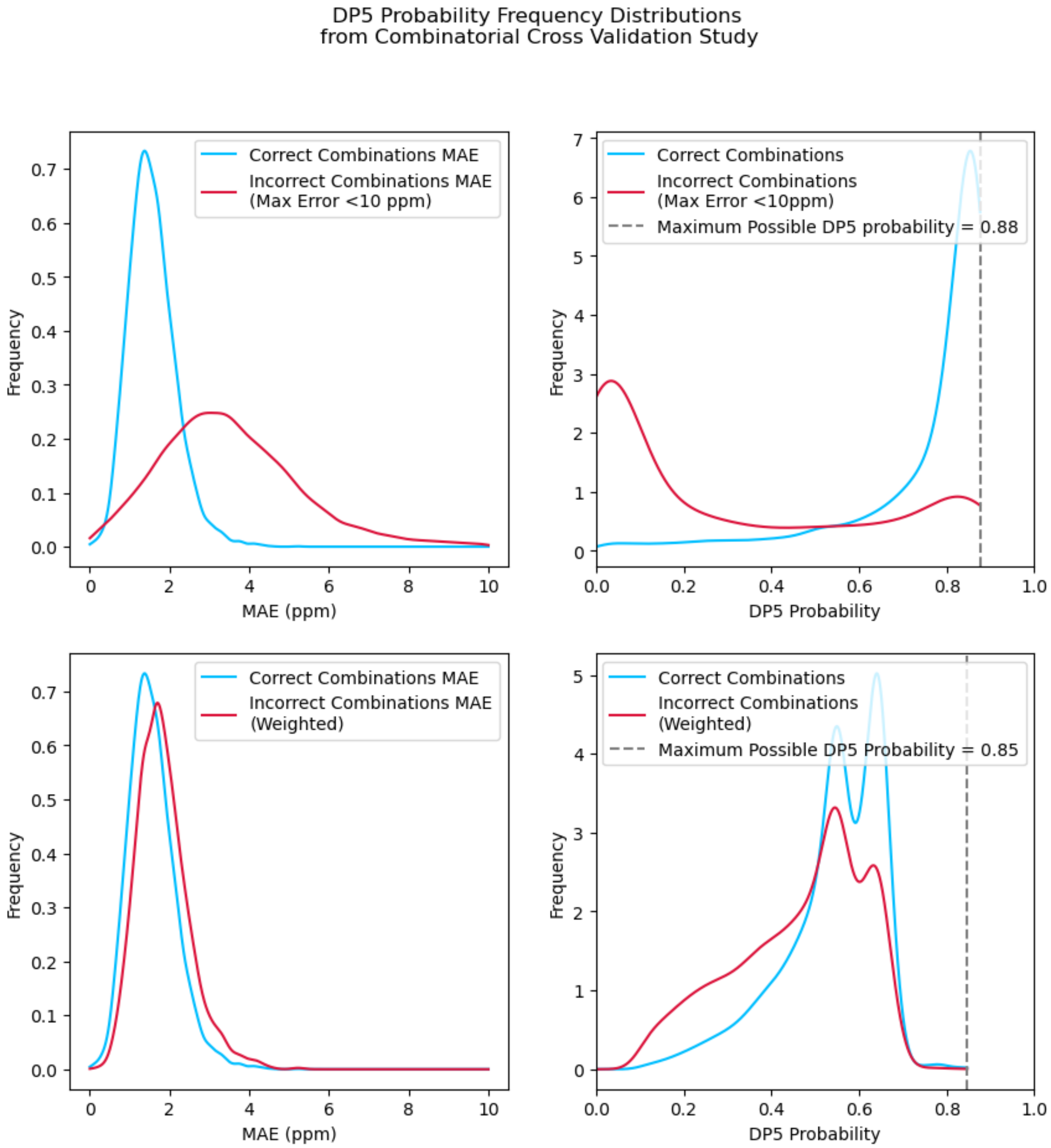

Figure 19: Figure displaying results from the combinatorial cross validation study. In this figure, the molecular probabilities were calculated using equation 8 atomic probabilities were found using equation 2 and the kernel sigma value was set to 0.1. Top right: the frequency distributions of the DP5 probabilities of the correct combinations (blue) and the incorrect combinations with maximum errors $>$ 10 ppm (red). Bottom left: the MAE error distribution of the correct combinations (blue) and the MAE error distribution of incorrect combinations weighted by the corresponding probabilities from the correct combinations MAE distribution (red). Bottom right: the frequency distributions of the DP5 probabilities of the correct combinations (blue) and weighted frequency distribution of the DP5 probabilities of the incorrect combinations weighted by the corresponding probabilities from the correct combinations MAE distribution (red).

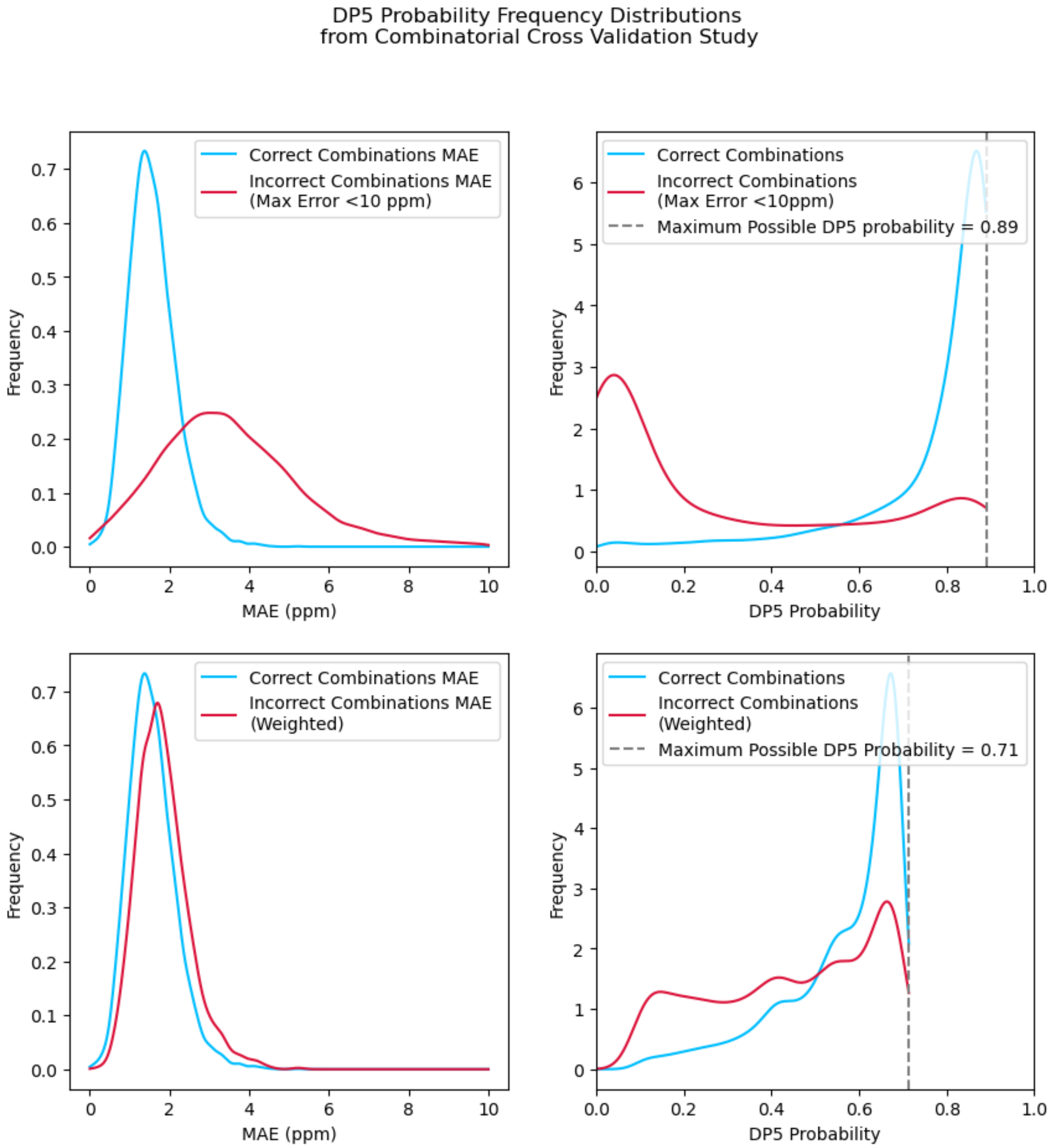

Figure 20: Figure displaying results from the combinatorial cross validation study. In this figure, the molecular probabilities were calculated using equation 8 atomic probabilities were found using equation 2 and the kernel sigma value was set to 0.025. Top right: the frequency distributions of the DP5 probabilities of the correct combinations (blue) and the incorrect combinations with maximum errors $>$ 10 ppm (red). Bottom left: the MAE error distribution of the correct combinations (blue) and the MAE error distribution of incorrect combinations weighted by the corresponding probabilities from the correct combinations MAE distribution (red). Bottom right: the frequency distributions of the DP5 probabilities of the correct combinations (blue) and weighted frequency distribution of the DP5 probabilities of the incorrect combinations weighted by the corresponding probabilities from the correct combinations MAE distribution (red).

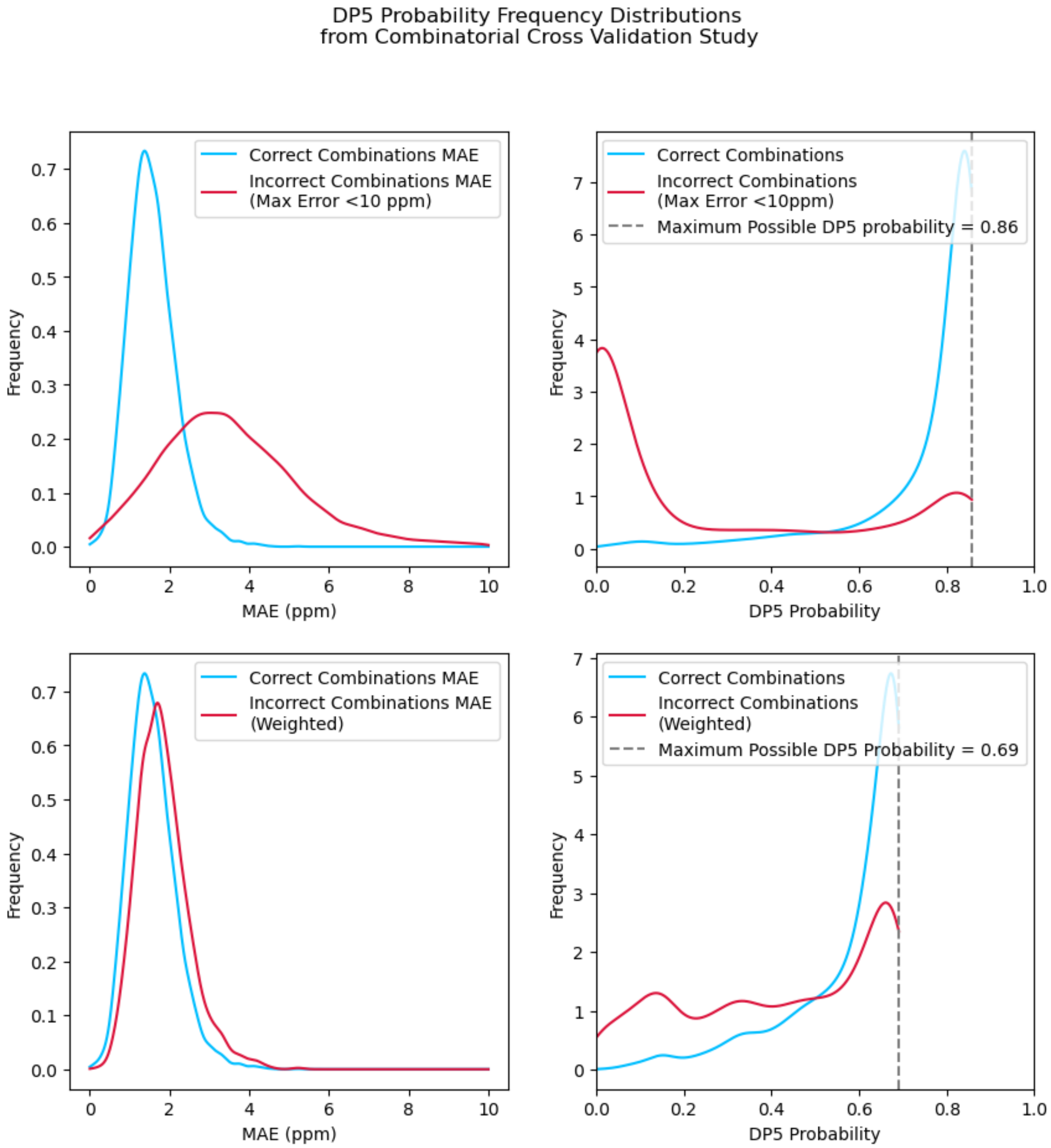

Figure 21: Figure displaying results from the combinatorial cross validation study. In this figure, the molecular probabilities were calculated using equation 8 atomic probabilities were found using equation 3 and the kernel sigma value was set to infinity. Top right: the frequency distributions of the DP5 probabilities of the correct combinations (blue) and the incorrect combinations with maximum errors > 10 ppm (red). Bottom left: the MAE error distribution of the correct combinations (blue) and the MAE error distribution of incorrect combinations weighted by the corresponding probabilities from the correct combinations MAE distribution (red). Bottom right: the frequency distributions of the DP5 probabilities of the correct combinations (blue) and weighted frequency distribution of the DP5 probabilities of the incorrect combinations weighted by the corresponding probabilities from the correct combinations MAE distribution (red).

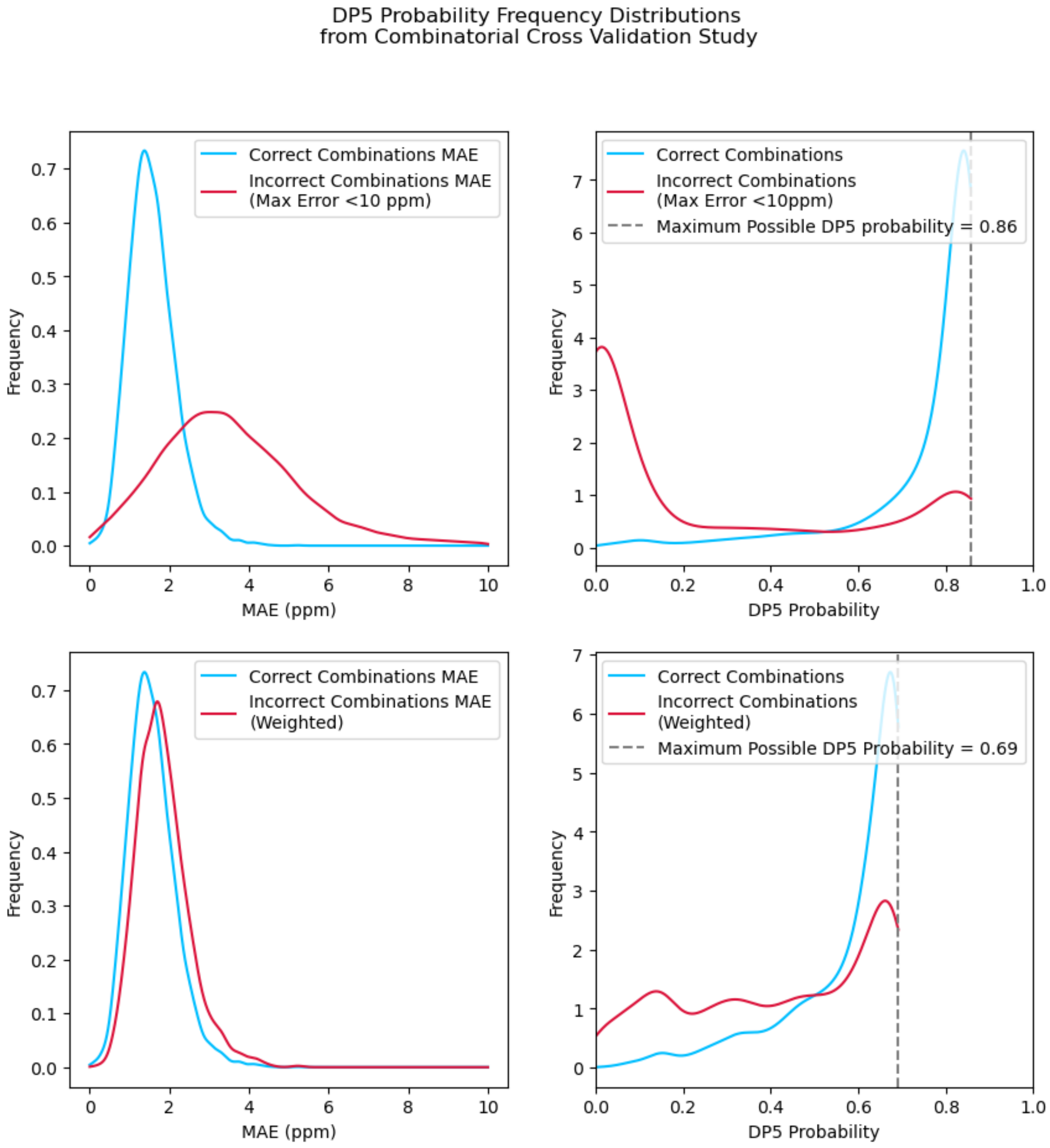

Figure 22: Figure displaying results from the combinatorial cross validation study. In this figure, the molecular probabilities were calculated using equation8 atomic probabilities were found using equation 3 and the kernel sigma value was set to 0.3. Top right: the frequency distributions of the DP5 probabilities of the correct combinations (blue) and the incorrect combinations with maximum errors $>$ 10 ppm (red). Bottom left: the MAE error distribution of the correct combinations (blue) and the MAE error distribution of incorrect combinations weighted by the corresponding probabilities from the correct combinations MAE distribution (red). Bottom right: the frequency distributions of the DP5 probabilities of the correct combinations (blue) and weighted frequency distribution of the DP5 probabilities of the incorrect combinations weighted by the corresponding probabilities from the correct combinations MAE distribution (red).

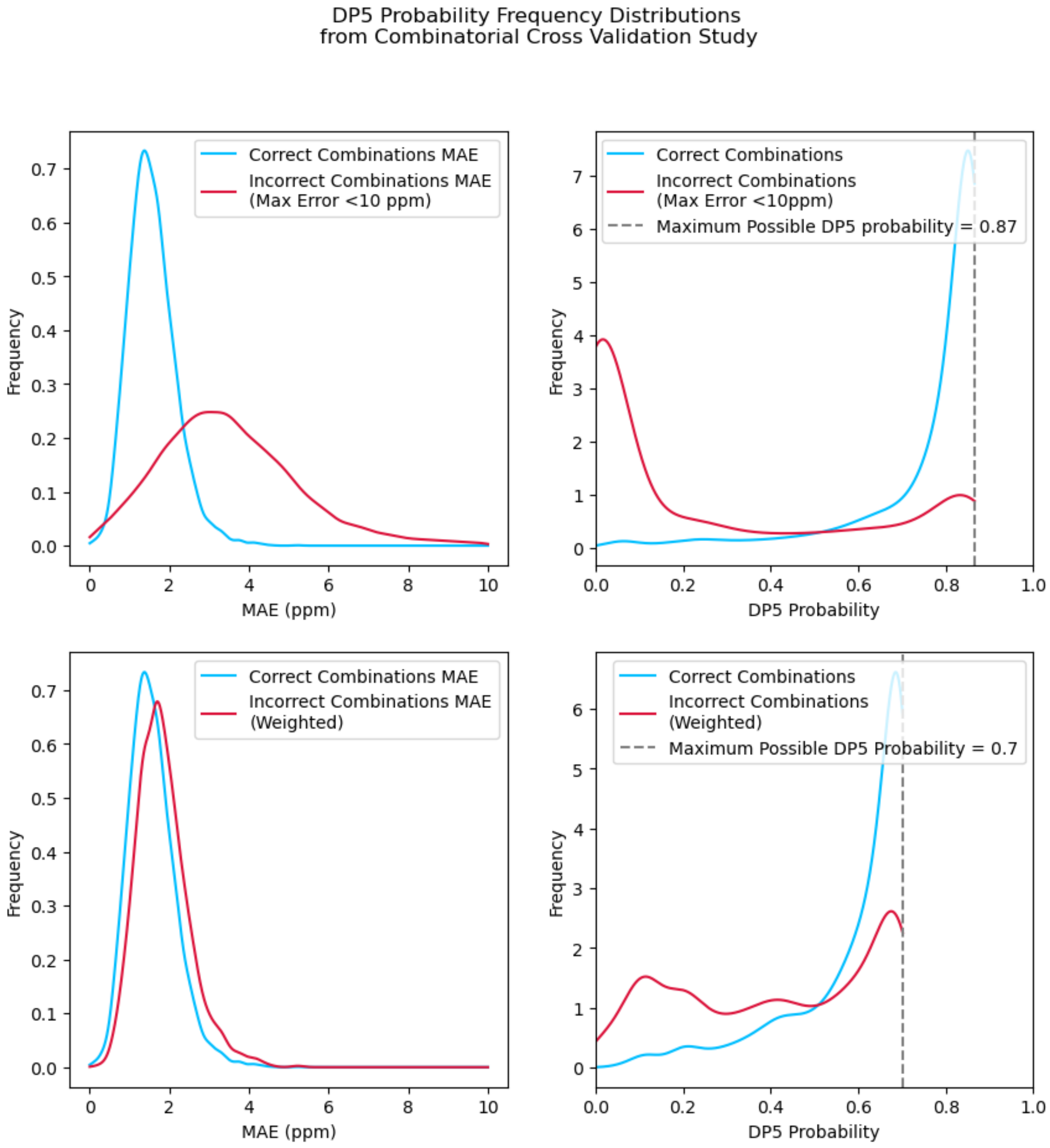

Figure 23: Figure displaying results from the combinatorial cross validation study. In this figure, the molecular probabilities were calculated using equation 8 atomic probabilities were found using equation 3 and the kernel sigma value was set to 0.1. Top right: the frequency distributions of the DP5 probabilities of the correct combinations (blue) and the incorrect combinations with maximum errors > 10 ppm (red). Bottom left: the MAE error distribution of the correct combinations (blue) and the MAE error distribution of incorrect combinations weighted by the corresponding probabilities from the correct combinations MAE distribution (red). Bottom right: the frequency distributions of the DP5 probabilities of the correct combinations (blue) and weighted frequency distribution of the DP5 probabilities of the incorrect combinations weighted by the corresponding probabilities from the correct combinations MAE distribution (red).

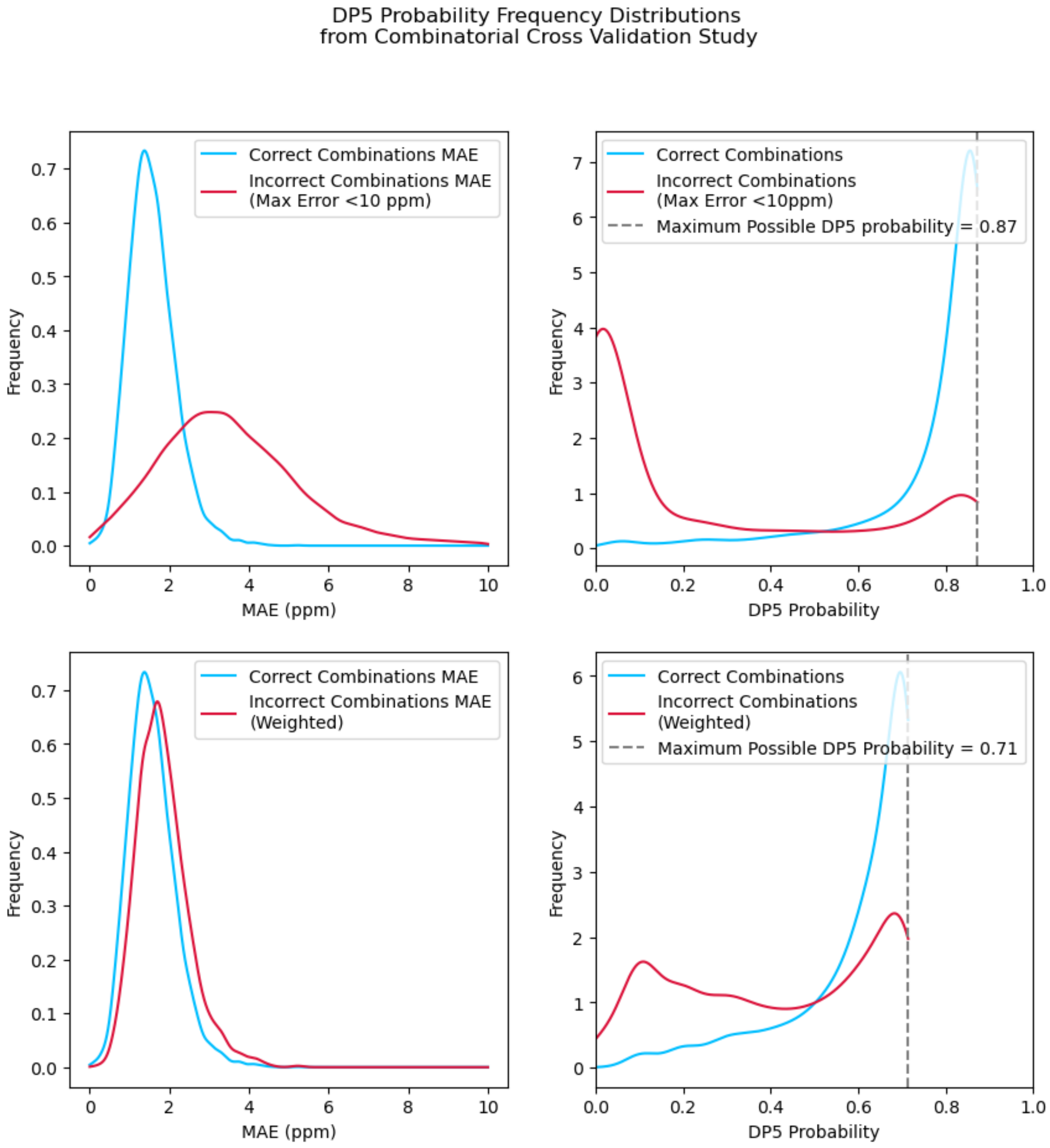

Figure 24: Figure displaying results from the combinatorial cross validation study. In this figure, the molecular probabilities were calculated using equation 8 atomic probabilities were found using equation 3 and the kernel sigma value was set to 0.075. Top right: the frequency distributions of the DP5 probabilities of the correct combinations (blue) and the incorrect combinations with maximum errors $>$ 10 ppm (red). Bottom left: the MAE error distribution of the correct combinations (blue) and the MAE error distribution of incorrect combinations weighted by the corresponding probabilities from the correct combinations MAE distribution (red). Bottom right: the frequency distributions of the DP5 probabilities of the correct combinations (blue) and weighted frequency distribution of the DP5 probabilities of the incorrect combinations weighted by the corresponding probabilities from the correct combinations MAE distribution (red).

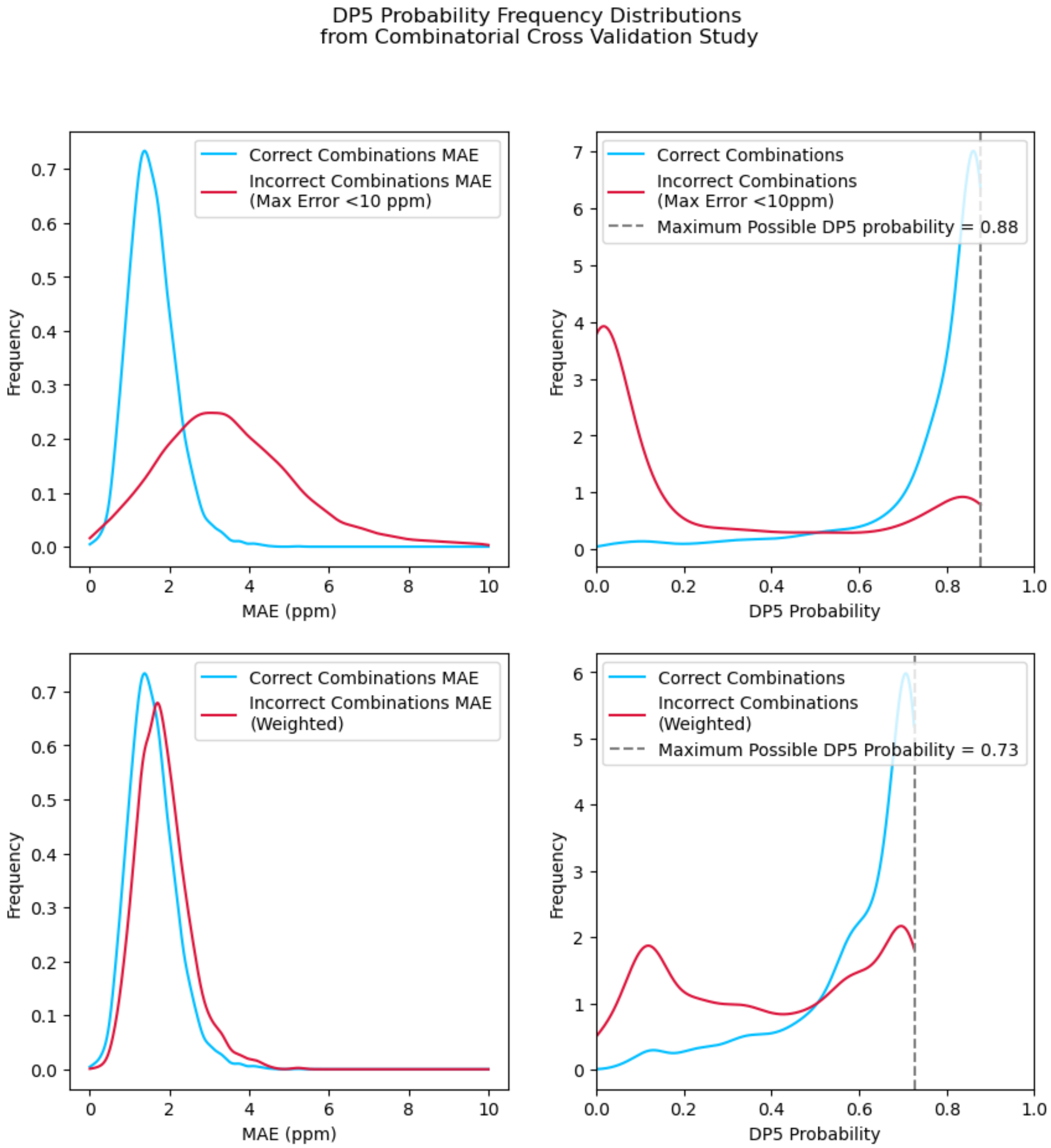

Figure 25: Figure displaying results from the combinatorial cross validation study. In this figure, the molecular probabilities were calculated using equation 8 atomic probabilities were found using equation 3 and the kernel sigma value was set to 0.05. Top right: the frequency distributions of the DP5 probabilities of the correct combinations (blue) and the incorrect combinations with maximum errors $>$ 10 ppm (red). Bottom left: the MAE error distribution of the correct combinations (blue) and the MAE error distribution of incorrect combinations weighted by the corresponding probabilities from the correct combinations MAE distribution (red). Bottom right: the frequency distributions of the DP5 probabilities of the correct combinations (blue) and weighted frequency distribution of the DP5 probabilities of the incorrect combinations weighted by the corresponding probabilities from the correct combinations MAE distribution (red).

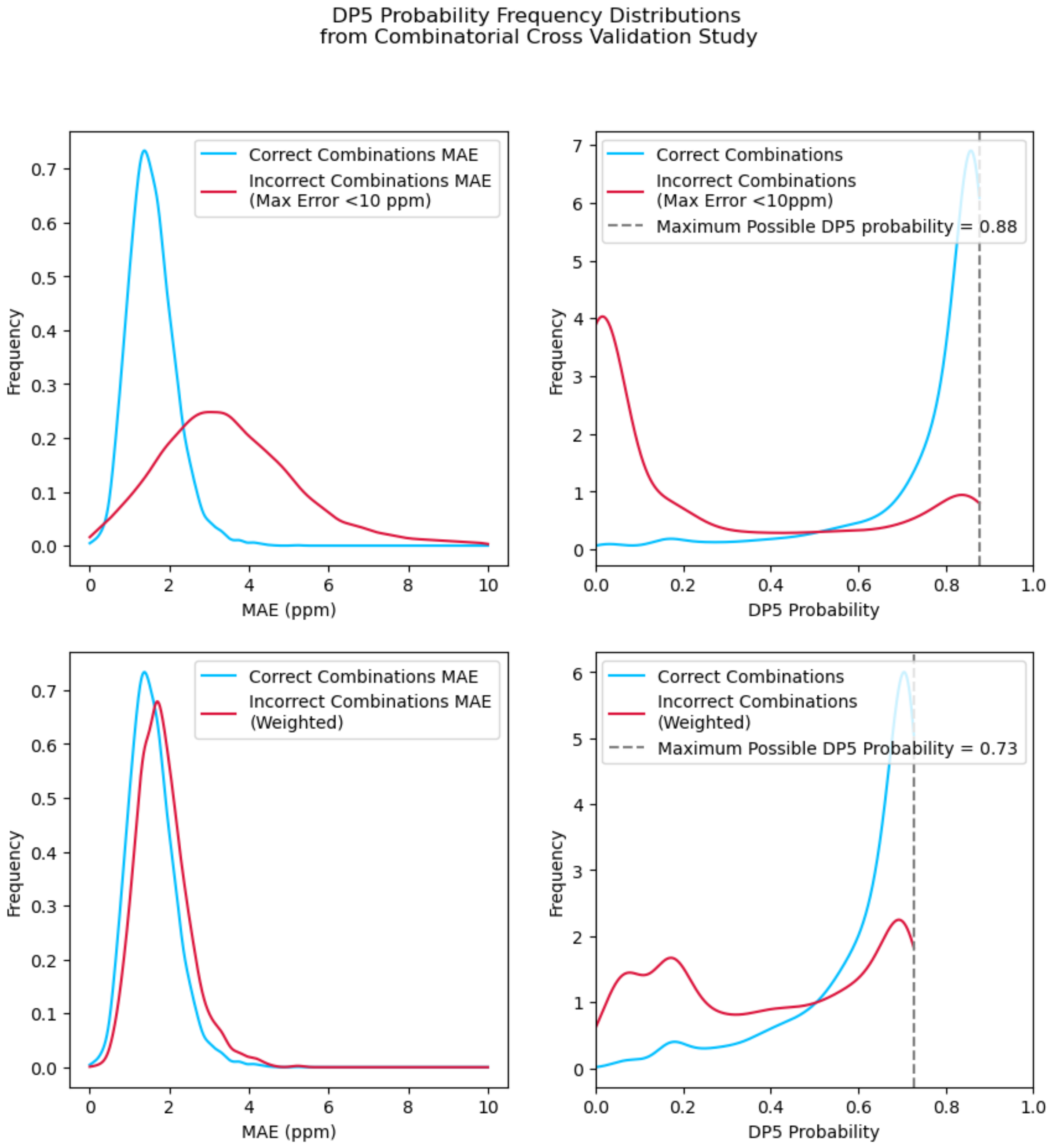

Figure 26: Figure displaying results from the combinatorial cross validation study. In this figure, the molecular probabilities were calculated using equation 8 atomic probabilities were found using equation 3 and the kernel sigma value was set to 0.025. Top right: the frequency distributions of the DP5 probabilities of the correct combinations (blue) and the incorrect combinations with maximum errors > 10 ppm (red). Bottom left: the MAE error distribution of the correct combinations (blue) and the MAE error distribution of incorrect combinations weighted by the corresponding probabilities from the correct combinations MAE distribution (red). Bottom right: the frequency distributions of the DP5 probabilities of the correct combinations (blue) and weighted frequency distribution of the DP5 probabilities of the incorrect combinations weighted by the corresponding probabilities from the correct combinations MAE distribution (red).

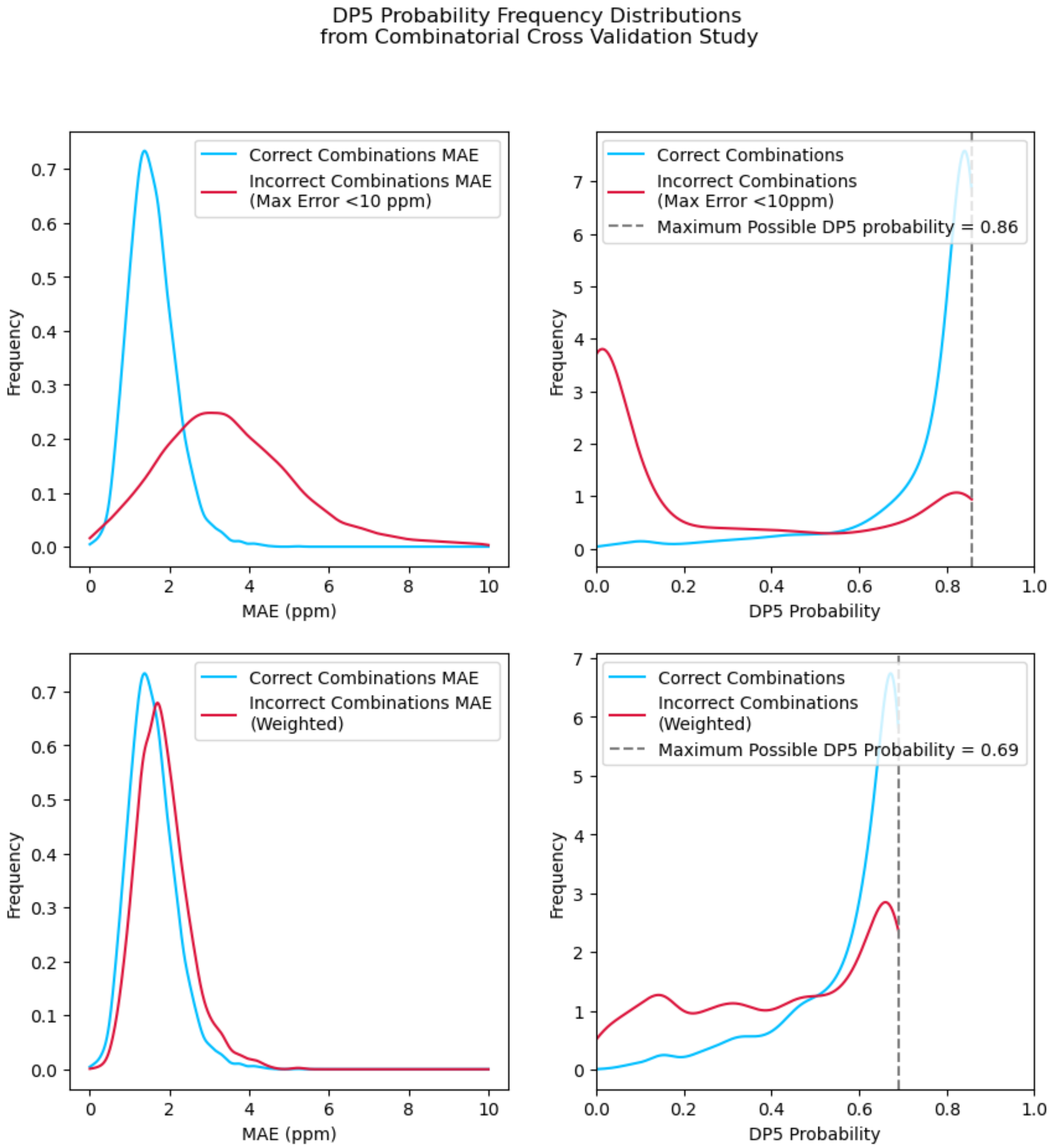

Figure 27: Figure displaying results from the combinatorial cross validation study. In this figure, the molecular probabilities were calculated using equation 8 atomic probabilities were found using equation 4 and the kernel sigma value was set to 0.3. Top right: the frequency distributions of the DP5 probabilities of the correct combinations (blue) and the incorrect combinations with maximum errors > 10 ppm (red). Bottom left: the MAE error distribution of the correct combinations (blue) and the MAE error distribution of incorrect combinations weighted by the corresponding probabilities from the correct combinations MAE distribution (red). Bottom right: the frequency distributions of the DP5 probabilities of the correct combinations (blue) and weighted frequency distribution of the DP5 probabilities of the incorrect combinations weighted by the corresponding probabilities from the correct combinations MAE distribution (red).

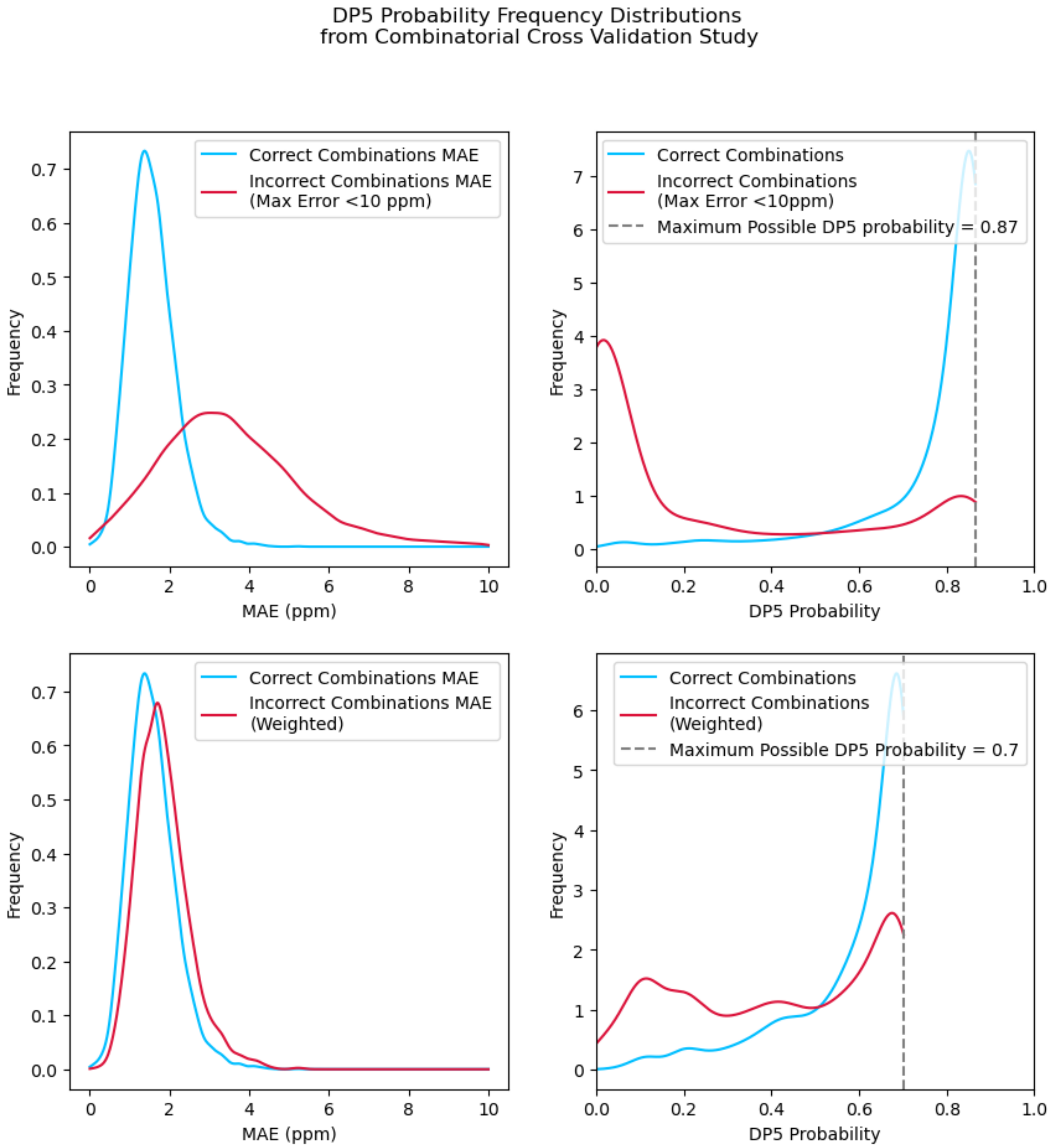

Figure 28: Figure displaying results from the combinatorial cross validation study. In this figure, the molecular probabilities were calculated using equation 8 atomic probabilities were found using equation 4 and the kernel sigma value was set to 0.1. Top right: the frequency distributions of the DP5 probabilities of the correct combinations (blue) and the incorrect combinations with maximum errors > 10 ppm (red). Bottom left: the MAE error distribution of the correct combinations (blue) and the MAE error distribution of incorrect combinations weighted by the corresponding probabilities from the correct combinations MAE distribution (red). Bottom right: the frequency distributions of the DP5 probabilities of the correct combinations (blue) and weighted frequency distribution of the DP5 probabilities of the incorrect combinations weighted by the corresponding probabilities from the correct combinations MAE distribution (red).

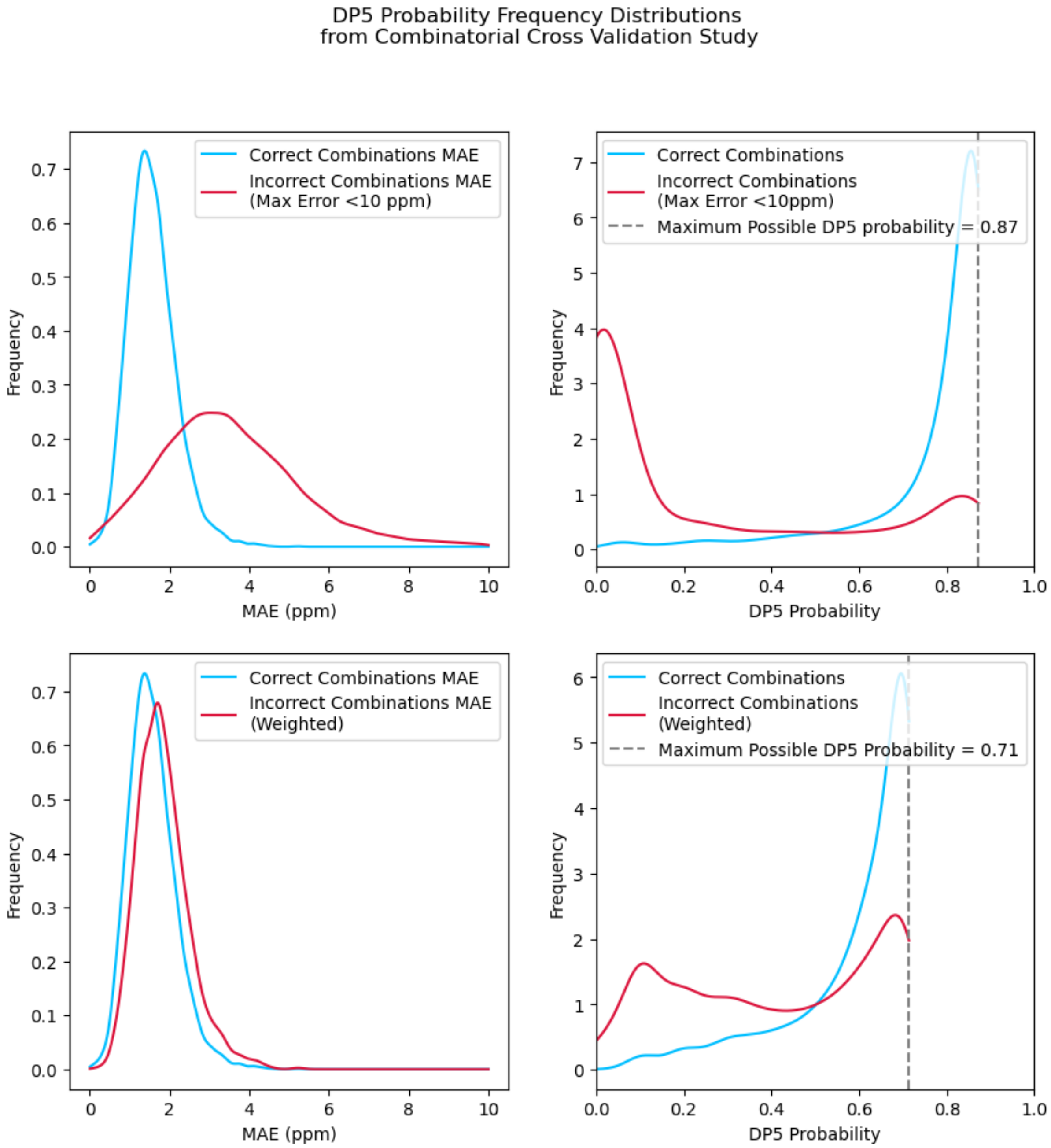

Figure 29: Figure displaying results from the combinatorial cross validation study. In this figure, the molecular probabilities were calculated using equation 8 atomic probabilities were found using equation 4 and the kernel sigma value was set to 0.075. Top right: the frequency distributions of the DP5 probabilities of the correct combinations (blue) and the incorrect combinations with maximum errors > 10 ppm (red). Bottom left: the MAE error distribution of the correct combinations (blue) and the MAE error distribution of incorrect combinations weighted by the corresponding probabilities from the correct combinations MAE distribution (red). Bottom right: the frequency distributions of the DP5 probabilities of the correct combinations (blue) and weighted frequency distribution of the DP5 probabilities of the incorrect combinations weighted by the corresponding probabilities from the correct combinations MAE distribution (red).

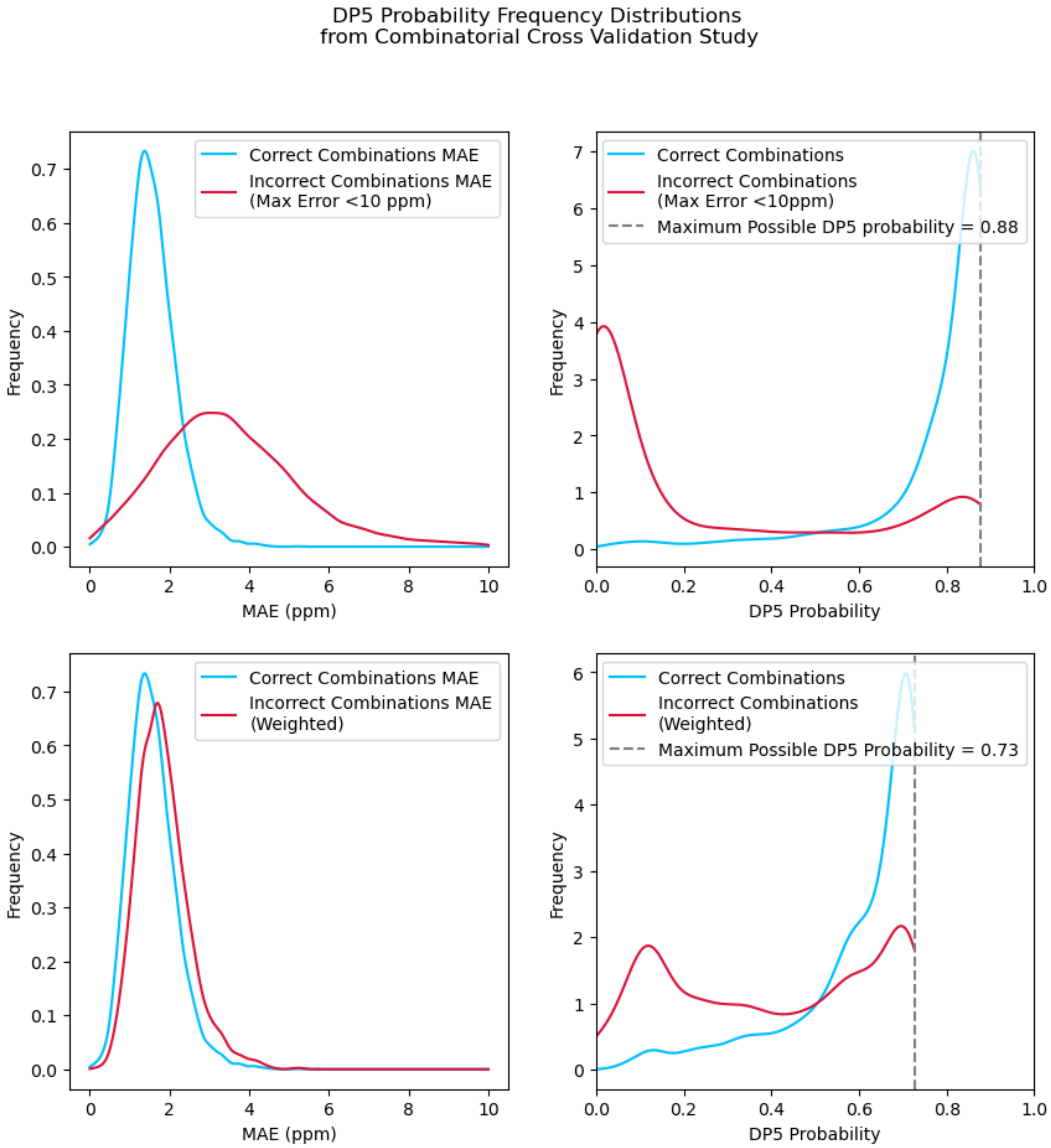

Figure 30: Figure displaying results from the combinatorial cross validation study. In this figure, the molecular probabilities were calculated using equation 8 atomic probabilities were found using equation 4 and the kernel sigma value was set to 0.05. Top right: the frequency distributions of the DP5 probabilities of the correct combinations (blue) and the incorrect combinations with maximum errors > 10 ppm (red). Bottom left: the MAE error distribution of the correct combinations (blue) and the MAE error distribution of incorrect combinations weighted by the corresponding probabilities from the correct combinations MAE distribution (red). Bottom right: the frequency distributions of the DP5 probabilities of the correct combinations (blue) and weighted frequency distribution of the DP5 probabilities of the incorrect combinations weighted by the corresponding probabilities from the correct combinations MAE distribution (red).

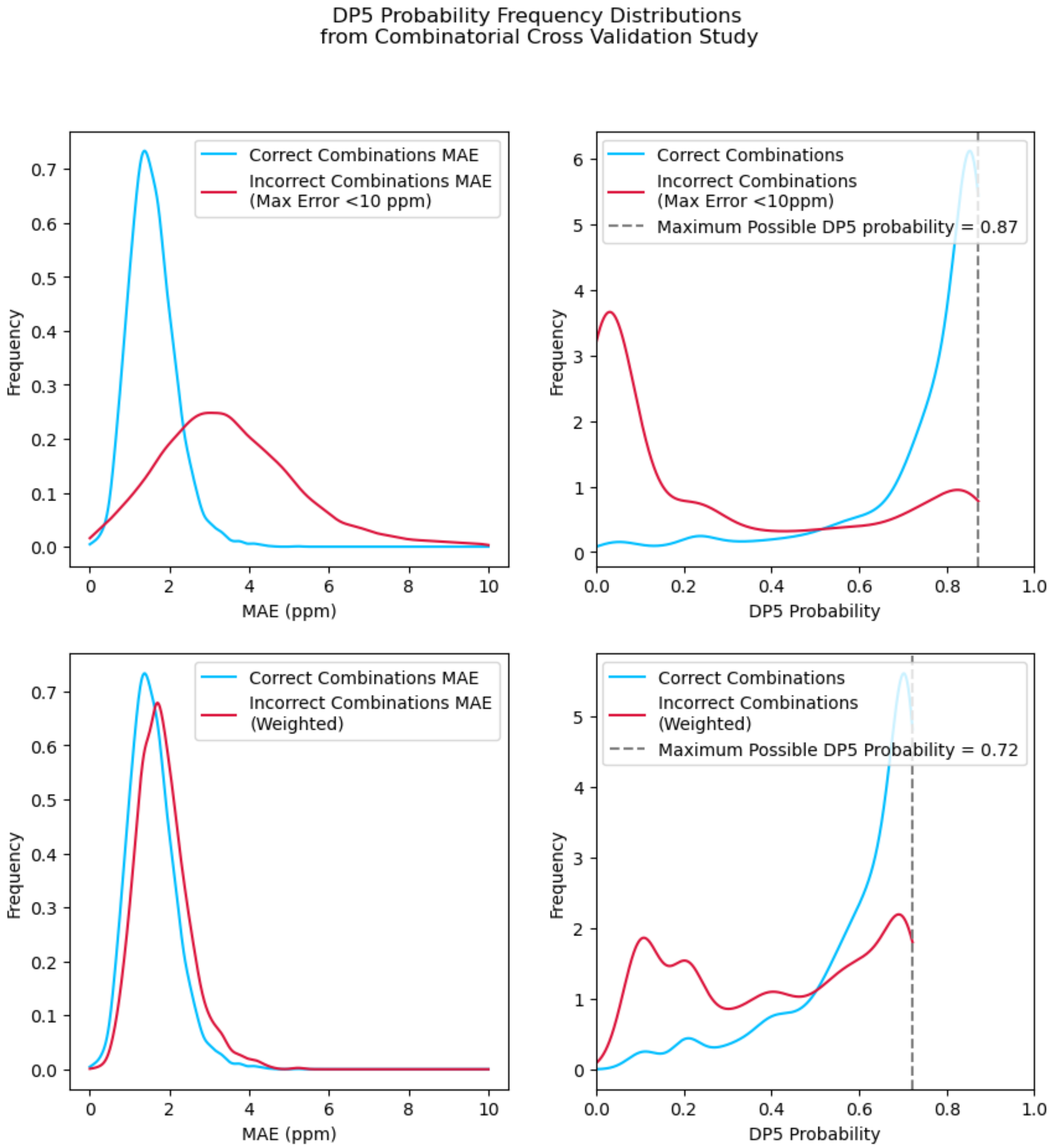

Figure 31: Figure displaying results from the combinatorial cross validation study. In this figure, the molecular probabilities were calculated using equation 8 atomic probabilities were found using equation 4 and the kernel sigma value was set to 0.025. Top right: the frequency distributions of the DP5 probabilities of the correct combinations (blue) and the incorrect combinations with maximum errors $>$ 10 ppm (red). Bottom left: the MAE error distribution of the correct combinations (blue) and the MAE error distribution of incorrect combinations weighted by the corresponding probabilities from the correct combinations MAE distribution (red). Bottom right: the frequency distributions of the DP5 probabilities of the correct combinations (blue) and weighted frequency distribution of the DP5 probabilities of the incorrect combinations weighted by the corresponding probabilities from the correct combinations MAE distribution (red).

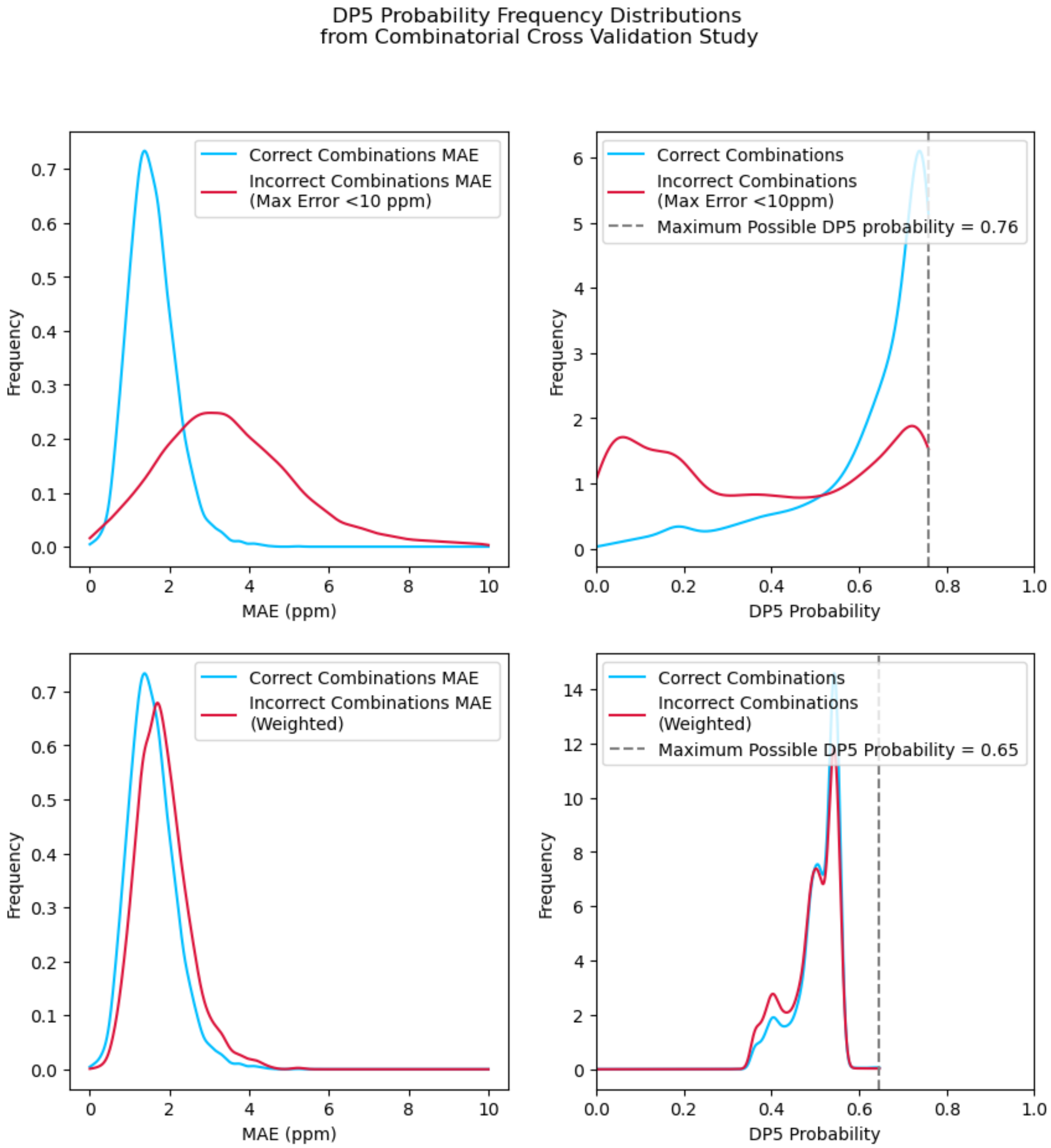

Figure 32: Figure displaying results from the combinatorial cross validation study. In this figure, the molecular probabilities were calculated using equation 7 atomic probabilities were found using equation 2 and the kernel sigma value was set to infinity. Top right: the frequency distributions of the DP5 probabilities of the correct combinations (blue) and the incorrect combinations with maximum errors $>$ 10 ppm (red). Bottom left: the MAE error distribution of the correct combinations (blue) and the MAE error distribution of incorrect combinations weighted by the corresponding probabilities from the correct combinations MAE distribution (red). Bottom right: the frequency distributions of the DP5 probabilities of the correct combinations (blue) and weighted frequency distribution of the DP5 probabilities of the incorrect combinations weighted by the corresponding probabilities from the correct combinations MAE distribution (red).

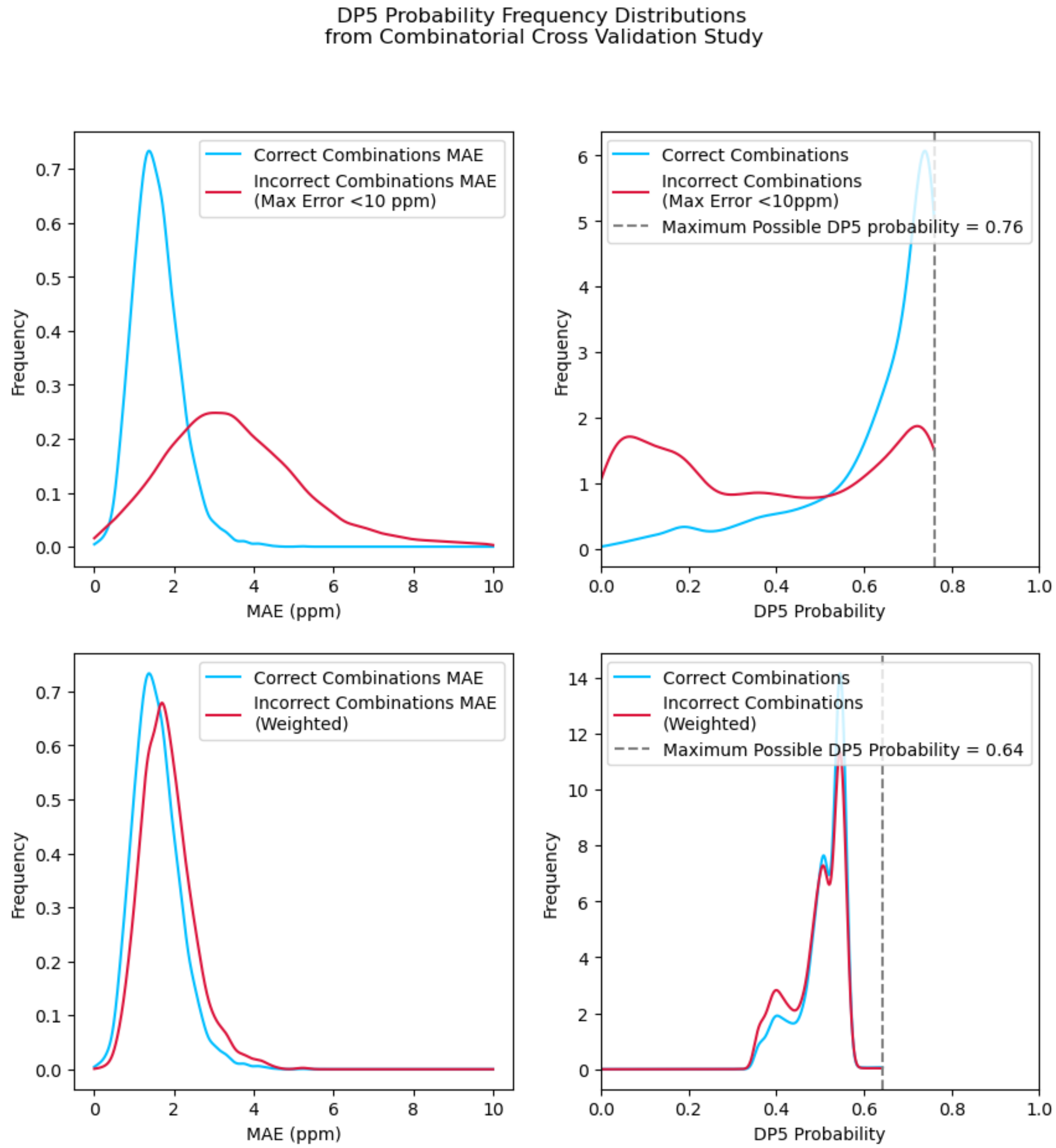

Figure 33: Figure displaying results from the combinatorial cross validation study. In this figure, the molecular probabilities were calculated using equation 7 atomic probabilities were found using equation 2 and the kernel sigma value was set to 0.3. Top right: the frequency distributions of the DP5 probabilities of the correct combinations (blue) and the incorrect combinations with maximum errors > 10 ppm (red). Bottom left: the MAE error distribution of the correct combinations (blue) and the MAE error distribution of incorrect combinations weighted by the corresponding probabilities from the correct combinations MAE distribution (red). Bottom right: the frequency distributions of the DP5 probabilities of the correct combinations (blue) and weighted frequency distribution of the DP5 probabilities of the incorrect combinations weighted by the corresponding probabilities from the correct combinations MAE distribution (red).

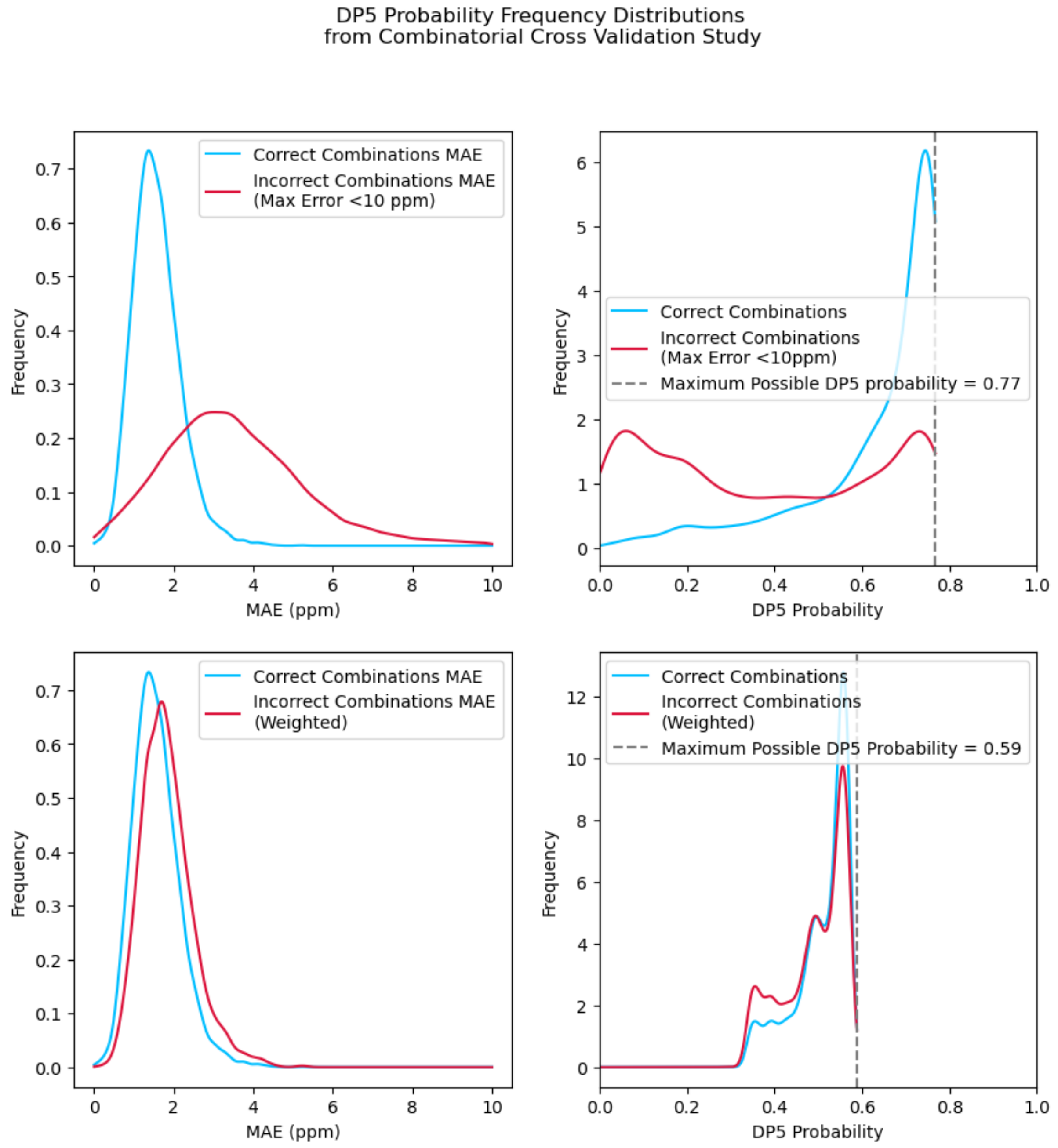

Figure 34: Figure displaying results from the combinatorial cross validation study. In this figure, the molecular probabilities were calculated using equation 7 atomic probabilities were found using equation 2 and the kernel sigma value was set to 0.1. Top right: the frequency distributions of the DP5 probabilities of the correct combinations (blue) and the incorrect combinations with maximum errors > 10 ppm (red). Bottom left: the MAE error distribution of the correct combinations (blue) and the MAE error distribution of incorrect combinations weighted by the corresponding probabilities from the correct combinations MAE distribution (red). Bottom right: the frequency distributions of the DP5 probabilities of the correct combinations (blue) and weighted frequency distribution of the DP5 probabilities of the incorrect combinations weighted by the corresponding probabilities from the correct combinations MAE distribution (red).

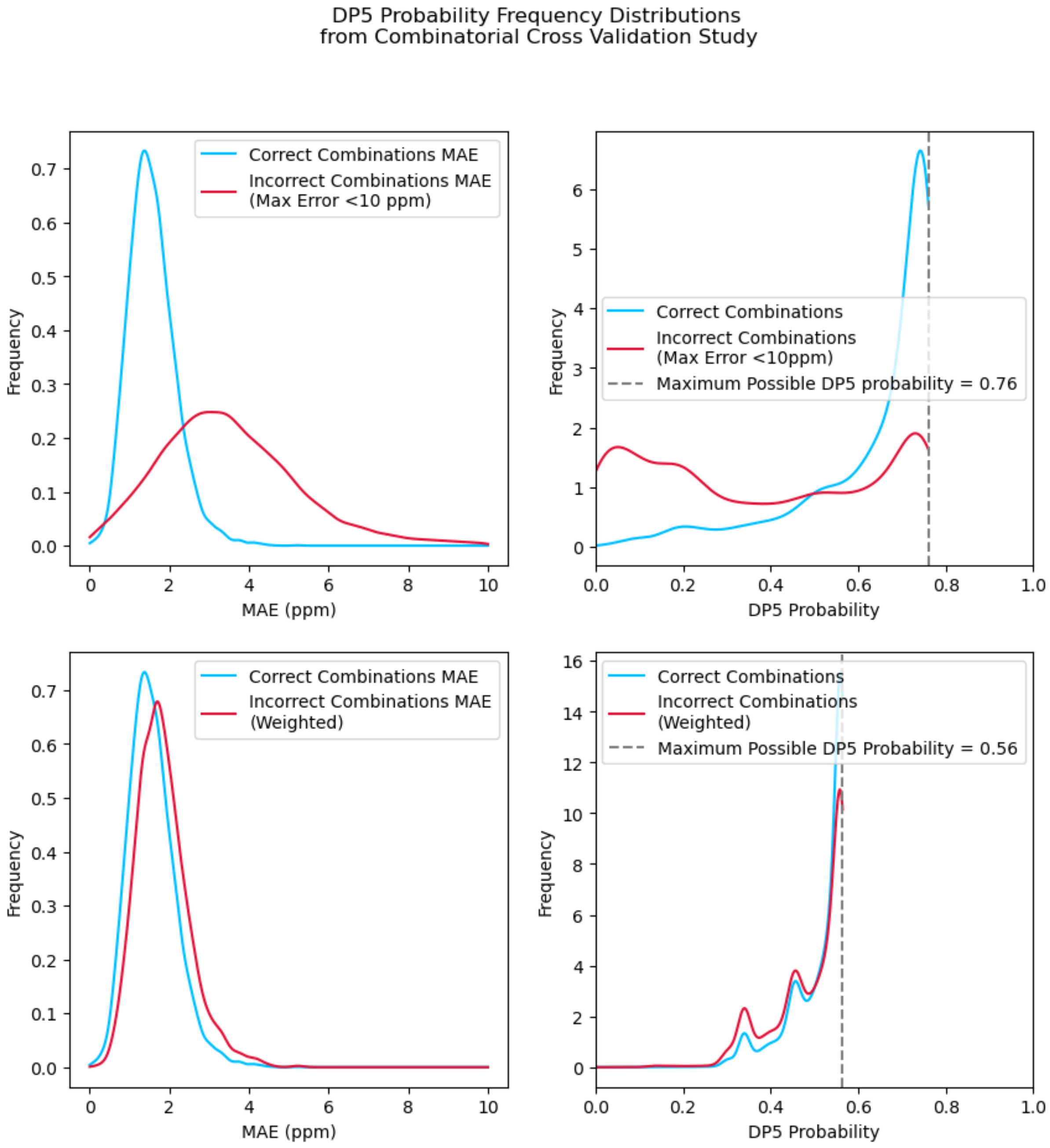

Figure 35: Figure displaying results from the combinatorial cross validation study. In this figure, the molecular probabilities were calculated using equation 7 atomic probabilities were found using equation 2 and the kernel sigma value was set to 0.025. Top right: the frequency distributions of the DP5 probabilities of the correct combinations (blue) and the incorrect combinations with maximum errors > 10 ppm (red). Bottom left: the MAE error distribution of the correct combinations (blue) and the MAE error distribution of incorrect combinations weighted by the corresponding probabilities from the correct combinations MAE distribution (red). Bottom right: the frequency distributions of the DP5 probabilities of the correct combinations (blue) and weighted frequency distribution of the DP5 probabilities of the incorrect combinations weighted by the corresponding probabilities from the correct combinations MAE distribution (red).

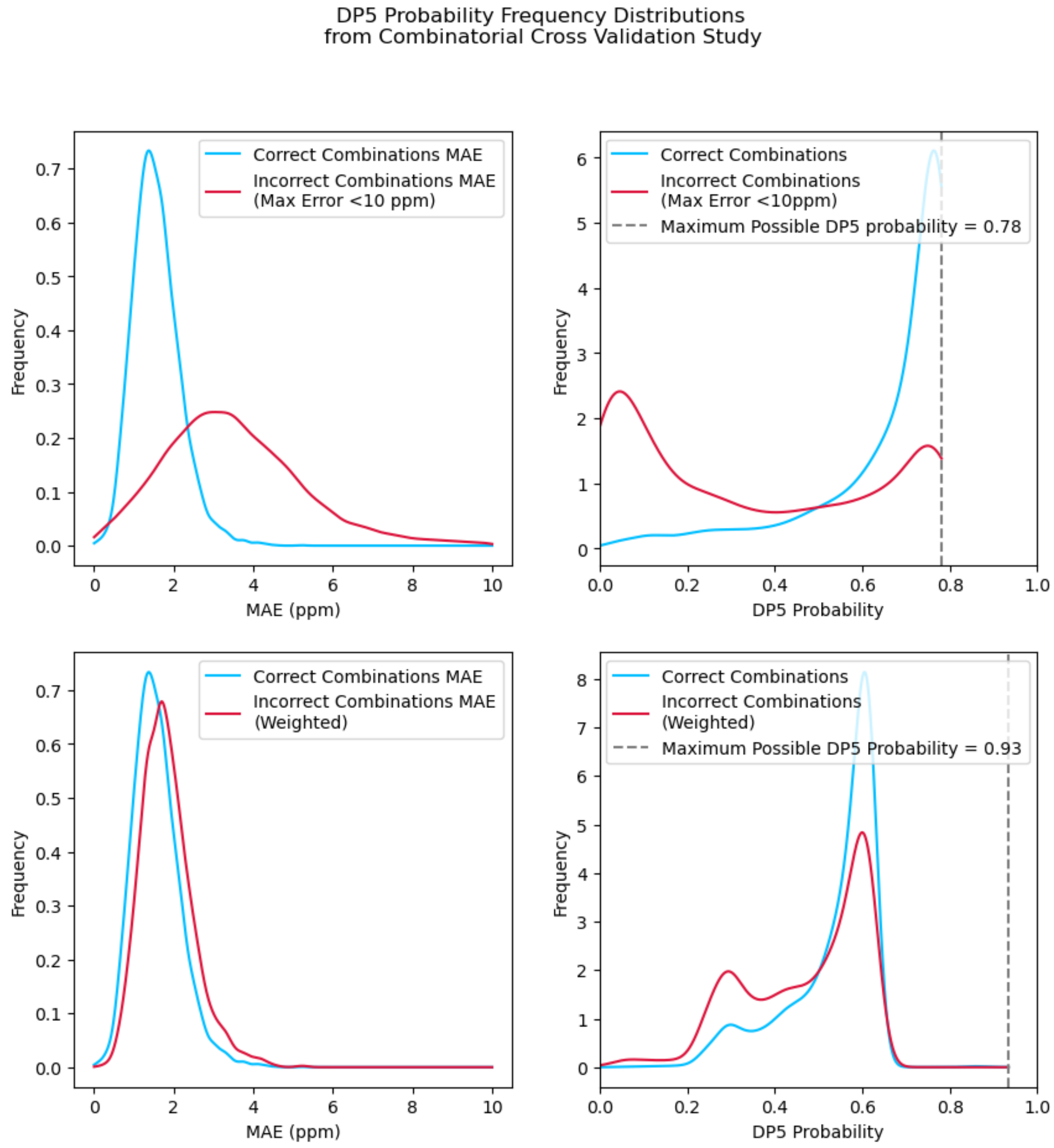

Figure 36: Figure displaying results from the combinatorial cross validation study. In this figure, the molecular probabilities were calculated using equation 7 atomic probabilities were found using equation 3 and the kernel sigma value was set to infinity. Top right: the frequency distributions of the DP5 probabilities of the correct combinations (blue) and the incorrect combinations with maximum errors > 10 ppm (red). Bottom left: the MAE error distribution of the correct combinations (blue) and the MAE error distribution of incorrect combinations weighted by the corresponding probabilities from the correct combinations MAE distribution (red). Bottom right: the frequency distributions of the DP5 probabilities of the correct combinations (blue) and weighted frequency distribution of the DP5 probabilities of the incorrect combinations weighted by the corresponding probabilities from the correct combinations MAE distribution (red).

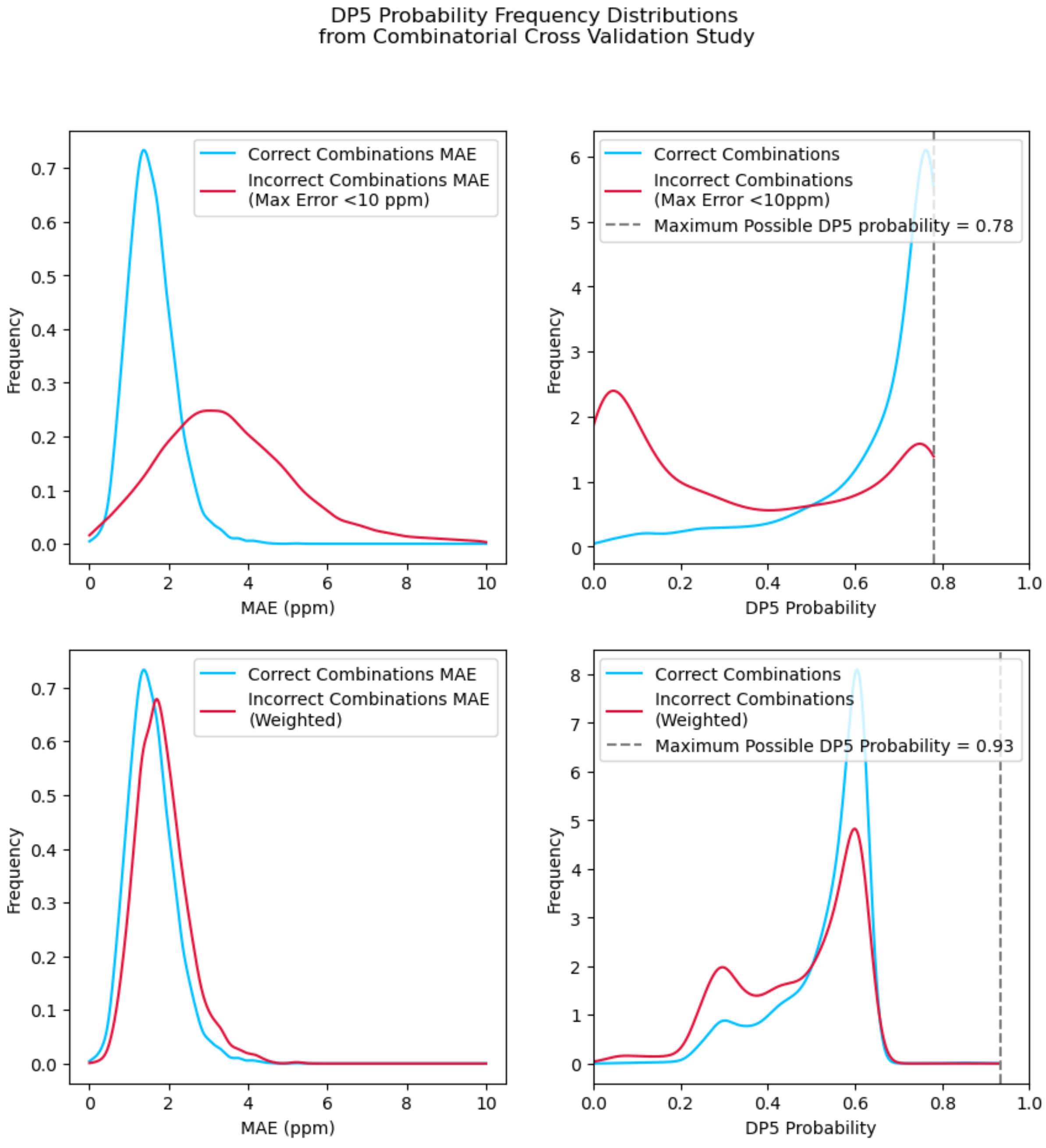

Figure 37: Figure displaying results from the combinatorial cross validation study. In this figure, the molecular probabilities were calculated using equation 7 atomic probabilities were found using equation 3 and the kernel sigma value was set to 0.3. Top right: the frequency distributions of the DP5 probabilities of the correct combinations (blue) and the incorrect combinations with maximum errors > 10 ppm (red). Bottom left: the MAE error distribution of the correct combinations (blue) and the MAE error distribution of incorrect combinations weighted by the corresponding probabilities from the correct combinations MAE distribution (red). Bottom right: the frequency distributions of the DP5 probabilities of the correct combinations (blue) and weighted frequency distribution of the DP5 probabilities of the incorrect combinations weighted by the corresponding probabilities from the correct combinations MAE distribution (red).

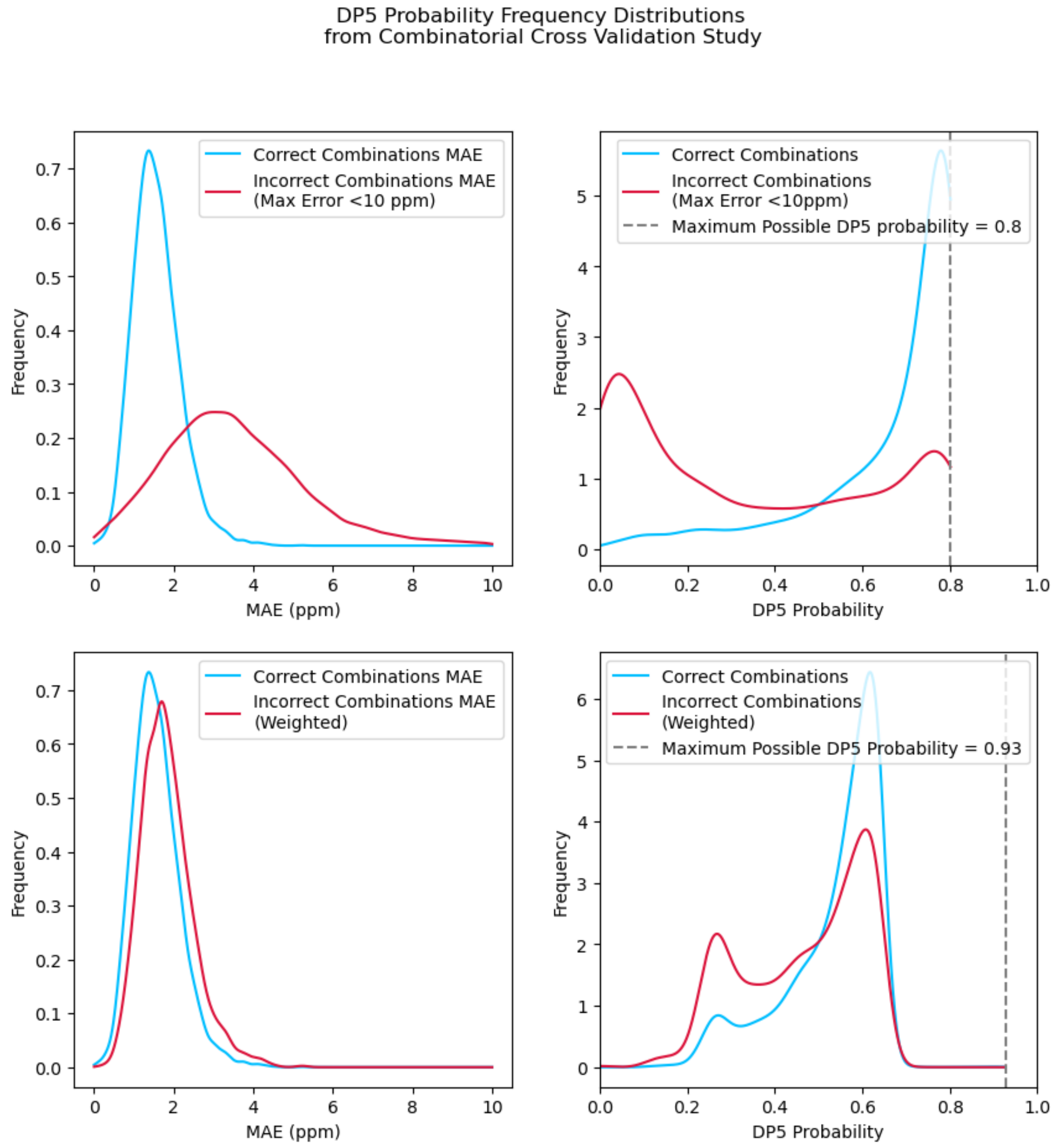

Figure 38: Figure displaying results from the combinatorial cross validation study. In this figure, the molecular probabilities were calculated using equation 7 atomic probabilities were found using equation 3 and the kernel sigma value was set to 0.1. Top right: the frequency distributions of the DP5 probabilities of the correct combinations (blue) and the incorrect combinations with maximum errors $>$ 10 ppm (red). Bottom left: the MAE error distribution of the correct combinations (blue) and the MAE error distribution of incorrect combinations weighted by the corresponding probabilities from the correct combinations MAE distribution (red). Bottom right: the frequency distributions of the DP5 probabilities of the correct combinations (blue) and weighted frequency distribution of the DP5 probabilities of the incorrect combinations weighted by the corresponding probabilities from the correct combinations MAE distribution (red).

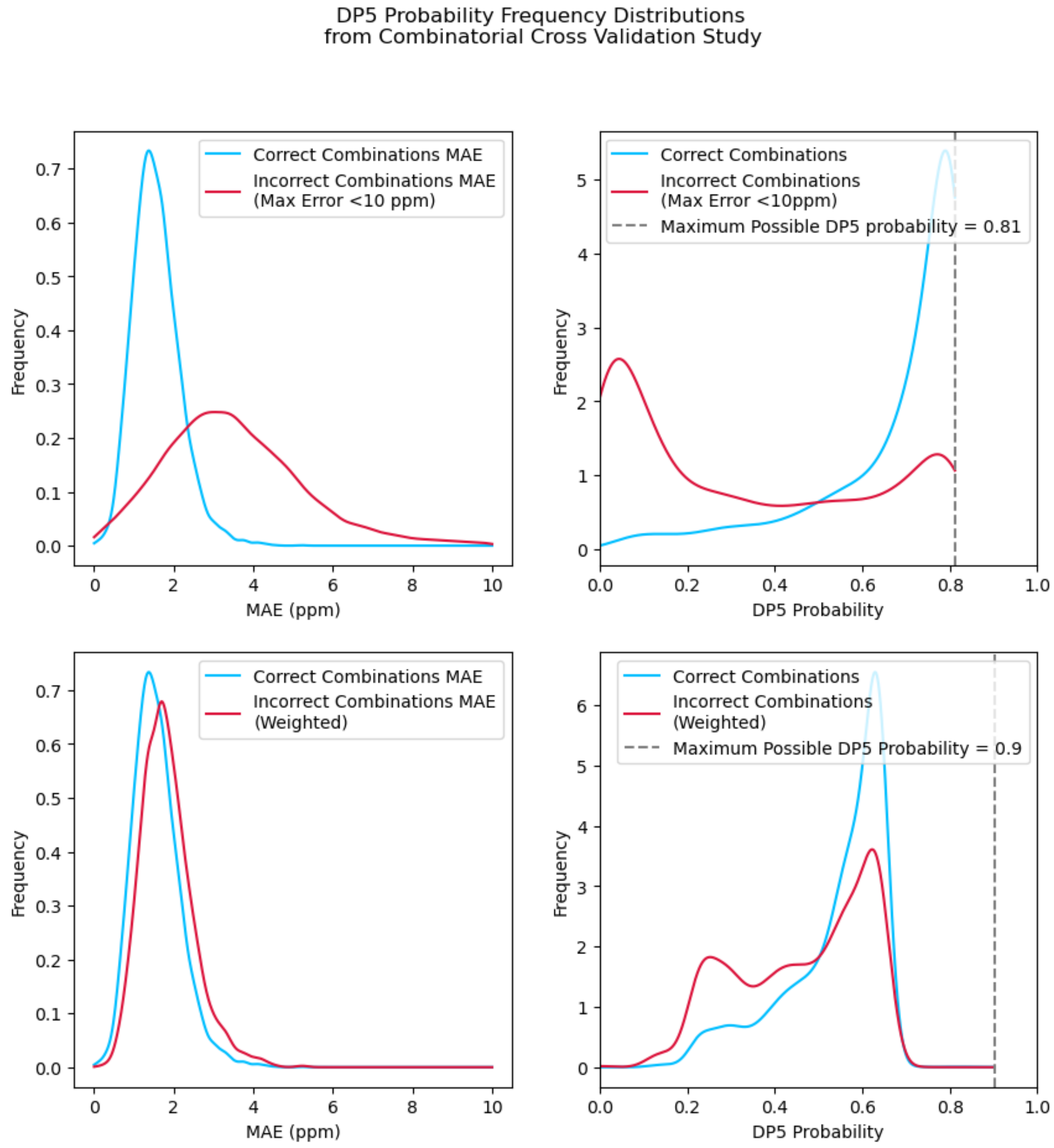

Figure 39: Figure displaying results from the combinatorial cross validation study. In this figure, the molecular probabilities were calculated using equation 7 atomic probabilities were found using equation 3 and the kernel sigma value was set to 0.075. Top right: the frequency distributions of the DP5 probabilities of the correct combinations (blue) and the incorrect combinations with maximum errors > 10 ppm (red). Bottom left: the MAE error distribution of the correct combinations (blue) and the MAE error distribution of incorrect combinations weighted by the corresponding probabilities from the correct combinations MAE distribution (red). Bottom right: the frequency distributions of the DP5 probabilities of the correct combinations (blue) and weighted frequency distribution of the DP5 probabilities of the incorrect combinations weighted by the corresponding probabilities from the correct combinations MAE distribution (red).

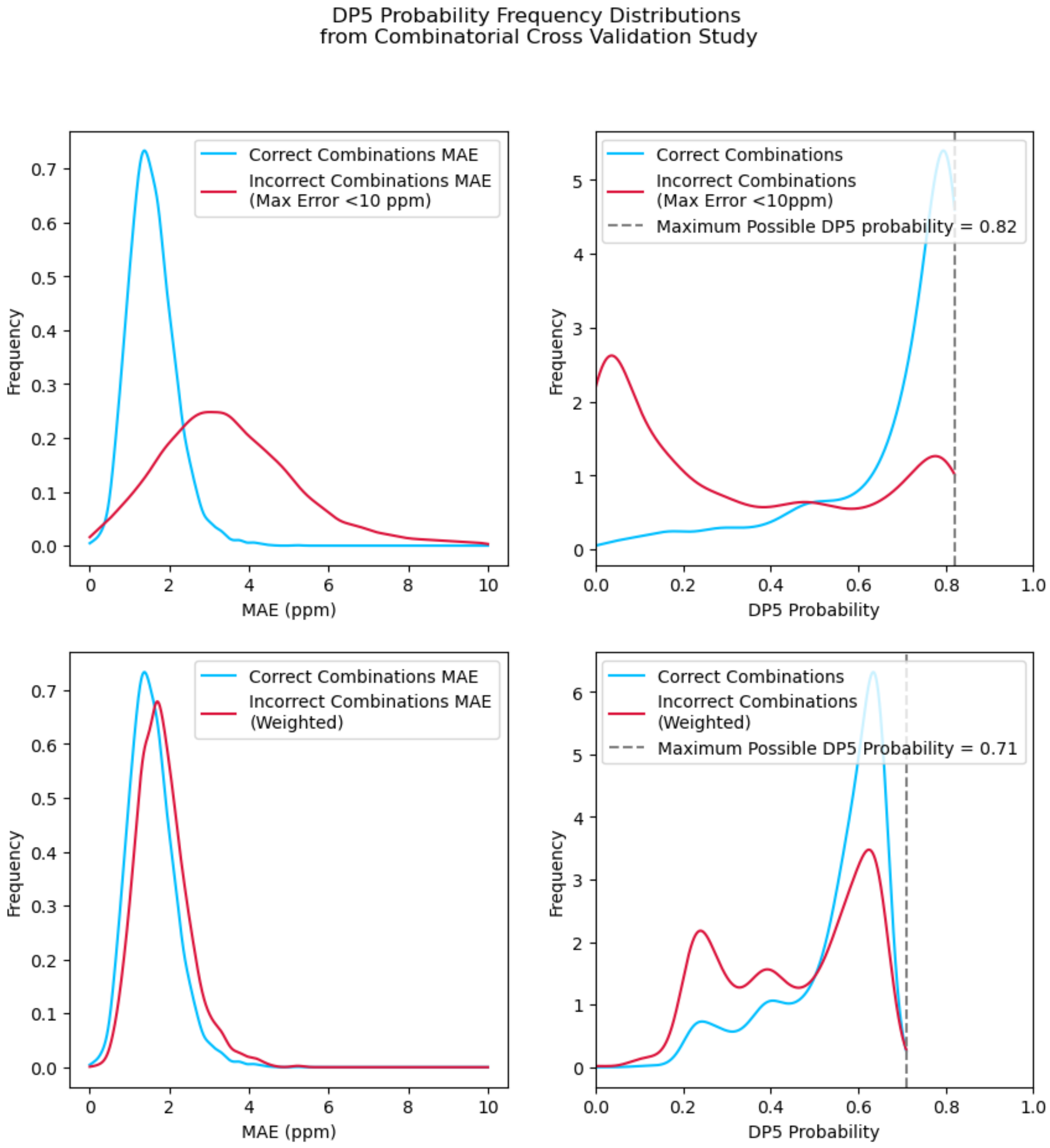

Figure 40: Figure displaying results from the combinatorial cross validation study. In this figure, the molecular probabilities were calculated using equation 7 atomic probabilities were found using equation 3 and the kernel sigma value was set to 0.05. Top right: the frequency distributions of the DP5 probabilities of the correct combinations (blue) and the incorrect combinations with maximum errors $>$ 10 ppm (red). Bottom left: the MAE error distribution of the correct combinations (blue) and the MAE error distribution of incorrect combinations weighted by the corresponding probabilities from the correct combinations MAE distribution (red). Bottom right: the frequency distributions of the DP5 probabilities of the correct combinations (blue) and weighted frequency distribution of the DP5 probabilities of the incorrect combinations weighted by the corresponding probabilities from the correct combinations MAE distribution (red).

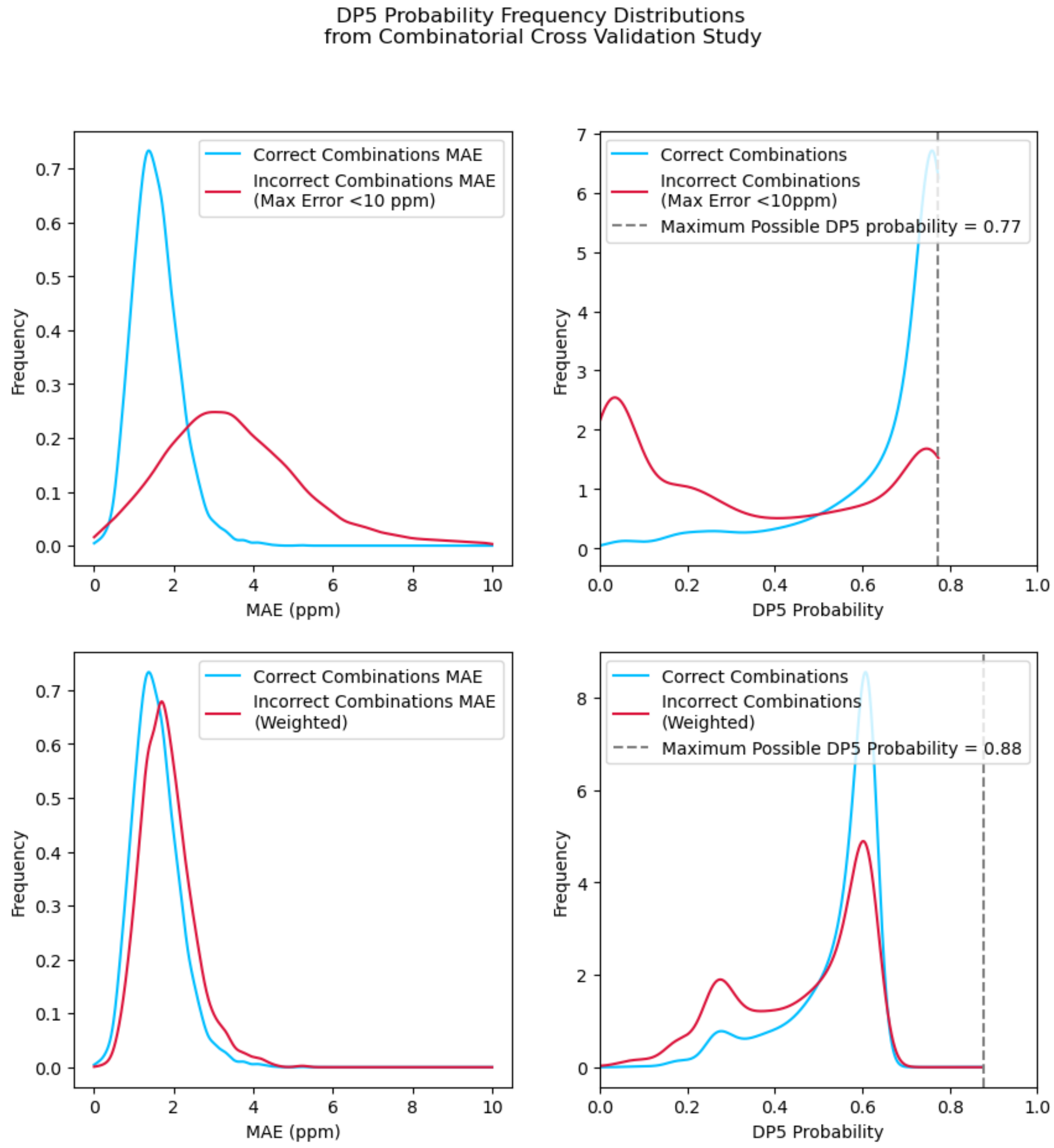

Figure 41: Figure displaying results from the combinatorial cross validation study. In this figure, the molecular probabilities were calculated using equation 7 atomic probabilities were found using equation 3 and the kernel sigma value was set to 0.025. Top right: the frequency distributions of the DP5 probabilities of the correct combinations (blue) and the incorrect combinations with maximum errors $>$ 10 ppm (red). Bottom left: the MAE error distribution of the correct combinations (blue) and the MAE error distribution of incorrect combinations weighted by the corresponding probabilities from the correct combinations MAE distribution (red). Bottom right: the frequency distributions of the DP5 probabilities of the correct combinations (blue) and weighted frequency distribution of the DP5 probabilities of the incorrect combinations weighted by the corresponding probabilities from the correct combinations MAE distribution (red).

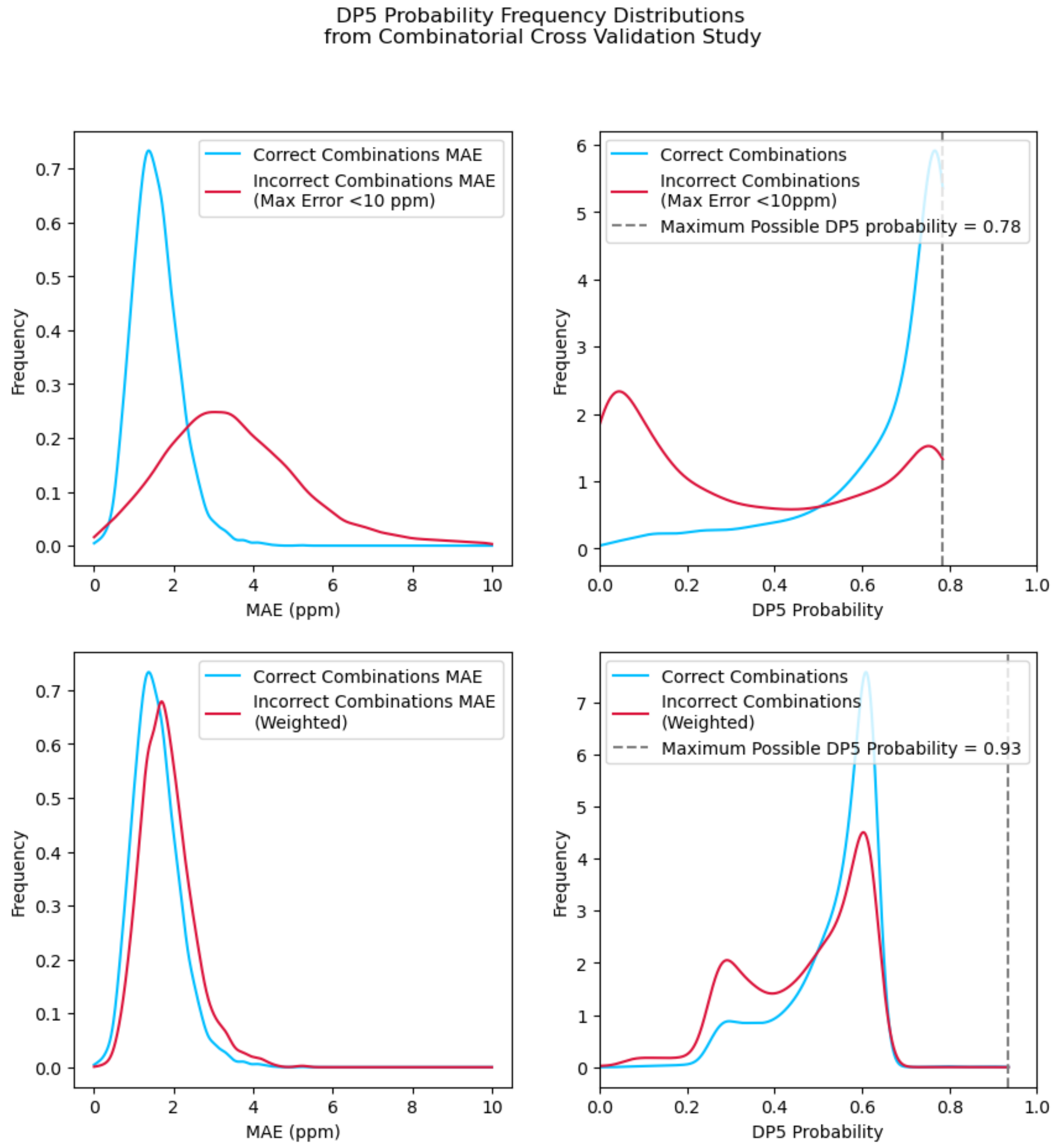

Figure 42: Figure displaying results from the combinatorial cross validation study. In this figure, the molecular probabilities were calculated using equation 7 atomic probabilities were found using equation 4 and the kernel sigma value was set to 0.3. Top right: the frequency distributions of the DP5 probabilities of the correct combinations (blue) and the incorrect combinations with maximum errors > 10 ppm (red). Bottom left: the MAE error distribution of the correct combinations (blue) and the MAE error distribution of incorrect combinations weighted by the corresponding probabilities from the correct combinations MAE distribution (red). Bottom right: the frequency distributions of the DP5 probabilities of the correct combinations (blue) and weighted frequency distribution of the DP5 probabilities of the incorrect combinations weighted by the corresponding probabilities from the correct combinations MAE distribution (red).

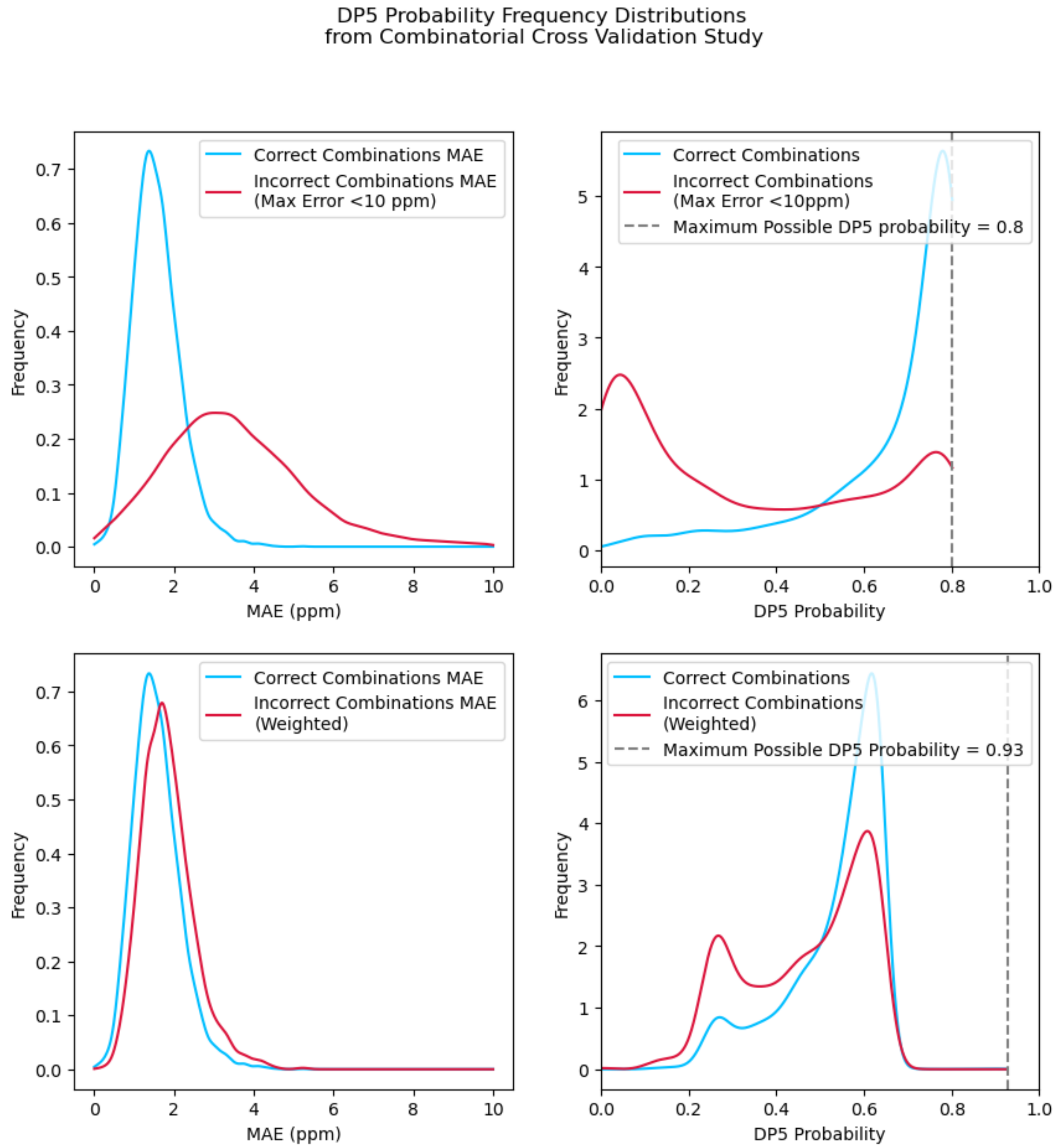

Figure 43: Figure displaying results from the combinatorial cross validation study. In this figure, the molecular probabilities were calculated using equation 7 atomic probabilities were found using equation 4 and the kernel sigma value was set to 0.1. Top right: the frequency distributions of the DP5 probabilities of the correct combinations (blue) and the incorrect combinations with maximum errors > 10 ppm (red). Bottom left: the MAE error distribution of the correct combinations (blue) and the MAE error distribution of incorrect combinations weighted by the corresponding probabilities from the correct combinations MAE distribution (red). Bottom right: the frequency distributions of the DP5 probabilities of the correct combinations (blue) and weighted frequency distribution of the DP5 probabilities of the incorrect combinations weighted by the corresponding probabilities from the correct combinations MAE distribution (red).

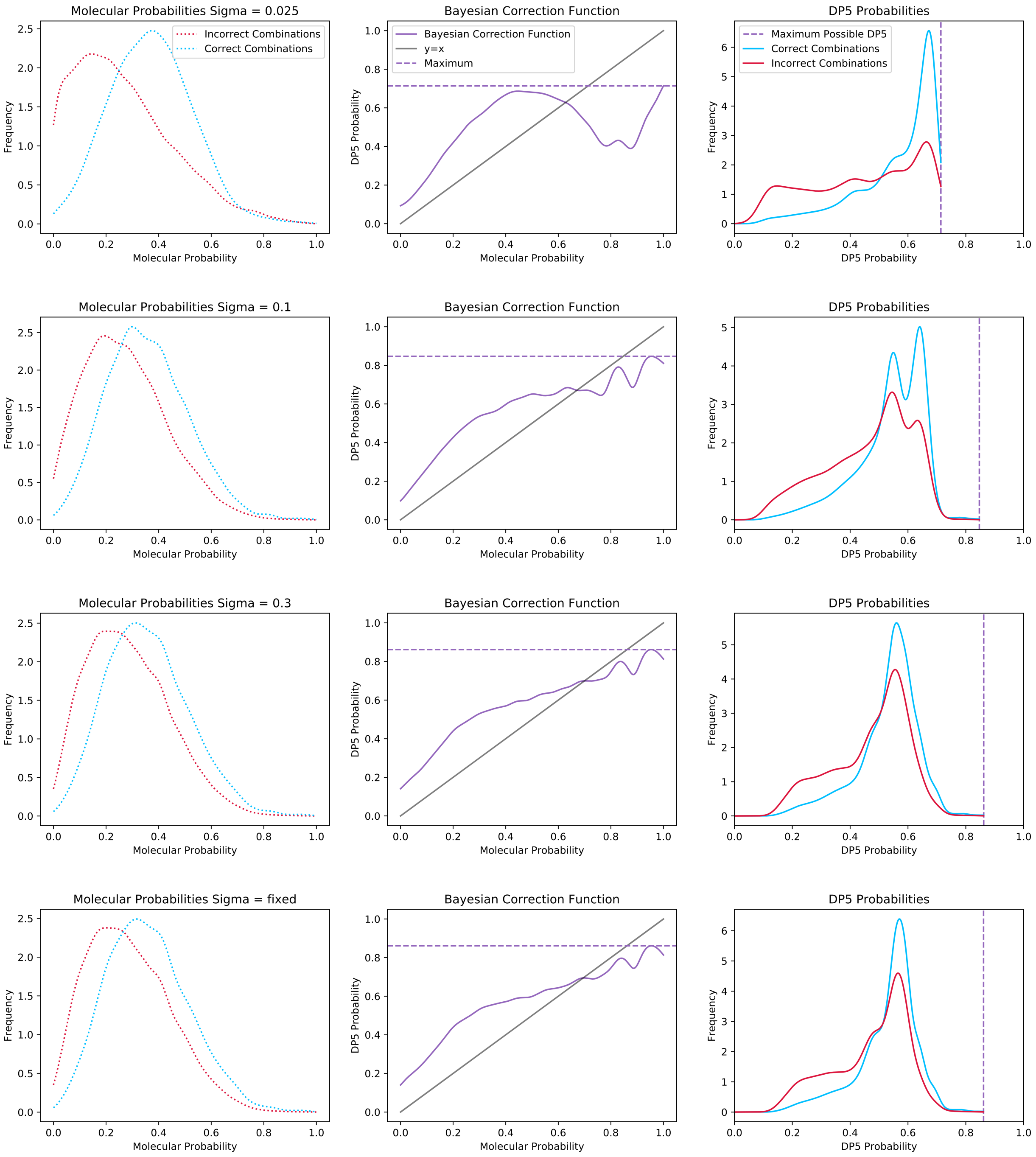

Figure 44: Figure comparing results from cross validation study for formulations of the DP5 probability differing only by the choice of sigma parameter. Molecular probabilities were calculated using equation 8 and the atomic probabilities were found using equation 2. Left: frequency distributions of molecular probabilities assigned to the correct (blue) and incorrect (red) combinations, where the incorrect combinations have been weighted such that their resulting MAE distribution matches that of the correct combinations. Centre: the bayesian correction function applied. Right: frequency distributions of final DP5 probabilities for correct (blue) and weighted incorrect combinations (red).

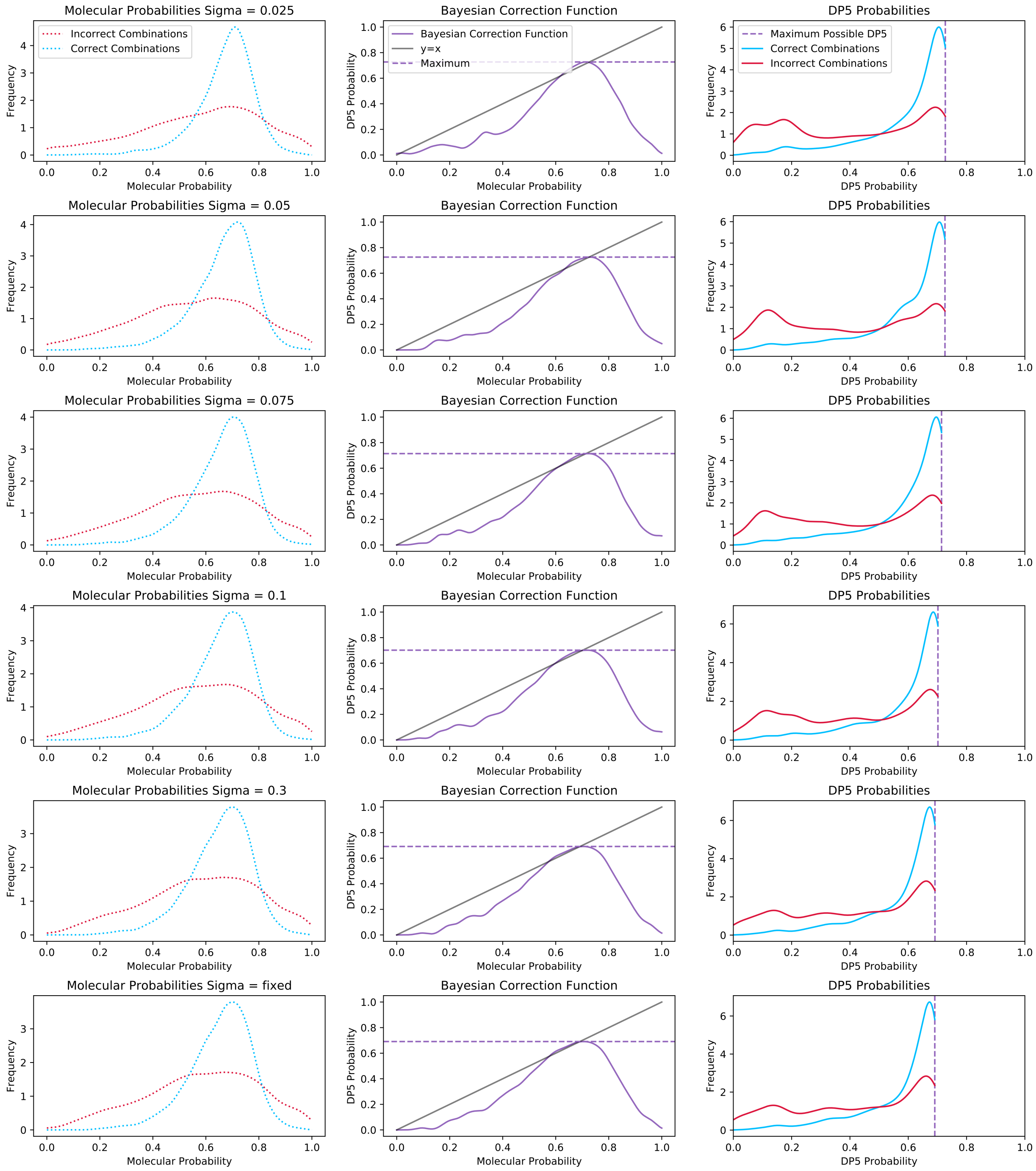

Figure 45: Figure comparing results from cross validation study for formulations of the DP5 probability differing only by the choice of sigma parameter. Molecular probabilities were calculated using equation 8 and the atomic probabilities were found using equation 3. Left: frequency distributions of molecular probabilities assigned to the correct (blue) and incorrect (red) combinations, where the incorrect combinations have been weighted such that their resulting MAE distribution matches that of the correct combinations. Centre: the bayesian correction function applied. Right: frequency distributions of final DP5 probabilities for correct (blue) and weighted incorrect combinations (red).

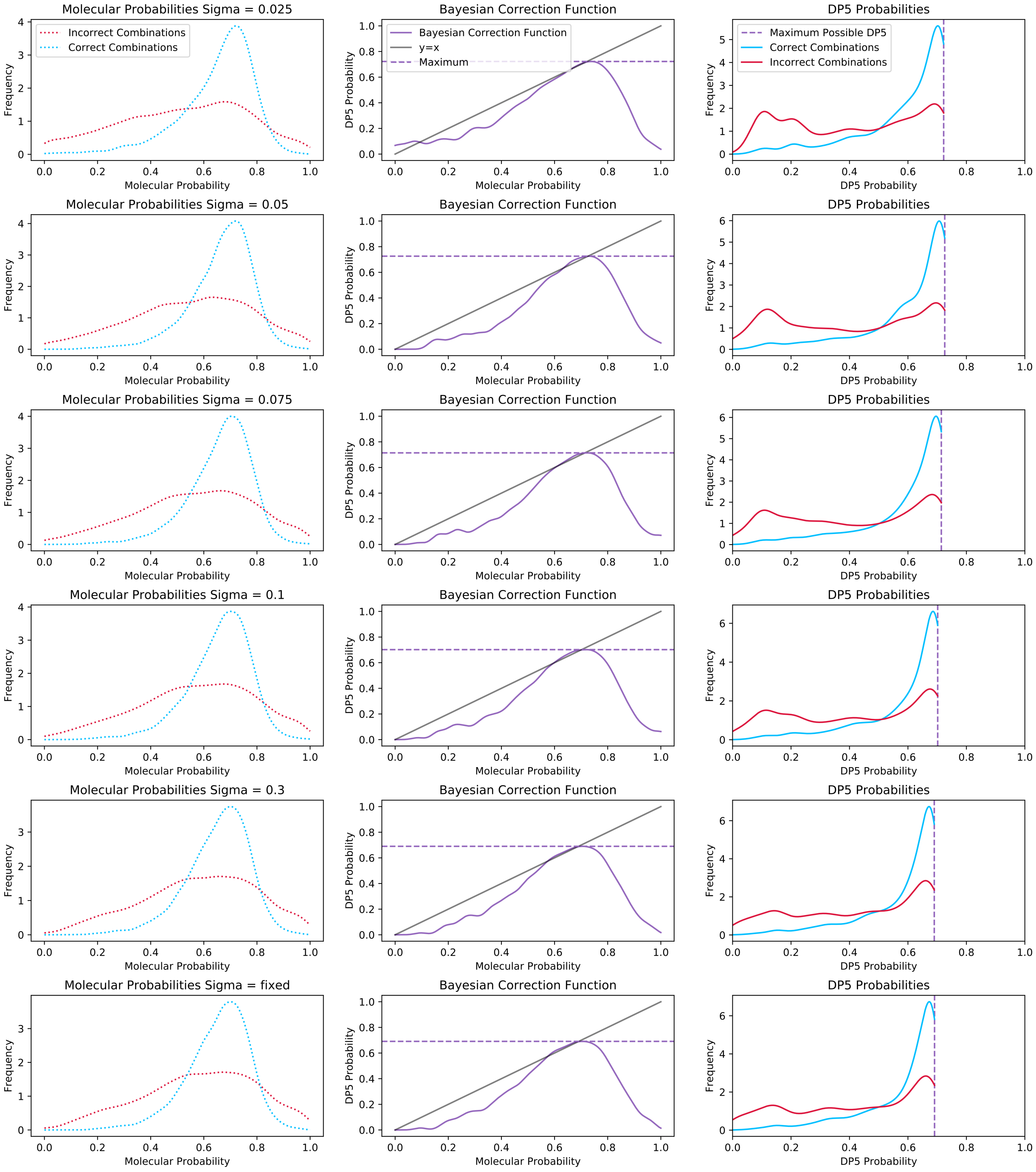

Figure 46: Figure comparing results from cross validation study for formulations of the DP5 probability differing only by the choice of sigma parameter. Molecular probabilities were calculated using equation 8 and the atomic probabilities were found using equation 4. Left: frequency distributions of molecular probabilities assigned to the correct (blue) and incorrect (red) combinations, where the incorrect combinations have been weighted such that their resulting MAE distribution matches that of the correct combinations. Centre: the bayesian correction function applied. Right: frequency distributions of final DP5 probabilities for correct (blue) and weighted incorrect combinations (red).

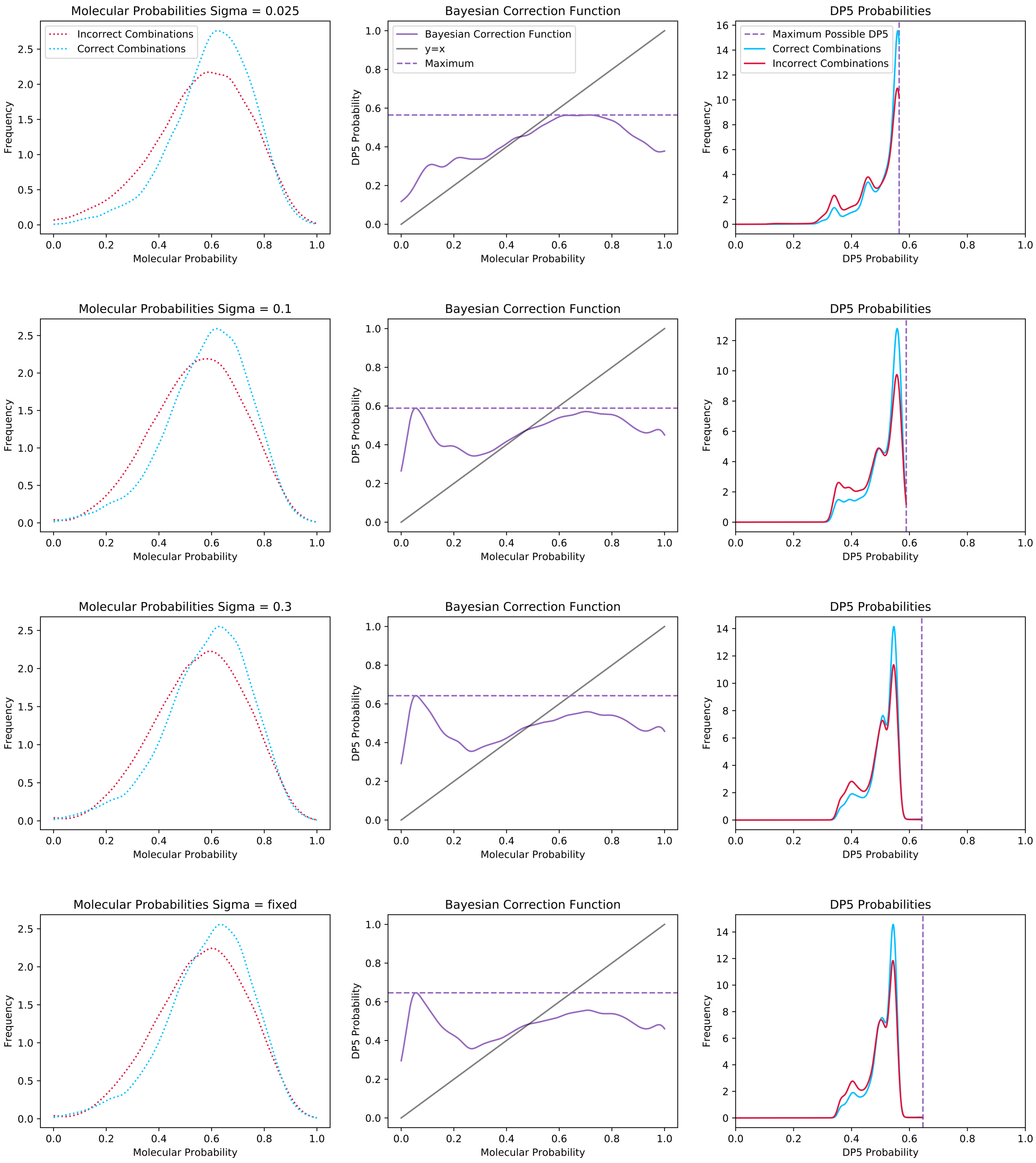

Figure 47: Figure comparing results from cross validation study for formulations of the DP5 probability differing only by the choice of sigma parameter. Molecular probabilities were calculated using equation 7 and the atomic probabilities were found using equation 2. Left: frequency distributions of molecular probabilities assigned to the correct (blue) and incorrect (red) combinations, where the incorrect combinations have been weighted such that their resulting MAE distribution matches that of the correct combinations. Centre: the bayesian correction function applied. Right: frequency distributions of final DP5 probabilities for correct (blue) and weighted incorrect combinations (red).

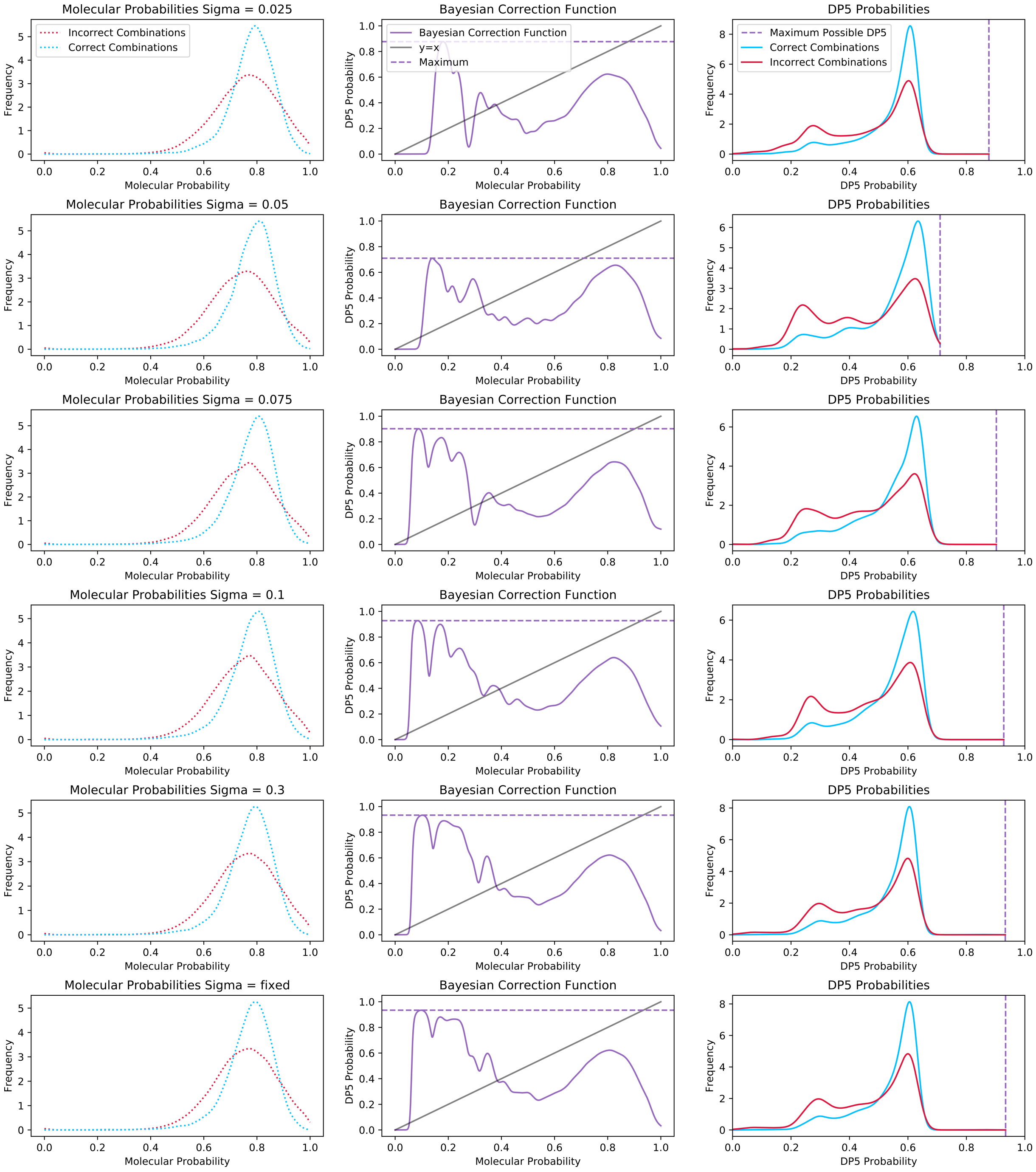

Figure 48: Figure comparing results from cross validation study for formulations of the DP5 probability differing only by the choice of sigma parameter. Molecular probabilities were calculated using equation 7 and the atomic probabilities were found using equation 3. Left: frequency distributions of molecular probabilities assigned to the correct (blue) and incorrect (red) combinations, where the incorrect combinations have been weighted such that their resulting MAE distribution matches that of the correct combinations. Centre: the bayesian correction function applied. Right: frequency distributions of final DP5 probabilities for correct (blue) and weighted incorrect combinations (red).

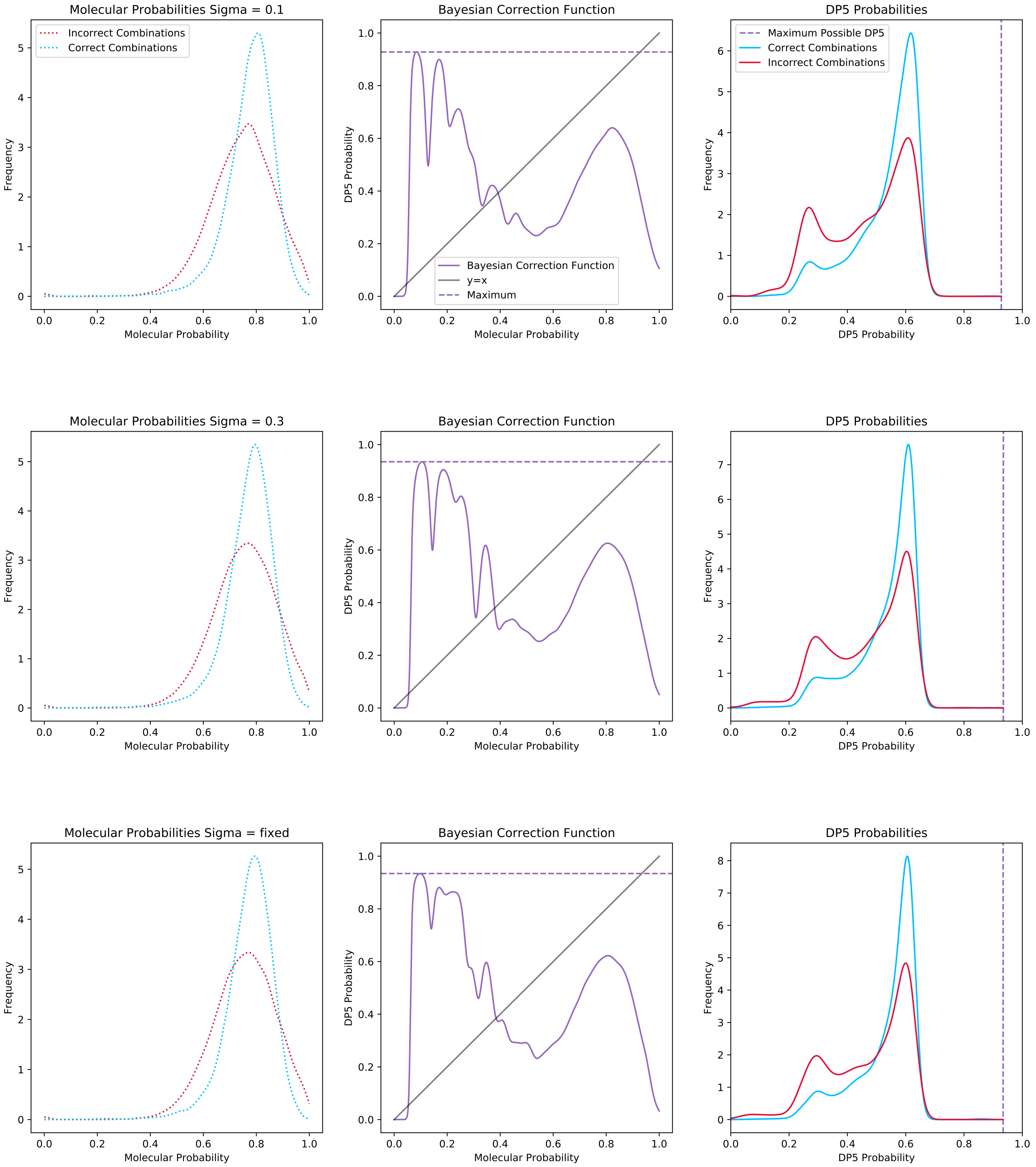

Figure 49: Figure comparing results from cross validation study for formulations of the DP5 probability differing only by the choice of sigma parameter. Molecular probabilities were calculated using equation 7 and the atomic probabilities were found using equation 4. Left: frequency distributions of molecular probabilities assigned to the correct (blue) and incorrect (red) combinations, where the incorrect combinations have been weighted such that their resulting MAE distribution matches that of the correct combinations. Centre: the bayesian correction function applied. Right: frequency distributions of final DP5 probabilities for correct (blue) and weighted incorrect combinations (red).

6 NMRShiftDB Molecules

NMRShiftDB IDs for the 5140 molecules used in DP5s internal dataset and in the combinatorial studies. This dataset was originally constructed by Paton et al.[18]

2192 2194 2198 2202 2203 2207 2230 2241 2245 2248 2251 2255 2276 2279 2282 2290 2293 2339 2340
2348 2361 2366 2370 2381 2425 2431 2432 2440 2443 2452 2453 2469 2481 2495 2506 2529 2537 2553
2558 2559 2568 2573 2581 2584 2585 2590 2593 2595 2596 2600 2610 2693 2697 2735 2739 2745 2782
2820 2825 2861 2862 3156 3285 3292 3325 3408 3409 3492 3707 3715 3744 3870 3873 3995 4206 4289
4293 4295 4420 5508 6605 6609 6768 7061 7066 7078 7108 7229 7236 7359 7489 7902 7912 7986 7992 8411
9670 9671 9754 10088 10258 10505 10592 10886 11012 11093 11220 11224 11225 11226 11305 11348 11352
11683 17453 19748 19831 19834 19872 19875 19918 19998 20001 20043 20082 20140 20334 21070 21111
21115 21125 21156 21326 21333 21786 22047 22127 22129 22130 22147 22169 74182 74209 74215 74217
74220 74224 74233 74250 74258 74260 74262 74272 74274 74275 74276 74277 74278 74279 74280 74283
74287 74291 74297 74934 74935 74937 74938 74939 74941 74945 74947 74950 74952 74955 74963 74967
74974 74975 74998 75003 75012 75013 75018 75019 75028 75040 75043 75044 75048 75052 75054 75058
75290 75293 75299 75301 75305 75310 75316 75319 75320 75321 75324 75327 75328 75329 75336 75344
75347 75349 75362 75363 75367 75383 75386 75388 75391 75399 75403 75404 75408 75413 75419 75425
75426 75427 75664 75668 75669 75686 75690 75694 75704 75706 75711 75712 75717 75718 75728 75744
75746 75749 75754 75762 75773 75775 75782 75784 75786 75787 75788 75789 76040 76054 76060 76063
76071 76072 76073 76076 76077 76089 76092 76132 76133 76164 76410 76435 76440 76441 76442 76448
76464 76478 76482 76484 76485 76487 76490 76491 76494 76495 76498 76505 76518 76523 76544 76549
76551 76552 76557 76786 76794 76797 76803 76806 76816 76818 76819 76834 76840 76843 76844 76845
76880 76886 76890 76902 76903 76905 76910 76912 77158 77169 77170 77182 77196 77197 77201 77202
77205 77206 77208 77213 77223 77230 77231 77234 77235 77236 77237 77238 77241 77242 77245 77251
77253 77255 77256 77258 77259 77260 77272 77274 77281 77295 77298 77301 88191 88199 88391 88395
88396 88399 88400 88401 88407 88408 88409 88418 88426 88432 88441 88450 88454 88460 88462 88464
88465 88466 88470 88471 88472 88477 88483 88486 88492 88496 88752 88753 88767 88775 88776 88777
88778 88779 88783 88790 88800 88809 88815 88816 88824 88826 88837 88852 88853 88861 88874 88883
89126 89127 89144 89152 89155 89166 89170 89172 89181 89182 89192 89193 89194 89195 89200 89201
89207 89208 89213 89214 89216 89218 89220 89226 89227 89248 89250 89251 89252 89256 89258 89265
89266 89267 89541 89548 89549 89569 89573 89576 89580 89581 89588 89591 89602 89604 89620 89625
89628 89641 89642 89646 89879 89880 89884 89886 89889 89897 89904 89907 89915 89916 89917 89918
89920 89936 89937 89941 89946 89964 89966 89976 89977 89984 89995 90003 90004 90005 90248 90263
90264 90265 90270 90271 90272 90274 90278 90279 90283 90285 90287 90289 90300 90306 90313 90318
90322 90323 90336 90337 90351 90357 90362 90369 90370 90378 90380 90381 90386 90632 90636 90644
90646 90648 90652 90674 90680 90694 90699 90700 90701 90719 90729 90733 10003368 10003451 10003533
10003578 10005613 10005727 10005795 10005811 10005853 10005985 10006107 10006277 10006293 10006294
10006295 10006315 10006316 10006345 10006644 10006977 10006982 10007145 10007149 10007150 10007649
10007655 10007667 10007825 10008070 10008157 10008330 10008574 10008576 10008584 10008597 10008628
10008629 10008643 10008729 10008735 10008744 10008771 10008778 10008835 10008849 10008882 10008898
10008929 10008940 10008981 10008986 10008987 10009012 10009015 10009028 10009029 10009035 10009037
10009040 10009064 10009071 10009072 10009075 10009077 10009080 10009088 10009089 10009091 10009093
10009095 10009102 10009113 10009115 10009117 10009120 10009122 10009123 10009124 10009126 10009155
10009156 10009159 10009164 10009207 10009230 10009253 10009254 10009255 10009259 10009269 10009270
10009273 10009278 10009279 10009281 10009285 10009287 10009292 10009295 10009297 10009298 10009337
10009836 10010005 10010379 10010421 10010426 10010467 10010850 10013804 10014033 10014077 10014078
10014081 10014118 10014119 10015650 10015823 10015862 10015866 10015869 10015890 10015899 10015915
10015919 10015922 10015923 10015925 10015926 10015927 10015937 10015941 10015976 10016002 10016035
10016123 10016287 10016297 10016308 10016311 10016364 10016425 10016428 10016430 10016556 10016575
10016628 10016638 10016649 10016656 10016661 10016662 10016664 10016673 10016679 10016690 10016691
10016694 10016700 10016701 10016703 10016706 10016710 10016712 10016715 10016722 10016725 10016727
10016728 10016729 10016730 10016732 10016734 10016758 10016773 10016774 10016795 10016798 10016802
10016828 10016843 10016866 10016880 10016887 10016888 10016894 10016895 10016898 10016902 10016921
10016924 10016936 10016941 10016946 10016950 10016953 10016966 10016967 10016972 10016975 10016978
10016979 10016982 10016990 10017001 10017013 10017017 10017018 10017024 10017032 10017035 10017038
10017041 10017043 10017104 10017144 10017393 10017480 10017590 10017593 10017596 10017603 10017612
10017620 10017629 10017639 10017665 10017670 10017674 10017686 10017695 10017704 10017729 10017731

10017740 10017750 10017753 10017759 10017761 10017766 10017767 10017781 10017783 10017792 10017793
10017794 10017795 10017803 10017804 10017808 10017825 10017835 10017842 10017843 10017844 10017855
10017857 10017859 10017867 10017871 10017872 10017874 10017875 10017876 10017883 10017887 10017889
10017890 10017892 10017893 10017899 10017912 10017916 10017930 10017931 10017932 10017942 10017948
10017949 10017950 10017960 10017961 10017969 10017988 10017990 10018006 10018008 10018011 10018020
10018026 10018040 10018069 10018071 10018089 10018090 10018091 10018092 10018100 10018112 10018117
10018118 10018119 10018126 10018138 10018140 10018143 10018151 10018156 10018159 10018160 10018166
10018167 10018171 10018180 10018181 10018186 10018188 10018194 10018195 10018204 10018223 10018225
10018228 10018230 10018231 10018236 10018244 10018248 10018278 10018301 10018302 10018306 10018307
10018310 10018319 10018321 10018324 10018340 10018342 10018343 10018350 10018355 10018360 10018361
10018363 10018364 10018371 10018393 10018394 10018397 10018399 10018404 10018414 10018418 10018421
10018424 10018436 10018437 10018443 10018449 10018452 10018453 10018456 10018460 10018469 10018476
10018487 10018491 10018494 10018497 10018502 10018510 10018512 10018532 10018533 10018534 10018537
10018538 10018543 10018544 10018551 10018555 10018557 10018558 10018564 10018565 10018566 10018569
10018573 10018574 10018576 10018578 10018580 10018581 10018593 10018607 10018617 10018619 10018648
10018653 10018655 10018678 10018681 10018683 10018684 10018703 10018710 10018716 10018720 10018723
10018725 10018727 10018731 10018743 10018745 10018746 10018748 10018749 10018750 10018762 10018790
10018800 10018805 10018809 10018810 10018812 10018815 10018827 10018830 10018831 10018861 10018871
10018879 10018880 10018885 10018891 10018896 10018900 10018909 10018910 10018913 10018915 10018926
10018927 10018943 10018944 10018948 10018950 10018951 10018959 10018963 10018965 10018974 10018975
10018980 10018982 10018987 10018991 10018995 10018997 10019000 10019005 10019013 10019014 10019017
10019019 10019031 10019032 10019033 10019046 10019063 10019064 10019066 10019071 10019078 10019080
10019088 10019092 10019101 10019102 10019103 10019111 10019120 10019126 10019130 10019134 10019140
10019143 10019152 10019153 10019179 10019184 10019186 10019193 10019199 10019210 10019219 10019228
10019230 10019233 10019247 10019248 10019250 10019261 10019266 10019273 10019283 10019293 10019294
10019298 10019299 10019300 10019304 10019306 10019311 10019312 10019316 10019317 10019318 10019336
10019339 10019341 10019347 10019354 10019362 10019363 10019371 10019374 10019377 10019385 10019392
10019402 10019406 10019411 10019454 10019495 10019497 10019536 10019704 10019871 10019956 10020482
10020483 10020522 10020564 10020694 10020902 10020904 10021219 10021228 10021230 10021233 10021234
10021251 10021253 10021260 10021261 10021263 10021264 10021266 10021270 10021275 10021277 10021283
10021595 10021596 10021598 10021601 10021621 10021623 10021624 10021627 10021628 10021631 10021638
10021642 10021649 10021657 10021661 10021672 10021675 10021678 10021684 10021710 10021715 10021721
10021723 10021727 10021733 10021738 10021765 10021768 10021771 10021774 10021779 10021787 10021793
10021797 10021805 10021810 10021813 10021820 10021822 10021847 10021848 10021855 10021856 10021864
10021865 10021884 10021885 10021890 10021895 10021899 10021908 10021909 10021953 10021959 10021960
10021961 10021963 10021969 10021971 10021980 10021982 10021984 10021985 10022003 10022012 10022013
10022014 10022016 10022017 10022024 10022029 10022030 10022033 10022034 10022036 10022040 10022041
10022045 10022056 10022059 10022061 10022066 10022068 10022069 10022074 10022082 10022085 10022088
10022120 10022246 10022249 10022292 10022335 10022341 10022382 10022417 10022424 10022456 10022464
10022466 10022548 10022713 10022721 10022735 10022745 10022747 10022749 10022752 10022755 10022762
10022769 10022774 10022816 10022819 10022827 10022831 10022843 10022844 10022846 10022848 10022850
10022851 10022868 10022870 10022872 10022891 10022898 10022899 10022911 10022943 10022946 10022954
10022955 10022956 10022985 10022989 10022990 10023002 10023007 10023009 10023010 10023015 10023027
10023029 10023035 10023046 10023049 10023051 10023057 10023062 10023063 10023097 10023098 10023101
10023191 10023249 10023251 10023405 10023412 10023445 10023448 10023451 10023478 10023480 10023508
10023527 10023601 10023603 10023611 10023619 10023620 10023623 10023626 10023631 10023636 10023651
10023652 10023662 10023674 10023680 10023696 10023701 10023703 10023705 10023706 10023708 10023710
10023711 10023738 10023740 10023744 10023746 10023750 10023751 10023752 10023757 10023758 10023760
10023761 10023762 10023763 10023770 10023775 10023785 10023786 10023788 10023797 10023804 10023805
10023806 10023819 10023820 10023825 10023828 10023837 10023842 10023848 10023850 10023855 10023879
10023934 10023936 10023938 10023940 10023941 10023942 10023946 10023957 10023963 10023967 10023972
10023973 10023979 10023982 10023984 10023990 10023991 10023993 10023997 10023999 10024047 10024054
10024061 10024066 10024071 10024096 10024097 10024099 10024102 10024109 10024111 10024117 10024125
10024149 10024158 10024164 10024165 10024169 10024174 10024179 10024195 10024196 10024212 10024222
10024224 10024282 10024296 10024299 10024302 10024321 10024323 10024326 10024327 10024331 10024333
10024342 10024355 10024359 10024364 10024371 10024373 10024375 10024391 10024393 10024395 10024397
10024398 10024433 10024498 10024500 10024505 10024556 10024562 10024749 10024750 10024795 10024831
10024837 10024850 10024855 10024861 10024863 10024871 10024872 10024899 10024904 10024906 10024908

10024912 10024913 10024915 10024958 10024963 10024974 10024977 10024991 10024996 10024998 10025007
10025009 10025012 10025013 10025014 10025020 10025021 10025022 10025028 10025029 10025034 10025040
10025041 10025043 10025045 10025065 10025066 10025067 10025073 10025076 10025078 10025081 10025088
10025090 10025124 10025125 10025134 10025141 10025145 10025148 10025157 10025161 10025162 10025167
10025171 10025175 10025201 10025252 10025341 10025346 10025368 10025379 10025380 10025388 10025389
10025606 10025858 10025944 10025987 10026194 10026237 10026406 10026410 10026414 10026996 10027066
10027086 10027149 10027246 10027307 10027487 10027666 10027786 10027808 10027988 10116054 10119794
20000203 20000243 20000420 20000460 20000563 20000580 20000581 20000600 20000601 20000620 20000685
20020607 20024872 20024997 20025005 20025017 20025029 20025037 20025038 20025045 20025048 20025049
20025249 20025509 20025518 20025564 20025661 20025666 20025667 20025735 20025741 20025747 20025752
20025758 20025770 20025780 20025784 20025787 20025798 20025799 20025817 20025818 20025828 20025831
20025836 20025850 20025930 20025932 20025940 20025946 20025955 20025956 20025958 20025964 20025971
20025973 20025981 20025989 20025990 20026004 20026013 20026026 20026027 20026028 20026041 20026157
20026159 20026161 20026171 20026176 20026180 20026183 20026190 20026194 20026198 20026202 20026205
20026209 20026213 20026259 20026264 20026265 20026268 20026269 20026271 20026277 20026284 20026285
20026288 20026289 20026296 20026297 20026298 20026305 20026306 20026309 20026310 20026311 20026312
20026315 20026318 20026320 20026323 20026325 20026328 20026330 20026331 20026343 20026344 20026355
20026357 20026363 20026366 20026375 20026408 20026409 20026412 20026418 20026419 20026421 20026426
20026430 20026439 20026440 20026441 20026443 20026447 20026449 20026452 20026458 20026468 20026469
20026472 20026475 20026478 20026484 20026491 20026496 20026498 20026499 20026500 20026501 20026504
20026515 20026516 20026518 20026519 20026520 20026522 20026534 20026573 20026579 20026581 20026585
20026588 20026590 20026593 20026600 20026627 20026632 20026634 20026638 20026639 20026645 20026657
20026661 20026667 20026679 20026708 20026717 20026809 20026822 20026839 20026840 20026842 20026850
20026857 20026975 20027185 20027191 20027236 20027320 20027323 20027352 20027354 20027366 20027367
20027368 20027373 20027374 20027380 20027381 20027425 20027506 20027510 20027514 20027550 20027578
20027583 20027615 20027666 20027674 20027697 20027738 20027739 20027740 20027751 20027755 20027757
20028175 20028179 20028180 20028181 20028182 20028183 20028184 20028215 20028216 20028227 20028228
20028231 20028234 20028235 20028237 20028238 20028239 20028240 20028249 20028250 20028262 20028552
20028559 20028560 20028583 20030035 20030408 20030409 20030783 20030879 20030884 20030895 20030908
20030920 20031156 20031160 20031161 20031163 20031172 20031195 20031196 20031200 20031202 20031207
20031209 20031216 20031218 20031220 20031221 20031229 20031230 20031233 20031243 20031246 20031247
20031248 20031261 20031281 20031533 20031537 20032281 20032288 20032290 20032300 20032313 20032315
20032318 20032320 20032337 20032338 20032341 20032342 20032345 20032350 20032366 20032369 20032372
20032374 20032388 20032397 20032402 20032405 20032409 20032413 20032656 20032658 20032674 20032680
20032683 20032693 20032694 20032707 20032717 20032732 20032735 20032736 20032737 20032738 20032739
20032744 20032763 20032764 20032772 20033033 20033037 20033065 20033067 20033075 20033076 20033114
20033121 20033122 20033124 20035460 20035474 20035478 20035513 20035516 20035520 20035542 20035553
20035564 20035567 20035568 20035569 20035838 20035845 20035848 20035859 20035869 20035872 20035875
20035887 20035888 20036212 20036214 20036233 20036236 20036579 20037328 20037330 20037341 20037348
20037355 20037375 20037382 20037392 20037395 20037723 20037726 20037728 20037735 20037736 20038075
20039213 20039221 20039226 20039234 20039236 20039244 20039259 20039263 20039271 20039274 20039276
20039278 20039283 20039285 20039286 20039287 20039304 20039319 20039324 20039328 20039329 20039572
20039575 20039577 20039643 20039654 20039660 20039661 20039676 20039700 20039945 20039953 20039954
20040320 20040328 20040348 20040351 20040359 20040367 20040384 20040386 20040395 20040408 20040409
20040410 20040441 20040442 20040695 20040736 20040737 20040742 20040769 20040783 20040797 20040801
20040811 20040814 20040820 20040821 20041086 20041110 20041817 20041820 20041821 20041825 20041829
20042563 20043312 20044060 20044463 20044473 20044481 20044499 20044512 20044519 20044846 20044851
20044853 20044854 20044860 20044867 20044878 20044908 20044910 20045183 20045185 20045186 20045187
20045189 20045197 20045201 20045202 20045221 20045223 20045239 20045240 20045241 20045248 20045259
20045275 20045278 20045282 20045285 20045565 20045567 20045572 20045577 20045590 20045591 20045592
20045597 20045602 20045606 20045610 20045612 20045614 20045630 20045642 20045643 20045646 20045647
20045649 20045650 20045652 20045657 20045659 20045660 20045666 20045673 20045676 20045678 20045682
20045929 20045940 20045951 20045958 20045964 20045968 20045980 20045992 20045995 20045996 20046677
20046678 20047058 20049298 20050043 20050050 20050052 20050060 20050062 20050066 20050077 20050079
20050082 20050085 20050087 20050088 20050092 20050096 20050098 20050102 20050107 20050114 20050123
20050141 20050142 20050143 20050145 20050148 20050150 20050153 20050159 20050168 20050169 20050187
20050189 20050190 20050418 20050419 20050420 20050421 20050422 20050424 20050428 20050435 20050440
20050441 20050445 20050447 20050453 20050454 20050460 20050478 20050495 20050496 20050519 20050545

20050548 20050554 20050555 20050556 20050558 20050793 20050794 20050796 20050797 20050798 20050800 20050802 20050805 20050808 20050810 20050811 20050812 20050813 20050814 20050819 20050821 20050842 20050856 20050857 20050858 20050861 20050862 20050863 20050864 20050885 20050898 20050929 20050932 20050937 20050939 20051165 20051169 20051175 20051176 20051179 20051180 20051181 20051727 20051733 20051741 20051743 20051744 20051746 20051753 20051767 20051768 20051769 20051771 20051801 20051806 20051810 20051815 20051816 20051817 20051820 20051841 20051849 20051858 20051861 20051863 20051864 20051865 20052106 20052129 20052130 20052133 20052135 20052145 20052147 20052148 20052155 20052156 20052160 20052163 20052166 20052169 20052170 20052173 20052178 20052181 20052182 20052184 20052192 20052205 20052478 20052479 20052494 20052495 20052496 20052500 20052522 20052524 20052529 20052531 20052533 20052535 20052537 20052538 20052540 20052541 20052542 20052549 20052552 20052554 20052556 20052562 20052566 20052575 20052579 20052581 20052585 20052586 20052849 20052852 20052853 20052854 20052856 20052859 20052863 20052872 20052873 20052877 20052882 20052887 20052892 20052893 20052895 20052897 20052898 20052899 20052904 20052906 20052909 20052917 20052918 20052919 20052920 20052923 20052930 20052931 20052938 20052947 20052950 20052955 20052957 20052970 20052972 20052976 20052981 20053225 20053227 20053230 20053234 20053241 20053243 20053247 20053270 20053274 20053275 20053291 20053317 20053334 20053971 20053972 20054345 20054350 20054352 20054357 20054366 20054371 20054373 20054374 20054377 20054381 20054382 20054453 20054456 20054457 20054460 20054461 20054463 20054466 20054467 20054469 20054470 20054474 20054482 20054722 20054727 20054729 20054795 20054797 20054798 20054806 20054830 20054841 20054842 20054843 20054844 20054848 20054851 20055119 20055120 20055126 20055129 20055133 20055140 20055144 20055155 20055161 20055166 20055171 20055184 20055185 20055187 20055190 20055192 20055193 20055485 20055496 20055498 20055504 20055511 20055512 20055515 20055518 20055520 20055522 20055523 20055529 20055530 20055554 20055565 20055570 20055573 20055841 20055842 20055868 20055889 20055893 20062594 20068932 20071548 20071923 20071924 20078093 20078467 20078475 20084079 20091745 20092122 20092495 20093245 20096439 20096442 20096488 20096490 20096493 20096520 20096523 20096531 20096793 20096806 20096822 20096832 20096836 20096849 20096850 20096851 20096852 20096866 20096871 20096890 20096893 20096903 20096923 20097169 20097203 20097214 20097217 20097219 20097222 20097224 20097225 20097240 20097242 20097249 20097559 20097587 20097594 20097603 20097616 20097621 20097637 20097640 20097642 20097645 20097652 20097661 20099226 20099228 20099603 20099973 20099976 20100348 20101842 20102216 20102591 20102965 20103712 20103713 20103714 20103719 20103720 20103723 20103729 20105583 20105966 20108960 20108969 20108979 20109329 20109330 20109333 20109339 20109340 20109347 20109349 20109350 20109379 20109395 20109399 20109400 20109404 20109405 20109407 20109416 20109417 20109418 20109423 20109442 20109699 20109705 20109706 20109712 20109719 20110071 20110073 20110074 20110077 20110088 20110094 20110096 20111567 20111940 20111941 20111959 20111967 20111983 20112344 20112351 20112352 20112359 20112693 20112695 20112700 20112702 20112714 20112715 20119795 20121665 20121667 20121676 20121678 20122050 20122051 20122061 20122062 20122075 20122082 20122085 20122087 20122089 20122102 20122104 20122106 20122420 20122443 20122444 20122447 20122449 20122450 20122454 20122455 20122456 20122462 20122463 20122464 20122465 20122467 20122470 20122472 20122474 20122480 20122484 20122492 20122796 20122802 20122803 20122832 20122833 20122838 20122839 20122842 20122845 20122849 20122853 20122858 20122859 20122860 20122864 20122865 20123171 20123182 20123183 20123185 20123187 20123189 20123192 20123196 20123198 20123201 20123208 20123211 20123223 20123225 20123230 20123236 20123237 20123238 20123241 20123244 20123247 20123535 20123542 20123558 20123567 20123569 20123570 20123572 20123596 20123597 20123603 20123613 20123616 20123908 20123909 20123911 20123912 20123929 20123946 20123955 20123958 20123959 20123970 20123973 20123979 20123984 20128770 20132324 20132889 20132900 20132902 20132913 20132914 20132918 20132920 20132921 20132926 20132930 20132931 20132935 20132936 20132951 20132952 20132961 20132963 20132966 20132967 20133259 20133265 20133267 20133276 20133278 20133279 20133285 20133301 20133304 20133305 20133306 20133308 20133309 20133313 20133317 20133320 20133324 20133637 20133645 20133654 20133655 20133662 20133664 20135129 20137933 20138494 20140738 20141862 20141863 20141864 20141871 20141873 20141883 20141884 20141907 20141910 20141911 20141915 20141917 20141921 20141922 20141924 20141925 20141930 20141932 20141950 20141952 20142238 20142239 20142240 20142241 20142245 20142248 20142266 20142284 20142287 20142290 20142294 20142303 20142308 20142312 20142609 20142616 20142621 20142627 20142629 20142632 20142634 20142636 20142638 20142643 20142645 20142649 20142650 20142656 20142658 20142683 20142685 20142686 20143183 20143184 20143204 20143205 20143206 20143209 20143213 20143214 20143218 20143219 20143221 20143223 20143224 20143227 20143232 20143234 20143240 20143242 20143243 20143244 20143251 20143254 20143545 20143551 20143554 20143559 20143560 20143561 20143562 20143564 20143565 20143569 20143570 20143572 20143575 20143579 20143583 20143586 20143593 20143601 20143604 20143606 20143610 20143617 20143620 20143626 20143917 20143919 20143921 20143934 20143937 20143959 20143969 20143974 20143975 20143976 20143981 20143982 20144294 20144338 20144341 20144349 20144352 20144353 20144354

20144356 20144359 20144363 20144368 20144687 20144689 20144694 20145048 20145066 20145109 20145115
20145116 20145417 20145418 20145419 20145420 20145421 20145422 20145447 20145448 20145450 20145454
20145456 20145458 20145466 20145474 20145488 20145497 20145794 20145798 20145801 20145817 20145827
20145829 20145834 20145836 20145842 20145843 20145853 20145856 20145859 20146174 20146176 20146182
20146183 20146185 20146186 20146187 20146188 20146195 20146196 20146208 20146225 20146235 20146240
20146535 20146551 20146558 20146567 20146571 20146574 20146583 20146584 20146588 20146589 20146600
20146611 20146910 20146914 20146915 20146918 20146932 20146934 20146943 20146944 20146946 20146951
20146958 20147284 20147285 20147286 20147293 20147295 20147297 20147299 20147307 20147309 20147312
20147315 20168002 20168332 20171388 20172771 20173306 20173727 20173977 20176233 20177277 20179636
20179638 20179822 20179827 20179831 20179832 20179840 20179848 20179849 20179859 20179864 20179869
20179870 20179873 20179879 20179880 20179882 20179883 20179884 20179887 20179893 20179900 20179902
20179905 20179914 20179915 20179919 20179922 20179925 20179937 20179942 20179952 20179955 20179958
20179971 20179975 20179978 20179984 20179988 20179989 20179992 20179995 20179999 20180005 20180013
20180026 20180029 20180030 20180055 20180056 20180064 20180065 20180066 20180090 20180098 20180100
20180107 20180110 20180115 20180116 20180124 20180130 20180133 20180135 20180138 20180143 20180148
20180149 20180152 20180164 20180166 20180169 20180179 20180186 20180198 20180205 20180210 20180216
20180217 20180224 20180235 20180237 20180238 20180239 20180272 20180276 20180285 20180287 20180291
20180292 20180293 20180297 20180301 20180303 20180311 20180313 20180316 20180320 20180323 20180324
20180331 20180341 20180344 20180349 20180353 20180354 20180356 20180373 20180377 20180381 20180386
20180395 20180406 20180408 20180412 20180415 20180416 20180417 20180426 20180428 20180437 20180440
20180447 20180449 20180456 20180458 20180459 20180461 20180462 20180464 20180474 20180475 20180481
20180482 20180490 20180492 20180495 20180497 20180503 20180510 20180511 20180516 20180521 20180526
20180528 20180530 20180536 20180540 20180543 20180544 20180561 20180574 20180589 20180605 20180607
20180613 20180629 20180630 20180640 20180648 20180651 20180658 20180659 20180663 20180673 20180685
20180702 20180704 20180705 20180708 20180709 20180711 20180713 20180715 20180716 20180723 20180725
20180731 20180732 20180734 20180738 20180739 20180751 20180755 20180759 20180766 20180769 20180775
20180776 20180782 20180785 20180788 20180790 20180792 20180798 20180803 20180813 20180814 20180819
20180822 20180823 20180824 20180828 20180829 20180835 20180838 20180848 20180856 20180873 20180879
20180880 20180882 20180884 20180885 20180888 20180890 20180910 20180911 20180912 20180913 20180919
20180923 20180924 20180925 20180926 20180927 20180929 20180930 20180938 20180941 20180943 20180951
20180956 20180969 20180971 20180975 20180984 20180985 20180990 20180997 20181002 20181008 20181011
20181014 20181016 20181024 20181034 20181036 20181042 20181046 20181047 20181057 20181059 20181065
20181079 20181097 20181104 20181105 20181109 20181112 20181118 20181119 20181120 20181123 20181124
20181126 20181134 20181136 20181137 20181141 20181147 20181150 20181160 20181164 20181170 20181171
20181179 20181183 20181188 20181191 20181192 20181198 20181208 20181219 20181220 20181226 20181229
20181230 20181231 20181232 20181234 20181235 20181238 20181243 20181245 20181246 20181250 20181253
20181260 20181262 20181264 20181268 20181271 20181276 20181283 20181286 20181290 20181295 20181301
20181303 20181346 20181353 20181354 20181371 20181377 20181383 20181390 20181392 20181402 20181409
20181410 20181411 20181414 20181421 20181425 20181434 20181448 20181473 20181485 20181487 20181498
20181500 20181504 20181507 20181508 20181509 20181519 20181524 20181526 20181536 20181542 20181544
20181548 20181553 20181554 20181556 20181557 20181559 20181560 20181570 20181577 20181580 20181582
20181599 20181613 20181614 20181618 20181622 20181634 20181638 20181649 20181654 20181656 20181675
20181688 20181704 20181706 20181709 20181714 20181716 20181723 20181725 20181730 20181735 20181741
20181742 20181743 20181744 20181745 20181756 20181757 20181764 20181769 20181788 20181790 20181804
20181806 20181808 20181809 20181818 20181819 20181825 20181830 20181839 20181841 20181849 20181853
20181869 20181873 20181876 20181886 20181889 20181891 20181892 20181902 20181905 20181909 20181915
20181916 20181921 20181931 20181935 20181942 20181945 20181951 20181964 20181967 20181977 20181981
20181988 20181992 20181999 20182000 20182002 20182004 20182008 20182018 20182020 20182026 20182041
20182043 20182045 20182048 20182051 20182055 20182057 20182062 20182074 20182079 20182088 20182097
20182098 20182103 20182106 20182111 20182120 20182123 20182124 20182127 20182130 20182131 20182136
20182138 20182142 20182159 20182160 20182167 20182174 20182179 20182181 20182182 20182188 20182189
20182192 20182196 20182197 20182201 20182203 20182211 20182214 20182221 20182224 20182226 20182228
20182229 20182231 20182232 20182234 20182241 20182251 20182254 20182265 20182268 20182270 20182271
20182272 20182273 20182276 20182278 20182281 20182285 20182286 20182292 20182294 20182309 20182311
20182315 20182316 20182328 20182331 20182334 20182335 20182342 20182355 20182369 20182370 20182372
20182373 20182378 20182381 20182383 20182385 20182393 20182395 20182399 20182401 20182406 20182422
20182424 20182432 20182433 20182443 20182444 20182446 20182465 20182469 20182472 20182486 20182496
20182498 20182502 20182504 20182521 20182532 20182536 20182545 20182554 20182555 20182565 20182568

20182572 20182586 20182591 20182594 20182596 20182600 20182605 20182608 20182611 20182616 20182617 20182620 20182623 20182629 20182636 20182646 20182649 20182651 20182658 20182659 20182671 20182672 20182675 20182680 20182685 20182688 20182691 20182695 20182701 20182703 20182704 20182705 20182715 20182718 20182722 20182726 20182733 20182734 20182739 20182752 20182754 20182758 20182762 20182766 20189359 20190481 20191607 20191610 20191616 20191617 20191619 20191621 20191639 20191642 20191644 20191645 20191648 20191649 20191654 20191666 20191667 20191671 20191673 20191676 20191679 20191680 20191683 20191687 20191689 20191694 20191696 20191710 20191714 20191715 20191718 20191721 20191726 20191731 20191733 20191760 20191774 20191778 20191782 20191785 20191794 20191802 20191804 20191805 20191810 20191812 20191813 20191824 20191830 20191832 20191833 20191845 20191850 20191852 20191856 20191858 20191872 20191874 20191878 20191897 20191904 20191918 20191921 20191925 20191926 20191927 20191931 20191934 20191935 20191936 20191938 20191950 20191959 20191962 20191967 20191968 20191970 20191973 20191974 20191976 20191979 20191983 20191984 20191994 20192000 20192002 20192019 20192021 20192027 20192726 20193847 20198900 20198908 20198915 20198921 20198925 20198927 20198928 20198941 20198950 20198966 20198976 20198995 20199000 20199001 20199010 20199025 20199034 20199048 20199061 20200018 20200022 20200027 20200032 20200036 20200038 20200054 20200057 20200059 20200060 20200067 20200069 20200070 20200081 20200082 20200083 20200089 20200097 20200098 20200099 20200111 20200122 20200127 20200146 20200149 20200155 20200187 20200192 20200193 20200197 20200198 20200219 20200258 20200260 20200264 20200282 20200286 20200305 20200589 20200591 20200593 20200596 20200611 20200616 20200617 20200621 20200627 20200632 20200633 20200640 20200645 20200657 20200658 20200659 20200676 20200715 20200716 20200718 20200720 20200721 20200722 20200724 20200727 20200739 20200753 20200764 20200771 20200780 20200782 20200795 20200798 20200803 20200810 20200811 20200815 20200816 20200818 20200826 20200838 20200846 20200851 20200853 20200855 20200864 20200877 20200878 20200883 20200886 20200901 20200904 20200926 20200935 20200943 20200947 20200956 20200958 20200959 20201327 20201342 20201344 20201347 20201355 20201360 20201374 20201375 20201377 20201383 20201384 20201386 20201389 20201396 20201397 20201398 20201401 20201406 20201414 20201419 20201421 20201428 20201429 20201435 20201442 20201445 20201465 20201701 20202465 20202483 20203198 20203760 20203770 20203777 20203786 20203791 20203792 20203795 20203796 20203797 20203817 20203833 20203836 20203838 20203839 20203842 20203843 20204132 20204136 20204137 20204141 20204145 20204162 20204174 20204178 20204196 20204203 20204207 20204216 20204218 20204220 20204230 20204231 20204232 20204235 20204242 20204250 20204260 20204265 20204281 20204283 20204284 20204288 20204294 20204295 20204302 20204307 20204316 20204321 20204325 20204326 20204327 20204329 20204342 20204344 20204357 20204366 20204696 20204697 20204704 20204710 20204716 20204720 20204727 20204728 20204731 20204732 20204737 20204739 20204748 20205068 20205815 20205824 20205825 20206190 20206203 20206205 20206207 20206212 20206216 20206221 20206228 20206231 20206234 20206238 20206248 20206259 20206279 20207314 20207318 20207319 20207320 20207324 20207328 20207350 20207354 20207357 20207371 20207381 20207385 20207387 20207390 20207407 20207416 20207419 20207423 20207424 20207437 20207441 20207447 20207450 20207455 20207462 20207463 20207468 20207474 20207484 20207486 20207509 20207515 20207523 20207526 20207532 20207554 20207556 20207559 20207562 20207563 20207578 20207594 20207883 20207885 20207896 20207906 20207907 20207908 20207909 20207912 20207914 20207915 20207934 20207947 20207948 20207957 20207958 20207969 20207976 20207979 20207995 20208017 20208021 20208031 20208037 20208050 20208051 20208059 20208072 20208088 20208091 20208101 20208105 20208107 20208112 20208113 20208118 20208119 20208121 20208127 20208131 20208137 20208139 20208148 20208156 20208163 20208188 20208189 20208190 20208193 20208194 20208196 20208201 20208202 20208218 20208222 20208224 20208225 20208228 20208234 20208235 20208248 20208249 20208253 20208254 20208267 20208279 20208291 20208297 20208303 20208308 20208316 20208317 20208318 20208321 20208328 20208334 20208339 20208346 20208351 20208356 20208359 20208363 20208373 20208377 20208379 20208381 20208385 20208389 20208393 20208394 20208400 20208414 20208417 20208420 20208428 20208437 20208438 20208439 20208442 20208450 20208455 20208463 20208466 20208470 20208472 20208478 20208479 20208488 20208496 20208508 20208513 20208532 20208535 20208545 20208548 20208550 20208557 20208558 20208560 20208561 20208565 20208569 20208570 20208572 20208575 20208580 20208586 20208588 20208589 20208604 20208609 20208610 20208611 20208629 20208631 20208635 20208642 20208648 20208654 20208661 20208665 20208669 20208674 20208678 20208685 20208689 20208707 20208711 20208722 20208725 20208729 20208738 20208745 20208747 20208754 20208758 20208760 20208761 20208767 20208776 20208783 20208785 20208789 20208792 20208793 20208795 20208807 20208808 20208810 20208812 20208820 20208824 20208828 20208835 20208847 20208855 20208861 20208864 20208874 20208884 20208888 20208897 20208907 20208919 20208932 20208936 20208955 20208972 20208974 20208984 20208995 20209012 20209017 20209019 20209022 20209023 20209025 20209042 20209043 20209045 20209047 20209060 20209061 20209069 20209076 20209081 20209082 20209090 20209091 20209099 20209104 20209105 20209110 20209115 20209124 20209129 20209131 20209142 20209144 20209151 20209157 20209160 20209167 20209169 20209176 20209177 20209186 20209199

20209205 20209209 20209225 20209228 20209239 20209243 20209248 20209254 20209259 20209260 20209267
20209275 20209279 20209286 20209296 20209300 20209307 20209314 20209321 20209326 20209327 20209333
20209337 20209344 20209352 20209357 20209358 20209359 20209371 20209375 20209391 20209395 20209404
20209405 20209411 20209413 20209417 20209423 20209424 20209427 20209436 20209439 20209444 20209446
20209473 20209489 20209497 20209498 20209506 20209507 20209522 20209523 20209532 20209537 20209539
20209541 20209557 20209564 20209566 20209567 20209592 20209599 20209609 20209614 20209615 20209618
20209624 20209627 20209629 20209632 20209645 20209651 20209652 20209670 20209687 20209696 20209697
20209698 20209704 20209710 20209713 20209719 20209730 20209732 20209735 20209748 20209749 20209750
20209753 20209756 20209757 20209758 20209765 20209771 20209793 20209806 20209814 20209823 20209830
20209833 20209835 20209836 20209839 20209842 20209845 20209847 20209854 20209856 20209857 20209873
20209883 20209888 20209898 20212362 20212365 20212368 20212369 20212372 20212377 20212400 20212402
20212404 20212411 20212414 20212420 20212422 20212439 20212440 20212441 20212443 20212445 20212448
20212452 20212456 20212463 20212467 20212468 20212477 20212481 20212482 20212483 20212484 20212485
20212488 20212500 20212502 20212504 20212517 20212527 20212529 20212532 20212533 20212537 20212538
20212544 20212557 20212561 20212567 20212576 20212582 20212585 20212588 20212589 20212590 20212591
20212593 20212597 20212598 20212606 20212607 20212614 20212616 20212617 20212618 20212628 20212634
20212637 20212641 20212643 20212646 20212655 20212659 20212665 20212673 20212674 20212675 20212676
20212682 20212686 20212695 20212701 20212705 20212722 20212723 20212728 20212735 20212738 20212748
20212752 20212762 20212767 20212771 20212780 20212782 20212783 20212786 20212796 20212802 20212805
20212806 20212807 20212810 20212824 20212827 20212831 20212833 20212845 20212846 20212869 20212877
20212885 20212891 20212892 20212893 20212894 20212895 20212908 20212928 20212929 20212938 20212946
20212947 20212951 20212963 20212964 20212965 20212968 20212970 20212978 20212987 20212990 20212994
20213000 20213005 20213009 20213022 20213030 20213031 20213034 20213041 20213047 20213049 20213055
20213066 20213072 20213075 20213080 20213086 20213087 20213088 20213090 20213092 20213095 20213096
20213097 20213098 20213104 20213113 20213115 20213141 20213145 20213146 20213164 20213167 20213171
20213174 20213178 20213180 20213181 20213182 20213186 20213190 20213192 20213199 20213203 20213206
20213210 20213226 20213239 20213245 20213249 20213254 20213262 20213263 20213279 20213287 20213290
20213296 20213300 20213302 20213307 20213311 20213316 20213331 20213333 20213335 20213340 20213344
20213353 20213357 20213362 20213374 20213376 20213377 20213382 20213391 20213396 20213399 20213400
20213402 20213405 20213414 20213418 20213429 20213432 20213441 20213442 20213444 20213446 20213450
20213453 20213458 20213461 20213462 20213463 20213466 20215914 20216662 20217971 20217972 20217983
20217984 20217985 20217987 20217990 20217991 20217993 20217998 20217999 20218000 20218006 20218011
20218014 20218020 20218024 20218025 20218028 20218047 20218051 20218054 20218055 20218058 20218062
20218068 20218071 20218079 20218080 20218083 20218085 20218086 20218088 20218095 20218097 20218101
20218102 20218105 20218114 20218118 20218119 20218122 20218123 20218124 20218125 20218130 20218133
20218147 20218152 20218159 20218160 20218161 20218165 20218166 20218168 20218177 20218181 20218182
20218186 20218187 20218189 20218192 20218197 20218199 20218200 20218204 20218207 20218216 20218218
20218220 20218222 20218230 20218233 20218234 20218247 20218248 20218252 20218253 20218270 20218271
20218723 20218727 20218733 20218735 20218739 20218747 20218750 20218754 20218755 20218762 20218765
20218767 20218775 20218778 20218786 20218791 20218794 20218795 20218797 20218799 20218800 20218805
20218806 20218808 20218809 20218812 20218817 20219467 20220215 20220596 20220598 20220599 20220601
20220964 20220967 20221340 20221342 20221357 20221358 20221359 20221361 20221363 20221372 20221373
20221380 20221384 20221390 20221402 20221409 20221420 20221429 20221431 20221435 20221436 20221440
20221441 20221447 20221461 20221477 20221478 20221480 20221481 20221483 20221492 20221496 20221497
20221500 20221503 20221506 20221508 20221510 20221511 20221516 20221522 20221526 20221528 20221529
20221532 20221534 20221537 20221542 20221548 20221551 20221553 20221566 20221571 20221573 20221585
20221590 20221596 20221597 20221609 20221610 20221614 20221618 20221621 20221622 20221625 20221632
20221645 20221653 20221658 20221661 20221662 20221664 20221665 20221677 20221695 20221699 20221704
20221709 20221714 20221723 20221728 20221742 20222085 20222461 20223583 20223955 20223956 20223957
20224154 20224155 20224156 20224158 20224170 20224172 20224175 20224179 20224180 20224182 20224186
20224187 20224193 20224197 20224199 20224201 20224202 20224215 20224226 20224250 20224252 20224254
20224257 20224258 20224259 20224262 20224263 20224280 20224282 20224283 20224292 20224300 20224309
20224319 20224321 20224330 20224332 20224333 20224342 20224348 20224349 20224354 20224355 20224363
20224369 20224373 20224383 20224388 20224401 20224413 20226947 20226955 20226968 20226972 20226974
20226982 20226984 20226985 20226988 20226992 20226997 20226999 20227002 20227008 20227011 20227013
20227014 20227021 20227022 20227024 20227028 20227030 20227035 20227037 20227041 20227044 20227054
20227057 20227058 20227059 20227065 20227066 20227067 20227069 20227070 20227071 20227076 20227077
20227331 20227343 20227352 20227358 20227362 20227365 20227369 20227370 20227371 20227376 20227383

20227393 20227397 20227400 20227407 20227408 20227409 20227411 20227412 20227413 20227418 20227428 20227431 20227438 20227448 20227450 20227455 20227457 20227459 20227461 20227464 20229941 20229948 20229959 20229960 20229963 20229966 20229969 20229974 20229998 20230004 20230007 20230009 20230011 20230012 20230025 20230028 20230030 20230034 20230046 20230047 20230052 20230059 20230062 20230068 20230070 20230071 20230073 20230080 20230087 20230089 20230093 20230094 20230098 20230105 20230107 20230109 20230115 20230124 20230136 20230138 20230139 20230140 20230141 20230150 20230152 20230162 20230166 20230172 20230177 20230185 20230187 20230190 20230198 20230203 20230204 20230205 20230207 20230212 20230216 20230236 20230240 20230244 20230259 20230263 20230269 20230277 20230280 20230287 20230288 20230289 20230294 20230298 20230320 20230332 20230336 20230337 20230341 20230346 20230350 20230351 20230356 20230357 20230365 20230368 20230372 20230375 20230376 20230384 20230389 20230393 20230399 20230402 20230404 20230406 20230407 20230415 20230416 20230419 20230422 20230427 20230432 20230439 20230441 20230443 20230448 20230456 20230465 20230482 20230483 20230487 20230488 20230490 20230492 20230499 20230502 20230510 20230523 20230526 20230532 20230538 20230540 20230542 20230549 20230552 20230554 20230559 20230565 20230568 20230570 20230571 20230578 20230585 20230595 20230600 20230612 20230617 20230622 20230625 20230632 20230634 20230636 20230639 20230644 20230648 20230657 20230658 20230672 20230673 20230675 20230676 20233868 20233871 20233874 20233877 20233881 20233884 20233891 20233896 20233898 20233902 20233907 20233912 20233914 20233917 20233918 20233926 20233931 20233945 20233954 20233955 20233956 20233965 20233966 20233971 20233980 20233981 20233986 20233987 20233991 20233995 20234001 20234022 20234026 20234029 20234030 20234032 20234035 20234046 20234047 20234048 20234055 20234062 20234067 20234069 20234070 20234073 20234074 20234086 20234091 20234098 20234103 20234110 20234120 20237236 20237241 20237250 20237254 20237257 20237259 20237263 20237268 20237272 20237277 20237281 20237290 20237292 20237300 20237318 20237321 20237326 20237328 20237332 20237334 20237349 20237371 20237382 20237386 20237392 20242099 20242103 20242114 20242129 20242135 20242136 20242139 20242141 20242149 20242165 20242173 20242179 20242182 20242185 20242187 20242196 20242202 20242209 20242210 20242212 20242214 20242220 20242230 20242234 20242236 20242237 20242238 20242239 20242242 20242248 20242258 20242265 20242266 20242273 20242274 20242278 20242279 20242284 20242287 20242294 20242298 20242301 20242303 20242308 20242313 20242317 20242323 20242329 20242334 20242336 20242338 20242346 20242347 20242355 20242364 20242371 20242392 20242395 20242398 20242403 20242409 20242411 20242414 20242416 20242422 20246789 20246793 20246807 20246819 20246822 20246825 20246827 20246833 20246843 20246844 20246858 20246861 20246862 20246871 20246872 20246876 20246879 20246881 20246886 20246894 20246907 20246908 20246909 20246911 20246923 20246927 20246939 20246942 20246948 20246967 20246969 20246970 20246973 20246980 20249049 20249066 20249087 20249088 20249107 20249111 20249124 20249131 20249134 20249137 20249142 20249168 20249170 20249184 20249226 20249257 20249259 20249263 20249292 30000100 30000220 30000241 30000260 30000280 30000300 30000340 30000460 30000480 30000500 30000600 30000601 30000604 30000632 30000730 30000765 30000766 30000770 30000790 30001000 30001080 30001540 30001572 30078467 30079589 30080340 30080346 30081089 30095492 30095496 30095874 30095876 30095879 30096241 30096617 30096629 30096634 30096635 30096993 30097373 30098293 30100161 30100164 30100167 30100176 30100178 30100186 30100199 30100207 30100209 30100210 30100218 30100222 30100224 30100225 30100232 30100243 30100246 30100254 30100259 30100260 30100270 30100286 30100540 30100542 30100543 30100550 30100553 30100572 30100576 30100577 30100579 30100585 30100590 30100604 30100609 30100618 30100621 30100623 30100637 30100645 30100648 30100649 30100651 30100653 30100657 30100659 30100667 30100671 30100678 30100909 30100912 30100915 30100916 30100917 30100928 30100929 30100933 30100947 30100948 30100970 30100983 30100984 30101020 30101021 30101285 30101287 30101301 30101310 30101311 30101313 30101314 30101315 30101316 30101318 30101322 30101330 30101337 30101338 30101339 30101340 30101341 30101342 30101353 30101357 30101358 30101359 30101366 30101372 30101375 30101664 30101687 30101690 30101698 30101701 30101707 30101709 30101710 30101717 30101719 30101724 30101742 30101756 30101769 30102049 30102050 30102054 30102056 30102075 30102087 30102088 30102089 30102090 30102094 30102114 30102115 30102156 30103156 40022454 40055374 40057048 40072630 40093880 40097666 40097671 40097674 40097716 40099299 40104204 40109486 40114828 40115576 40116004 40116015 40116016

7 Reassignment Examples Shift Data

Similar tables of data are available for the stereochemistry examples in DP4-AI supporting information [1]

7.1 S1

label	calc	corrected	exp	error	prob
C12	24.52	17.26	18.70	1.44	0.02
C1	27.97	20.23	20.70	0.47	0.71
C15	29.12	21.23	24.40	3.17	0.27
C8	29.27	21.36	24.60	3.24	0.62
C13	30.35	22.29	26.90	4.61	0.07
C10	32.58	24.22	28.10	3.88	0.18
C7	37.80	28.72	28.20	-0.52	0.65
C3	40.14	30.75	28.60	-2.15	0.14
C2	41.44	31.86	32.80	0.94	0.39
C11	50.84	39.98	37.60	-2.38	0.49
C9	52.25	41.20	39.10	-2.10	0.69
C4	55.31	43.84	40.10	-3.74	0.18
C5	71.91	58.18	43.70	-14.48	0.01
C6	82.80	67.58	75.20	7.62	0.01

Incorrect isomer

label	calc	corrected	exp	error	prob
C11	21.14	18.33	18.70	0.37	0.41
C13	24.86	21.84	20.70	-1.14	0.17
C14	27.27	24.11	24.40	0.29	0.68
C8	27.31	24.15	24.60	0.45	0.57
C10	27.94	24.74	26.90	2.16	0.59
C7	31.26	27.87	28.10	0.23	0.68
C5	31.55	28.14	28.20	0.06	0.61
C1	33.00	29.51	28.60	-0.91	0.17
C4	35.53	31.89	32.80	0.91	0.65
C6	43.05	38.98	37.60	-1.38	0.09
C9	43.44	39.35	39.10	-0.25	0.65
C12	46.14	41.90	40.10	-1.80	0.32
C2	47.59	43.26	43.70	0.44	0.71
C3	80.87	74.62	75.20	0.58	0.65

Correct Isomer

7.2 S2

label	calc	corrected	exp	error	prob
C13	12.37	3.49	7.70	4.21	0.08
C14	20.13	12.03	19.72	7.69	0.01
C17	55.99	51.50	55.81	4.31	0.07
C15	61.86	57.96	56.46	-1.50	0.06
C1	104.55	104.96	91.80	-13.16	0.01
C2	120.50	122.51	108.70	-13.81	0.01
C8	121.19	123.27	111.34	-11.93	0.01
C4	123.61	125.94	157.05	31.11	0.01
C5	139.96	143.94	157.05	13.11	0.01
C3	154.21	159.63	159.07	-0.56	0.70
C6	161.04	167.14	161.14	-6.00	0.01
C9	168.16	174.98	162.05	-12.93	0.01
C7	171.43	178.57	178.03	-0.54	0.71

Incorrect isomer, original NMR interpretation

label	calc	corrected	exp	error	prob
C13	21.86	21.48	19.72	-1.76	0.15
C16	56.12	56.06	55.81	-0.25	0.70
C15	56.20	56.14	56.46	0.32	0.68
C4	95.51	95.83	91.80	-4.03	0.36
C6	98.33	98.68	108.70	10.02	0.01
C2	113.77	114.27	111.34	-2.93	0.37
C8	117.30	117.83	157.05	39.22	0.01
C3	166.09	167.09	157.05	-10.04	0.01
C1	168.01	169.02	159.07	-9.95	0.01
C5	169.84	170.87	161.14	-9.73	0.01
C9	169.86	170.89	162.05	-8.84	0.01
C7	178.94	180.06	178.03	-2.03	0.49

Correct Isomer, original NMR interpretation

label	calc	corrected	exp	error	prob
C13	12.37	3.26	7.30	4.04	0.08
C14	20.13	11.47	19.40	7.93	0.01
C17	55.99	49.41	55.40	5.99	0.01
C15	61.86	55.62	56.00	0.38	0.71
C1	104.55	100.78	91.10	-9.68	0.01
C2	120.50	117.66	105.30	-12.36	0.01
C8	121.19	118.39	108.00	-10.39	0.01
C4	123.61	120.96	110.90	-10.06	0.01
C5	139.96	138.25	156.50	18.25	0.01
C3	154.21	153.33	158.50	5.17	0.06
C6	161.04	160.55	160.70	0.15	0.55
C9	168.16	168.08	162.60	-5.48	0.11
C7	171.43	171.54	177.60	6.06	0.01

Incorrect isomer, updated NMR interpretation

label	calc	corrected	exp	error	prob
C13	21.86	21.97	19.40	-2.57	0.53
C16	56.12	54.70	55.40	0.70	0.71
C15	56.20	54.78	56.00	1.22	0.06
C4	95.51	92.34	91.10	-1.24	0.30
C6	98.33	95.04	105.30	10.26	0.01
C2	113.77	109.79	108.00	-1.79	0.34
C8	117.30	113.17	110.90	-2.27	0.69
C3	166.09	159.78	156.50	-3.28	0.42
C1	168.01	161.62	158.50	-3.12	0.30
C5	169.84	163.37	160.70	-2.67	0.69
C9	169.86	163.39	162.60	-0.79	0.32
C7	178.94	172.06	177.60	5.54	0.02

Correct Isomer, updated NMR interpretation

7.3 S3

label	calc	corrected	exp	error	prob
C22	15.78	12.52	14.00	1.48	0.18
C11	22.14	18.66	14.50	-4.16	0.17
C21	26.76	23.11	22.60	-0.51	0.70
C5	27.80	24.11	24.30	0.19	0.70
C16	31.39	27.58	28.40	0.82	0.71
C17	33.01	29.14	29.20	0.06	0.71
C19	33.05	29.18	29.50	0.32	0.72
C18	33.46	29.58	29.60	0.02	0.72
C20	35.24	31.30	30.30	-1.00	0.72
C6	35.60	31.65	30.50	-1.15	0.51
C15	35.88	31.92	31.80	-0.12	0.72
C4	36.56	32.56	32.20	-0.36	0.59
C13	61.02	56.16	59.40	3.24	0.57
C2	112.43	105.77	132.20	26.43	0.01
C10	150.33	142.33	138.90	-3.43	0.18
C3	160.45	152.10	139.00	-13.10	0.01
C7	169.96	161.28	147.50	-13.78	0.01
C9	170.26	161.57	172.80	11.23	0.01
C1	210.92	200.80	194.60	-6.20	0.01

Incorrect isomer

label	calc	corrected	exp	error	prob
C24	14.51	13.01	14.00	0.99	0.68
C20	16.01	14.46	14.50	0.04	0.61
C19	26.62	24.75	22.60	-2.15	0.49
C5	27.61	25.71	24.30	-1.41	0.63
C14	29.86	27.88	28.40	0.52	0.73
C15	30.87	28.87	29.20	0.33	0.72
C16	31.05	29.05	29.50	0.45	0.73
C13	31.82	29.79	29.60	-0.19	0.55
C17	32.16	30.12	30.30	0.18	0.67
C4	33.09	31.03	30.50	-0.53	0.73
C18	34.72	32.60	31.80	-0.80	0.70
C6	35.40	33.26	32.20	-1.06	0.66
C23	57.97	55.14	59.40	4.26	0.07
C2	136.65	131.43	132.20	0.77	0.49
C22	143.49	138.06	138.90	0.84	0.44
C3	145.42	139.94	139.00	-0.94	0.41
C8	154.02	148.27	147.50	-0.77	0.47
C9	178.50	172.00	172.80	0.80	0.16
C1	203.17	195.92	194.60	-1.32	0.23

Correct Isomer

7.4 S4

label	calc	corrected	exp	error	prob
C12	23.37	24.82	18.70	-6.12	0.03
C11	28.77	30.08	20.80	-9.28	0.01
C10	29.54	30.83	24.90	-5.93	0.01
C15	32.43	33.64	30.00	-3.64	0.29
C4	42.03	42.98	42.60	-0.38	0.73
C13	42.41	43.35	43.40	0.05	0.55
C1	48.71	49.48	48.60	-0.88	0.44
C3	51.39	52.09	49.40	-2.69	0.13
C5	52.44	53.11	53.10	-0.01	0.69
C9	56.62	57.17	78.30	21.13	0.01
C2	62.84	63.22	83.40	20.18	0.01
C6	216.89	213.10	207.20	-5.90	0.02
C14	219.07	215.22	208.70	-6.52	0.01

Incorrect isomer

label	calc	corrected	exp	error	prob
C9	21.14	19.30	18.70	-0.60	0.50
C8	21.89	20.02	20.80	0.78	0.56
C16	26.14	24.10	24.90	0.80	0.57
C12	31.86	29.58	30.00	0.42	0.69
C10	44.32	41.53	42.60	1.07	0.67
C4	49.34	46.35	43.40	-2.95	0.58
C5	51.79	48.69	48.60	-0.09	0.73
C1	52.38	49.26	49.40	0.14	0.72
C3	56.83	53.53	53.10	-0.43	0.72
C14	80.56	76.29	78.30	2.01	0.53
C2	89.21	84.58	83.40	-1.18	0.20
C11	217.45	207.54	207.20	-0.34	0.65
C6	218.27	208.33	208.70	0.37	0.71

Correct Isomer

7.5 S5

label	calc	corrected	exp	error	prob
C22	23.89	20.62	28.50	7.88	0.08
C19	30.99	27.63	30.50	2.87	0.65
C18	39.17	35.70	36.50	0.80	0.71
C26	58.59	54.88	42.40	-12.48	0.01
C3	60.59	56.86	60.80	3.94	0.23
C6	72.41	68.53	61.70	-6.83	0.01
C4	76.75	72.82	71.80	-1.02	0.71
C5	88.09	84.01	81.70	-2.31	0.71
C1	122.59	118.08	122.10	4.02	0.18
C16	126.30	121.74	127.30	5.56	0.07
C10	127.23	122.66	127.40	4.74	0.08
C15	128.03	123.44	129.90	6.46	0.01
C12	142.15	137.39	137.00	-0.39	0.72
C14	142.73	137.96	142.40	4.44	0.06
C13	158.05	153.08	145.60	-7.48	0.01
C7	160.52	155.53	145.80	-9.73	0.01
C9	164.01	158.97	158.10	-0.87	0.46
C11	165.06	160.01	158.70	-1.31	0.12
C2	176.12	170.93	173.50	2.57	0.70
C17	213.25	207.59	206.70	-0.89	0.59

Incorrect isomer

label	calc	corrected	exp	error	prob
C17	32.92	29.60	28.50	-1.10	0.21
C22	35.91	32.52	30.50	-2.02	0.44
C16	39.26	35.80	36.50	0.70	0.73
C3	49.84	46.13	42.40	-3.73	0.35
C26	57.20	53.33	60.80	7.47	0.01
C6	66.06	61.98	61.70	-0.28	0.72
C4	78.11	73.77	71.80	-1.97	0.18
C5	81.41	76.99	81.70	4.71	0.10
C1	129.42	123.91	122.10	-1.81	0.31
C14	129.95	124.43	127.30	2.87	0.69
C13	132.32	126.75	127.40	0.65	0.69
C9	137.37	131.69	129.90	-1.79	0.15
C12	141.69	135.90	137.00	1.10	0.18
C2	149.71	143.75	142.40	-1.35	0.07
C7	152.50	146.47	145.60	-0.87	0.42
C18	154.89	148.81	145.80	-3.01	0.57
C10	164.00	157.71	158.10	0.39	0.51
C11	168.15	161.76	158.70	-3.06	0.21
C8	178.33	171.71	173.50	1.79	0.19
C15	212.77	205.38	206.70	1.32	0.08

Correct Isomer

7.6 S6

label	calc	corrected	exp	error	prob
C19	14.08	11.26	14.46	3.20	0.26
C14	19.25	15.67	18.08	2.41	0.73
C4	25.98	21.41	20.89	-0.52	0.73
C17	28.95	23.94	22.05	-1.89	0.17
C16	31.69	26.28	26.22	-0.06	0.67
C5	31.93	26.48	27.07	0.59	0.66
C9	33.90	28.16	28.74	0.58	0.70
C6	37.92	31.59	35.47	3.88	0.19
C11	51.35	43.04	41.16	-1.88	0.24
C3	52.06	43.64	42.26	-1.38	0.09
C10	52.78	44.26	51.02	6.76	0.01
C13	60.78	51.08	57.80	6.72	0.01
C1	84.57	71.36	64.86	-6.50	0.01
C2	116.09	98.23	76.90	-21.33	0.01
C7	144.68	122.61	136.08	13.47	0.01
C8	172.07	145.97	141.92	-4.05	0.08

Incorrect isomer

label	calc	corrected	exp	error	prob
C16	16.57	14.81	14.46	-0.35	0.72
C14	19.25	17.31	18.08	0.77	0.45
C12	21.39	19.30	20.89	1.59	0.09
C8	25.60	23.23	22.05	-1.18	0.12
C13	26.88	24.43	26.22	1.79	0.23
C2	30.75	28.04	27.07	-0.97	0.12
C7	31.58	28.81	28.74	-0.07	0.65
C6	40.95	37.56	35.47	-2.09	0.39
C3	45.92	42.19	41.16	-1.03	0.16
C11	47.37	43.54	42.26	-1.28	0.08
C9	55.84	51.45	51.02	-0.43	0.48
C17	58.50	53.92	57.80	3.88	0.10
C1	71.63	66.17	64.86	-1.31	0.54
C10	81.86	75.71	76.90	1.19	0.28
C4	146.61	136.12	136.08	-0.04	0.65
C5	153.34	142.40	141.92	-0.48	0.65

Correct Isomer

7.7 S7

label	calc	corrected	exp	error	prob
C27	20.38	22.14	26.60	4.46	0.08
C12	24.90	25.90	27.30	1.40	0.36
C11	25.16	26.12	30.90	4.78	0.05
C4	48.77	45.79	36.10	-9.69	0.01
C3	51.29	47.88	45.70	-2.18	0.64
C1	51.92	48.41	47.00	-1.41	0.48
C2	72.07	65.19	65.30	0.11	0.71
C8	103.29	91.20	86.90	-4.30	0.14
C6	115.88	101.69	112.50	10.81	0.01
C5	208.87	179.15	171.50	-7.65	0.01
C7	209.70	179.83	183.50	3.67	0.07

Incorrect isomer

label	calc	corrected	exp	error	prob
C12	27.96	25.41	26.60	1.19	0.11
C11	28.91	26.33	27.30	0.97	0.26
C13	31.36	28.68	30.90	2.22	0.72
C6	42.77	39.64	36.10	-3.54	0.13
C3	48.42	45.08	45.70	0.62	0.02
C1	49.75	46.35	47.00	0.65	0.20
C2	72.54	68.27	65.30	-2.97	0.58
C4	91.38	86.38	86.90	0.52	0.24
C7	119.54	113.45	112.50	-0.95	0.20
C8	176.51	168.22	171.50	3.28	0.60
C5	194.47	185.48	183.50	-1.98	0.14

Correct Isomer

7.8 S8

label	calc	corrected	exp	error	prob
C8	36.64	20.92	32.40	11.48	0.01
C5	82.56	68.06	66.20	-1.86	0.73
C6	83.37	68.88	66.70	-2.18	0.49
C4	88.00	73.64	71.90	-1.74	0.72
C2	152.81	140.15	130.90	-9.25	0.01
C3	163.37	151.00	135.50	-15.50	0.01
C1	168.09	155.84	174.90	19.06	0.01

Incorrect isomer

label	calc	corrected	exp	error	prob
C3	35.53	31.07	32.40	1.33	0.29
C6	69.55	65.11	66.20	1.09	0.26
C2	70.85	66.41	66.70	0.29	0.66
C1	75.63	71.20	71.90	0.70	0.72
C4	136.97	132.59	130.90	-1.69	0.17
C5	148.46	144.08	135.50	-8.58	0.01
C7	172.40	168.05	174.90	6.85	0.07

Correct Isomer

7.9 S9

label	calc	corrected	exp	error	prob
C21	10.81	7.07	10.30	3.23	0.27
C20	31.26	27.56	27.00	-0.56	0.72
C13	39.38	35.69	34.80	-0.89	0.51
C7	40.49	36.80	39.00	2.20	0.55
C4	40.75	37.07	39.20	2.13	0.48
C8	65.89	62.25	48.80	-13.45	0.01
C5	65.98	62.34	49.20	-13.14	0.01
C2	74.40	70.78	79.50	8.72	0.01
C3	74.60	70.98	80.10	9.12	0.01
C9	82.50	78.89	80.20	1.31	0.11
C16	82.87	79.27	82.70	3.43	0.22
C6	84.06	80.46	86.70	6.24	0.01
C17	91.15	87.56	89.00	1.44	0.48
C15	116.15	112.60	111.40	-1.20	0.65
C14	152.16	148.68	140.10	-8.58	0.01

Incorrect isomer

label	calc	corrected	exp	error	prob
C20	11.20	12.47	10.30	-2.17	0.65
C19	29.72	29.57	27.00	-2.57	0.24
C9	31.32	31.05	34.80	3.75	0.18
C10	33.83	33.37	39.00	5.63	0.08
C2	40.53	39.55	39.20	-0.35	0.60
C13	41.04	40.03	48.80	8.77	0.01
C14	69.68	66.48	49.20	-17.28	0.01
C1	83.01	78.80	79.50	0.70	0.69
C11	84.04	79.74	80.10	0.36	0.61
C5	84.23	79.92	80.20	0.28	0.48
C8	85.67	81.25	82.70	1.45	0.50
C6	90.86	86.04	86.70	0.66	0.68
C15	91.42	86.56	89.00	2.44	0.27
C4	114.94	108.29	111.40	3.11	0.43
C3	154.57	144.89	140.10	-4.79	0.06

Correct Isomer

7.10 S10

label	calc	corrected	exp	error	prob
C14	18.74	19.30	22.00	2.70	0.66
C21	24.53	24.83	22.07	-2.76	0.57
C15	27.21	27.40	22.45	-4.95	0.07
C20	32.25	32.21	22.73	-9.48	0.01
C7	34.63	34.49	28.53	-5.96	0.06
C4	41.47	41.03	41.73	0.70	0.62
C3	45.14	44.54	54.37	9.83	0.01
C2	46.81	46.14	54.48	8.34	0.06
C5	51.86	50.97	62.57	11.60	0.01
C6	58.73	57.53	62.77	5.24	0.05
C1	85.75	83.37	84.12	0.75	0.40
C17	145.48	140.49	130.94	-9.55	0.01
C19	150.99	145.76	135.07	-10.69	0.01
C9	188.85	181.96	177.27	-4.69	0.11
C16	210.49	202.66	211.58	8.92	0.01

Incorrect isomer

label	calc	corrected	exp	error	prob
C19	22.82	20.04	22.00	1.96	0.25
C20	23.41	20.61	22.07	1.46	0.06
C12	25.34	22.46	22.45	-0.01	0.50
C14	25.79	22.88	22.73	-0.15	0.72
C13	32.78	29.58	28.53	-1.05	0.36
C1	47.75	43.92	41.73	-2.19	0.61
C5	58.19	53.92	54.37	0.45	0.32
C4	58.25	53.98	54.48	0.50	0.17
C8	66.93	62.29	62.57	0.28	0.68
C2	67.99	63.31	62.77	-0.54	0.26
C3	88.56	83.02	84.12	1.10	0.09
C11	141.85	134.05	130.94	-3.11	0.31
C10	144.36	136.46	135.07	-1.39	0.04
C7	185.40	175.78	177.27	1.49	0.12
C9	221.54	210.39	211.58	1.19	0.22

Correct Isomer

7.11 S11

label	calc	corrected	exp	error	prob
C10	29.80	18.50	24.50	6.00	0.01
C11	33.34	22.29	26.40	4.11	0.18
C9	52.43	42.67	46.20	3.53	0.18
C12	72.68	64.29	49.60	-14.69	0.01
C5	132.66	128.35	127.10	-1.25	0.28
C1	132.82	128.52	128.30	-0.22	0.65
C4	133.01	128.72	128.30	-0.42	0.63
C2	134.28	130.08	129.80	-0.28	0.68
C6	136.01	131.93	129.80	-2.13	0.54
C3	139.78	135.95	137.30	1.35	0.53
C7	167.74	165.80	169.80	4.00	0.48

Incorrect isomer

label	calc	corrected	exp	error	prob
C11	28.13	24.39	24.50	0.11	0.63
C10	30.46	26.68	26.40	-0.28	0.65
C12	50.60	46.55	46.20	-0.35	0.72
C9	53.16	49.08	49.60	0.52	0.72
C3	131.95	126.81	127.10	0.29	0.69
C4	132.30	127.16	128.30	1.14	0.17
C1	133.52	128.36	128.30	-0.06	0.45
C6	135.25	130.07	129.80	-0.27	0.61
C2	135.59	130.40	129.80	-0.60	0.60
C5	143.11	137.82	137.30	-0.52	0.72
C7	175.50	169.78	169.80	0.02	0.67

Correct Isomer

7.12 S12

label	calc	corrected	exp	error	prob
C18	70.89	47.62	40.40	-7.22	0.01
C20	76.14	54.54	53.30	-1.24	0.21
C1	79.16	58.52	65.10	6.58	0.18
C3	80.60	60.41	65.80	5.39	0.07
C6	86.50	68.19	69.50	1.31	0.31
C2	87.33	69.28	78.40	9.12	0.05
C11	95.19	79.63	81.00	1.37	0.10
C26	132.69	129.03	104.90	-24.13	0.01
C22	132.69	129.04	129.50	0.46	0.62
C24	132.93	129.35	130.60	1.25	0.27
C25	133.21	129.73	131.40	1.67	0.40
C23	133.64	130.29	134.20	3.91	0.16
C21	145.67	146.14	167.90	21.76	0.01
C12	178.85	189.84	169.60	-20.24	0.01

Incorrect isomer

label	calc	corrected	exp	error	prob
C10	66.92	46.32	40.40	-5.92	0.07
C1	69.63	49.77	53.30	3.53	0.41
C2	71.79	52.50	65.10	12.60	0.01
C12	75.97	57.81	65.80	7.99	0.07
C4	82.20	65.72	69.50	3.78	0.16
C8	83.49	67.35	78.40	11.05	0.01
C5	108.46	99.05	81.00	-18.05	0.01
C18	133.25	130.51	104.90	-25.61	0.01
C16	133.66	131.03	129.50	-1.53	0.61
C14	133.67	131.05	130.60	-0.45	0.69
C17	133.75	131.15	131.40	0.25	0.70
C15	133.80	131.21	134.20	2.99	0.28
C13	143.02	142.92	167.90	24.98	0.01
C37	176.34	185.20	169.60	-15.60	0.01

Correct Isomer

7.13 S13

label	calc	corrected	exp	error	prob
C36	55.75	61.68	61.50	-0.18	0.63
C4	114.60	113.70	117.40	3.70	0.18
C19	119.16	117.73	118.20	0.47	0.67
C20	120.04	118.51	118.50	-0.01	0.70
C6	123.45	121.52	119.90	-1.62	0.18
C2	125.63	123.45	120.20	-3.25	0.39
C5	128.09	125.63	127.40	1.77	0.21
C7	136.37	132.94	130.20	-2.74	0.47
C18	141.28	137.28	138.50	1.22	0.16
C17	144.53	140.16	145.00	4.84	0.03
C3	160.99	154.71	150.50	-4.21	0.07

Incorrect isomer

label	calc	corrected	exp	error	prob
C16	63.02	54.85	61.50	6.65	0.01
C1	124.73	113.54	117.40	3.86	0.69
C3	129.98	118.53	117.40	-1.13	0.25
C9	130.67	119.19	118.20	-0.99	0.15
C11	137.81	125.98	119.00	-6.98	0.08
C4	138.11	126.27	119.90	-6.37	0.01
C12	140.27	128.33	120.20	-8.13	0.01
C10	140.85	128.88	127.40	-1.48	0.06
C2	142.30	130.26	130.20	-0.06	0.59
C5	148.55	136.20	138.50	2.30	0.69
C13	154.16	141.54	145.00	3.46	0.72
C6	158.54	145.71	150.50	4.79	0.17
C8	159.28	146.41	150.50	4.09	0.06

Correct Isomer

References

[1] A. Howarth, K. Ermanis and J. M. Goodman, *Chemical Science*, 2020, **11**, 4351–4359.

[2] K. Ermanis, K. E. B. Parkes, T. Agback and J. M. Goodman, *Organic & Biomolecular Chemistry*, 2019, **17**, 5886–5890.

[3] K. Ermanis, K. E. B. Parkes, T. Agback and J. M. Goodman, *Organic & Biomolecular Chemistry*, 2016, **14**, 3943–3949.

[4] K. Ermanis, K. E. B. Parkes, T. Agback and J. M. Goodman, *Organic & Biomolecular Chemistry*, 2017, **15**, 8998–9007.

[5] *MacroModel, version 9.9*, Schrödinger, LLC, New York, NY, 2009.

[6] A. D. Becke, *Physical Review A*, 1988, **38**, 3098–3100.

[7] C. Lee, W. Yang and R. G. Parr, *Physical Review B*, 1988, **37**, 785–789.

[8] M. J. Frisch, G. W. Trucks, H. B. Schlegel, G. E. Scuseria, M. A. Robb, J. R. Cheeseman, G. Scalmani, V. Barone, B. Mennucci, G. A. Petersson, H. Nakatsuji, M. Caricato, X. Li, H. P. Hratchian, A. F. Izmaylov, J. Bloino, G. Zheng, J. L. Sonnenberg, M. Hada, M. Ehara, K. Toyota, R. Fukuda, J. Hasegawa, M. Ishida, T. Nakajima, Y. Honda, O. Kitao, H. Nakai, T. Vreven, J. A. Montgomery, Jr., J. E. Peralta, F. Ogliaro, M. Bearpark, J. J. Heyd, E. Brothers, K. N. Kudin, V. N. Staroverov, R. Kobayashi, J. Normand, K. Raghavachari, A. Rendell, J. C. Burant, S. S. Iyengar, J. Tomasi, M. Cossi, N. Rega, J. M. Millam, M. Klene, J. E. Knox, J. B. Cross, V. Bakken, C. Adamo, J. Jaramillo, R. Gomperts, R. E. Stratmann, O. Yazyev, A. J. Austin, R. Cammi, C. Pomelli, J. W. Ochterski, R. L. Martin, K. Morokuma, V. G. Zakrzewski, G. A. Voth, P. Salvador, J. J. Dannenberg, S. Dapprich, A. D. Daniels, . Farkas, J. B. Foresman, J. V. Ortiz, J. Cioslowski and D. J. Fox, *Gaussian 09 Revision D.01*, Gaussian Inc. Wallingford, CT, 2009.

[9] L. Lagardère, L. H. Jolly, F. Lipparini, F. Aviat, B. Stamm, Z. F. Jing, M. Harger, H. Torabifard, G. A. Cisneros, M. J. Schnieders, N. Gresh, Y. Maday, P. Y. Ren, J. W. Ponder and J. P. Piquemal, *Chemical Science*, 2018, **9**, 956–972.

[10] Y. Zhao and D. G. Truhlar, *Theoretical Chemistry Accounts*, 2008, **120**, 215–241.

[11] F. Weigend, *Physical Chemistry Chemical Physics*, 2006, **8**, 1057.

[12] F. Weigend and R. Ahlrichs, *Physical Chemistry Chemical Physics*, 2005, **7**, 3297.

[13] F. London, *Journal de Physique et le Radium*, 1937, **8**, 397–409.

[14] C. Adamo and V. Barone, *The Journal of Chemical Physics*, 1998, **108**, 664–675.

[15] J. Li, J. K. Liu and W. X. Wang, *Journal of Organic Chemistry*, 2020, **85**, 11350–11358.

[16] A. S. Christensen, L. A. Bratholm, F. A. Faber and O. Anatole Von Lilienfeld, *Journal of Chemical Physics*, 2020, **152**, 044107.

[17] S. Kuhn and N. E. Schlörer, *Magnetic Resonance in Chemistry*, 2015, **53**, 582–589.

[18] Y. Guan, S. V. S. Sowndarya, L. C. Gallegos, P. C. S. John and R. S. Paton, *Chemical Science*, 2021.

[19] W. Gerrard, L. A. Bratholm, M. J. Packer, A. J. Mulholland, D. R. Glowacki and C. P. Butts, *Chemical Science*, 2020, **11**, 508–515.

[20] K. M. A.S. Christensen, F. A. Faber, B. Huang, L.A. Bratholm, A. Tkatchenko and O. Anatole Von Lilienfeld, *GitHub repository*, 2017.

[21] J. R. Cabrera-Pardo, A. Trowbridge, M. Nappi, K. Ozaki and M. J. Gaunt, *Angewandte Chemie International Edition*, 2017, **56**, 11958–11962.

[22] K. F. Hogg, A. Trowbridge, A. Alvarez-Pérez and M. J. Gaunt, *Chemical Science*, 2017, **8**, 8198–8203.

[23] B. Y. Han, N. Y. Lam, C. I. MacGregor, J. M. Goodman and I. Paterson, *Chemical Communications*, 2018, **54**, 3247–3250.

[24] L. B. Marx and J. W. Burton, *Chemistry - A European Journal*, 2018, **24**, 6747–6754.

[25] S. Ainsua Martinez, M. Gillard, A. C. Chany and J. W. Burton, *Tetrahedron*, 2018, **74**, 5012–5021.

[26] N. Y. S. Lam, G. Muir, V. R. Challa, R. Britton and I. Paterson, *Chemical Communications*, 2019, **55**, 9717–9720.
